# Hijacking of host PCNA by circovirus replication-associated protein to recruit POLD1 drives viral DNA replication and is inhibited by R428

**DOI:** 10.1371/journal.ppat.1014575

**Published:** 2026-09-28

**Authors:** Lei Zhu, Guan Zhang, Yingying Pu, Wenyu Wang, Zhanyan Zhang, Jiasai Kang, Haoshu Zhang, Lingling Chang, Le Shi, Yong Huang, Dewen Tong, Qian Du

**Affiliations:** 1 College of Veterinary Medicine, Northwest A&F University, Yangling, China; 2 Center for Mitochondrial Biology and Medicine, The Key Laboratory of Biomedical Information Engineering of Ministry of Education, School of Life Science and Technology, Xi’an Jiaotong University, Xi’an, China; 3 Engineering Research Center of Efficient New Vaccines for Animals, Ministry of Education, Yangling, China; 4 Key Laboratory of Ruminant Disease Prevention and Control (West), Ministry of Agriculture and Rural Affairs, Yangling, China; 5 Engineering Research Center of Efficient New Vaccines for Animals, Universities of Shaanxi Province, Yangling, China; University of Pittsburgh School of Medicine, UNITED STATES OF AMERICA

## Abstract

Circoviruses are the smallest known mammalian viruses with a single-stranded circular genome, whose replication capacity is highly dependent on host factors. Although the virus-encoded replication-associated protein (Rep/Rep’) plays a critical role in circovirus DNA replication, it lacks polymerase activity. Here, we uncovered a mechanism by which circoviruses hijack the host proliferating cell nuclear antigen (PCNA) via their Rep to recruit POLD1, the catalytic subunit of host DNA polymerase δ, thereby driving viral DNA synthesis. POLD1 was identified to be the major polymerase in the replication of circovirus DNA, and the virus infection promoted POLD1 expression. Further, it had been found that the Rep of Circoviruses bound to host sliding clamp protein PCNA to indirectly recruit POLD1 to viral replication centers, and the N-terminal motif LIXXG of Rep and the C-terminal motif NXNXK of PCNA were identified to be corresponding conservative binding sites. As PCV2 is the most clinically significant circovirus, a small-molecule drug R428 was screened to effectively block the binding between PCV2 Rep and porcine PCNA. R428 could significantly suppress the formation of the Rep/PCNA complex in cells, and effectively suppress PCV2 replication and alleviate PCV2 infection-induced tissue damage in mice and piglets. In conclusion, we elucidated the novel mechanism by which circoviruses hijack host PCNA-POLD1 to facilitate their DNA replication via Rep, and identified R428 as a potential anti-PCV2 agent for the prevention and control of circovirus-associated diseases.

## Author summary

Circoviruses are major economic pathogens in the animal industry and pose a potential threat to humans. The widely used porcine circovirus type 2 (PCV2) vaccines cannot fully prevent virus spread, and no vaccines are currently available for other circoviruses. Therefore, elucidating their replication mechanism may provide suitable strategies for the prevention and control of these viruses. Considering that circoviruses rely on the host enzyme system for replication, we screened host polymerases involved in circovirus DNA replication and found that POLD1, the catalytic subunit of host DNA polymerase δ, is the major polymerase involved. Further research revealed that circoviruses hijack host proliferating cell nuclear antigen (PCNA) via their replication-associated protein (Rep) to recruit POLD1, and the N-terminal motif LIXXG of Rep and the C-terminal motif NXNXK of PCNA were identified as the conserved binding sites. Based on these findings, a small-molecule drug, R428, was found to be effective in blocking the binding between PCV2 Rep and porcine PCNA. In summary, this study revealed that circoviruses utilize Rep to hijack host POLD1 through PCNA, thereby promoting their DNA replication, and identified R428 as a potential anti-PCV2 agent for controlling circovirus-associated diseases.

## Introduction

Circoviruses belong to the genus *Circovirus* within the family *Circoviridae* and are among the smallest mammalian viruses with single-stranded, circular DNA genomes of approximately 1.6 to 2.2 kb [1–3]. The non-pathogenic porcine circovirus type 1 (PCV1) is the first member of this family, and was identified in 1974 [4]. The discovery of the pathogenic PCV2 and its association with postweaning multisystemic wasting syndrome (PMWS) in 1996 revolutionized the understanding of this viral family, establishing it as a significant agricultural pathogen [5]. Subsequently, additional porcine circovirus genotypes were identified, and new circoviruses were detected in avian, canine, and other hosts, many of which are associated with clinical disease [6–8]. More recently, the discovery of circovirus-like DNA sequences in human samples from unexplained fever and hepatitis cases has raised important questions about their potential as zoonotic agents and human pathogens [9–12]. Given that PCV2 and beak and feather disease virus (BFDV), two well-known circoviruses, share features of immune impairment and persistent infection [5,13], understanding the molecular mechanisms that underpin the circovirus life cycle is essential for developing new prevention and control strategies. The genome of circoviruses contains two major open reading frames (ORFs), ORF1 and ORF2. ORF2 encodes the capsid protein (Cap), the sole structural component, which is essential for viral assembly and immunogenicity [14,15]. ORF1 encodes the non-structural replication-associated protein (Rep) and its splice variant Rep’, which are the master regulators of viral genome replication [16]. The replication of circoviruses’ genomic DNA initiates when Rep binds to a specific sequence within the intergenic region [17]. Upon binding, the endonuclease, or nickase, domain of Rep introduces a site-specific single-strand nick that initiates rolling-circle replication (RCR), during which the replication machinery synthesizes a new positive strand using the circular negative strand as a template [18,19]. Although the nicking and ligation activities of Rep are well characterized, its lack of polymerase activity creates a critical dependence on host cells, requiring the virus to commandeer cellular factors for high-fidelity synthesis of new viral genomes. The identities of these essential host factors and the mechanisms by which Rep recruits them to the viral origin of replication have, until now, remained poorly understood.

In eukaryotic cells, genome duplication is orchestrated by a sophisticated and tightly regulated multiprotein complex known as the replisome [20]. At the core of this machinery are several distinct DNA polymerases, each with specialized functions. DNA polymerase α (Pol α)-primase complex initiates synthesis by laying down a short RNA-DNA hybrid primer [21]. The main replicative workload is then handed over to the highly processive DNA polymerases δ (Pol δ) and ε (Pol ε), which are primarily responsible for lagging and leading strand synthesis, respectively [22–24]. The high processivity of these polymerases also depends on several auxiliary factors. Specifically, these polymerases achieve efficient synthesis by binding to a ring-shaped sliding clamp protein, such as proliferating cell nuclear antigen (PCNA), which is loaded onto primer-template junctions by the replication factor C (RFC) complex and then encircles the DNA to tether DNA polymerases and other replication factors to the template as a mobile platform [25–27]. This network of protein-protein interactions at the replication fork ensures the coordinated and rapid duplication of the host genome [28]. Some DNA viruses have been shown to hijack host polymerase to replicate their genomes. For instance, the replication of adeno-associated virus (AAV), another small DNA virus, is known to be dependent on a suite of host factors that includes replication protein A (RPA), replication factor (RFC), proliferating cell nuclear antigen (PCNA), and DNA polymerase delta 1 (POLD1) [29]. These precedents strongly suggest that circoviruses may have evolved mechanisms to similarly tap into this powerful host machinery.

In this study, we bridge the gap in our understanding of circovirus replication and address the urgent need for new antiviral strategies. We first combined DNA pull-down with LC-MS/MS assay approach to identify host factors associated with circovirus replication, leading to the discovery that circovirus DNA binds to POLD1. Through molecular and cellular analyses, we uncovered an evolutionarily conserved mechanism in which circovirus Rep interacts with host PCNA, which then serves as a scaffold to recruit the POLD1-containing Pol δ holoenzyme to the viral replication origin. Based on AlphaFold3 structural prediction of the Rep-PCNA complex, we identified a druggable pocket at the protein-protein interface. Using this structural information, we performed in silico screening followed by experimental validation and identified R428 as a small molecule that disrupts the Rep-PCNA interaction and shows antiviral activity against PCV2 in cell culture and animal models. While the efficacy of R428 against other circoviruses requires further investigation, our findings provide a robust proof-of-concept for a new therapeutic paradigm. This research provides a critical theoretical foundation and a promising new avenue for the control of diseases caused by the circovirus family.

## Results

### Circoviruses employ host POLD1 to replicate their genomic DNA

To identify the host DNA polymerase used by circoviruses to replicate their single-stranded circular DNA, we used PCV2 as a prototypical virus for the initial screening. Biotin-labeled PCV2 DNA or biotin-labeled non-coding DNA was transfected into PK-15 cells for 12 h, after which PCV2 DNA-associated host proteins were enriched with streptavidin-coated beads and analyzed by LC-MS/MS (Fig 1A). Seven host DNA polymerase subunits were identified (S1 Table). Among these candidates, shRNA knockdown assays showed that only POLD1 and POLA1 contributed to PCV2 DNA replication, while only the POLD1 transcript level was significantly higher in PCV2-infected cells than in mock-infected cells (Fig 1B and S1A Fig in S1 File), suggesting that POLD1 may be the major polymerase regulating PCV2 DNA replication. Further results showed that the rescue of POLD1 expression significantly promoted PCV2 DNA replication compared to the empty vector control in the endogenous POLD1 knocked down cells (S1B Fig in S1 File). Subsequently, DNA pull-down and ChIP assays were used to verify the interaction of POLD1 and PCV2 DNA, and both assays confirmed that POLD1 could interact with PCV2 DNA. (Fig 1C, and S1C Fig in S1 File). To figure out if POLD1 also interacts with other circovirus DNA, we transfected HEK293T cells with Flag-tagged POLD1 of different species (pig, pigeon, human, and canine) and cyclized PCV2, PiCV, HirCV, and CaCV DNA, respectively, and the ChIP results showed that the POLD1 of different species did interact with corresponding circovirus DNA (Fig 1D). To further confirm the role of POLD1 in regulating circovirus DNA replication, we transfected PK-15 cells with sh-POLD1 for 24 h, followed by PCV2 infection for another 24 h, and the results showed that the knockdown of POLD1 significantly reduced the TCID_50_ and DNA copy numbers of PCV2, as well as the genomic (CP) and replicative form (RFP) PCV2 DNA (Fig 1E, 1F, 1G, 1H and S1D Fig in S1 File). Further results showed that the overexpression of POLD1 in PK-15 cells significantly increased the TCID_50_ and DNA copy numbers of PCV2, as well as the CP and RFP PCV2 DNA (Fig 1I, 1J, 1K, 1L and S1E Fig in S1 File). These results indicate that the single-stranded circular DNA of circoviruses mainly interacts with host polymerase POLD1 to facilitate their replication.

**Fig 1:**
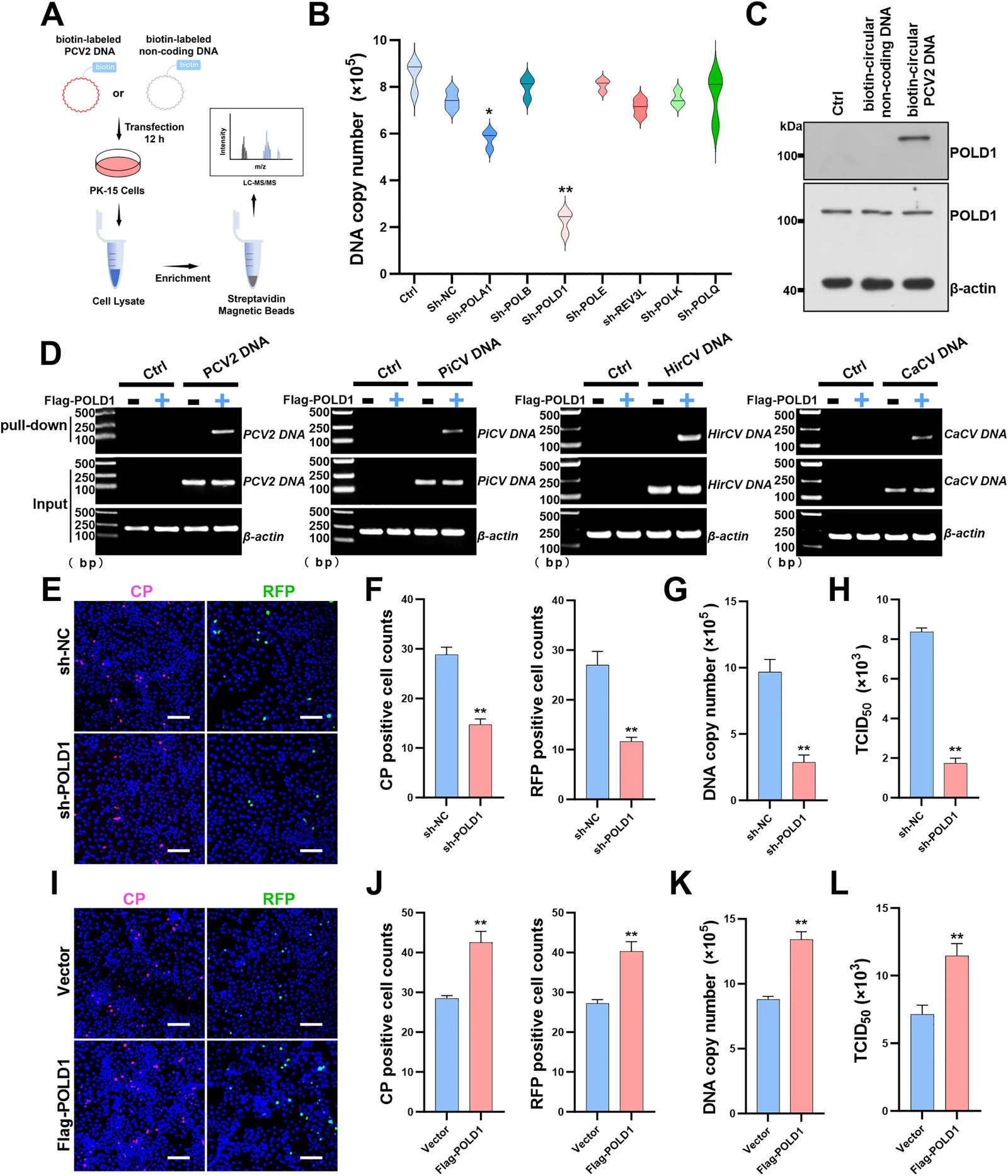
Interaction between circovirus DNA and POLD1. **(A)** Schematic diagram of the experiment design. biotin-labeled PCV2 DNA or biotin-labeled non-coding DNA was transfected into PK-15 cells for 12 **h.** PCV2 DNA-bound host proteins were extracted using streptavidin-coated beads and subsequently analyzed by liquid chromatography-tandem mass spectrometry (LC-MS/MS). (B) qPCR detection of the effect of interfering with the expression of different DNA polymerases on PCV2 DNA copy number; “Ctrl”represents cells that were infected with PCV2 alone. **p < 0.01 versus the Ctrl group. **(C)** PCV2 DNA pull-down of proteins detected by Western blot using an anti-POLD1 antibody; “Ctrl” represents untreated cells. **(D)** HEK293T cells were first transfected with Flag-tagged POLD1 from different species (pig, pigeon, human, and canine), and then transfected with cyclized PCV2, PiCV, HirCV, and CaCV DNA. Subsequently, chromatin immunoprecipitation (ChIP) was performed using Flag antibody to detect the interaction between POLD1 and the viral DNAs; “Ctrl” denotes non-coding DNA. (E, F, G, **H)** Effect of POLD1 interference on PCV2 replication. **(E)** Positive cells were photographed using a fluorescence microscope. Bars = 100 μm are indicated in each panel. **(F)** The counts of CP-positive and RFP-positive cells per 100 cells. **(G)** The DNA copy numbers of PCV2 were detected and calculated by qPCR. **(H)** The PCV2 TCID_50_ of these cells was measured and calculated. **p < 0.01 versus the sh-NC group in **(F)**, (G) and **(H)**. (I, J, K, **L)** Effect of POLD1 overexpression on PCV2 replication. **(I)** Positive cells were photographed using a fluorescence microscope. Bars = 100 μm are indicated in each panel. **(J)** The counts of CP-positive and RFP-positive cells per 100 cells. **(K)** The DNA copy numbers of PCV2 were detected and calculated by qPCR. **(L)** The PCV2 TCID_50_ of these cells was measured and calculated. **p < 0.01 versus the Vector group in **(J)**, (K) and **(L)**.

### Porcine circovirus infection upregulates host POLD1 expression both *in vitro* and *in vivo*

To investigate the impact of circovirus infection on POLD1 expression, we initially examined the expression levels of POLD1 in PCV2-infected PK-15 cells at different time points, and the results showed that PCV2 infection time-dependently upregulated both the mRNA and protein levels of POLD1 compared to mock infection in these cells (Fig 2A, 2B). Comparative analysis of POLD1 expression levels in various tissues (heart, liver, spleen, lung, kidney, and lymph nodes) from PCV2-infected and uninfected piglets showed significantly elevated POLD1 expression across all examined tissues in infected piglets (Fig 2C, 2D). Moreover, we found that the PCV2-targeted lymphoid tissues (spleens and lymph nodes) exhibited substantially higher POLD1 expression levels compared to other tissues (Fig 2C, 2D). Further, to determine whether POLD1 expression levels correlate with PCV2 loads in infected piglets, we detected POLD1 expression levels in spleens and lymph nodes from the PCV2-infected piglets, and the results showed that increased expression levels of POLD1 were positively associated with higher PCV2 titers in the spleens and lymph nodes of piglets (Fig 2E, 2F and S2A, 2B Fig in S1 File). These findings collectively demonstrate that PCV2 infection upregulates POLD1 expression levels in cells and tissues to promote viral DNA replication.

**Fig 2:**
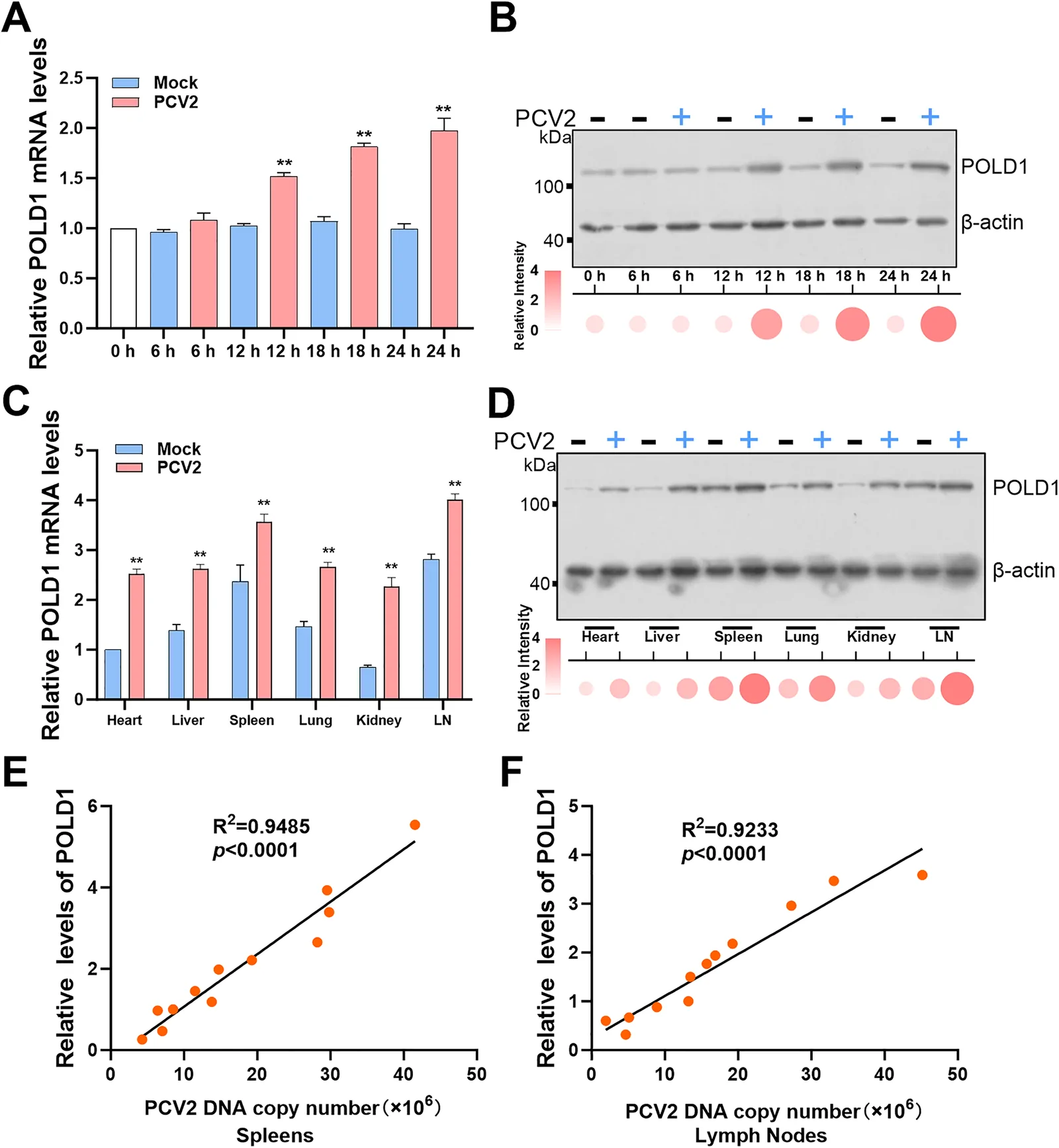
Effect of PCV2 infection on POLD1 expression. (A) qPCR analysis of POLD1 mRNA expression in PK-15 cells at different time points post-PCV2 infection. **(B)** Western blot analysis of POLD1 protein expression in PK-15 cells at different time points post-PCV2 infection. (C) qPCR analysis of POLD1 mRNA expression in various tissues of piglets following PCV2 infection. **(D)** Western blot analysis of POLD1 protein expression in various tissues of piglets following PCV2 infection. **(E)** Western blot analysis of POLD1 protein expression in the spleens of different piglets, and qPCR measurement of PCV2 viral loads in the corresponding spleen samples. **(F)** Western blot analysis of POLD1 protein expression in the lymph nodes of different piglets, and qPCR measurement of PCV2 viral loads in the corresponding lymph node samples. **p < 0.01 versus the Mock group in (A) and **(C)**.

### Circoviruses recruit host POLD1 through an indirect interaction of its replicase Rep

Since Rep is the major replication-associated protein encoded by circoviruses, we speculated that Rep plays an important role in recruiting POLD1 to facilitate viral DNA replication. In the PK-15 cells infected with PCV2 for 24 h, we found Rep and POLD1 were predominantly co-localized within the nucleus, and stronger POLD1 signals were detected in PCV2-infected cells than in mock-infected cells. (Fig 3A). Also, in the PCV2-infected PK-15 cells, we demonstrated that PCV2 Rep interacted with endogenous POLD1 using co-immunoprecipitation (co-IP) assays (Fig 3B and S3A Fig in S1 File). To determine whether Rep proteins from other circoviruses also interact with species-matched POLD1, we co-transfected HEK293T cells with GFP-tagged circovirus Rep constructs and Flag-tagged POLD1 orthologs. These assays confirmed interactions between Rep proteins from PCV2, PiCV, HirCV, and CaCV and the corresponding POLD1 proteins (Fig 3C and S3B, 3C Fig in S1 File). However, GST pull-down assays showed that none of these circovirus Rep proteins directly bound the corresponding POLD1 proteins (Fig 3D and S3D Fig in S1 File). Collectively, these findings suggest that circovirus Rep indirectly interacts with host POLD1 to recruit this polymerase.

**Fig 3:**
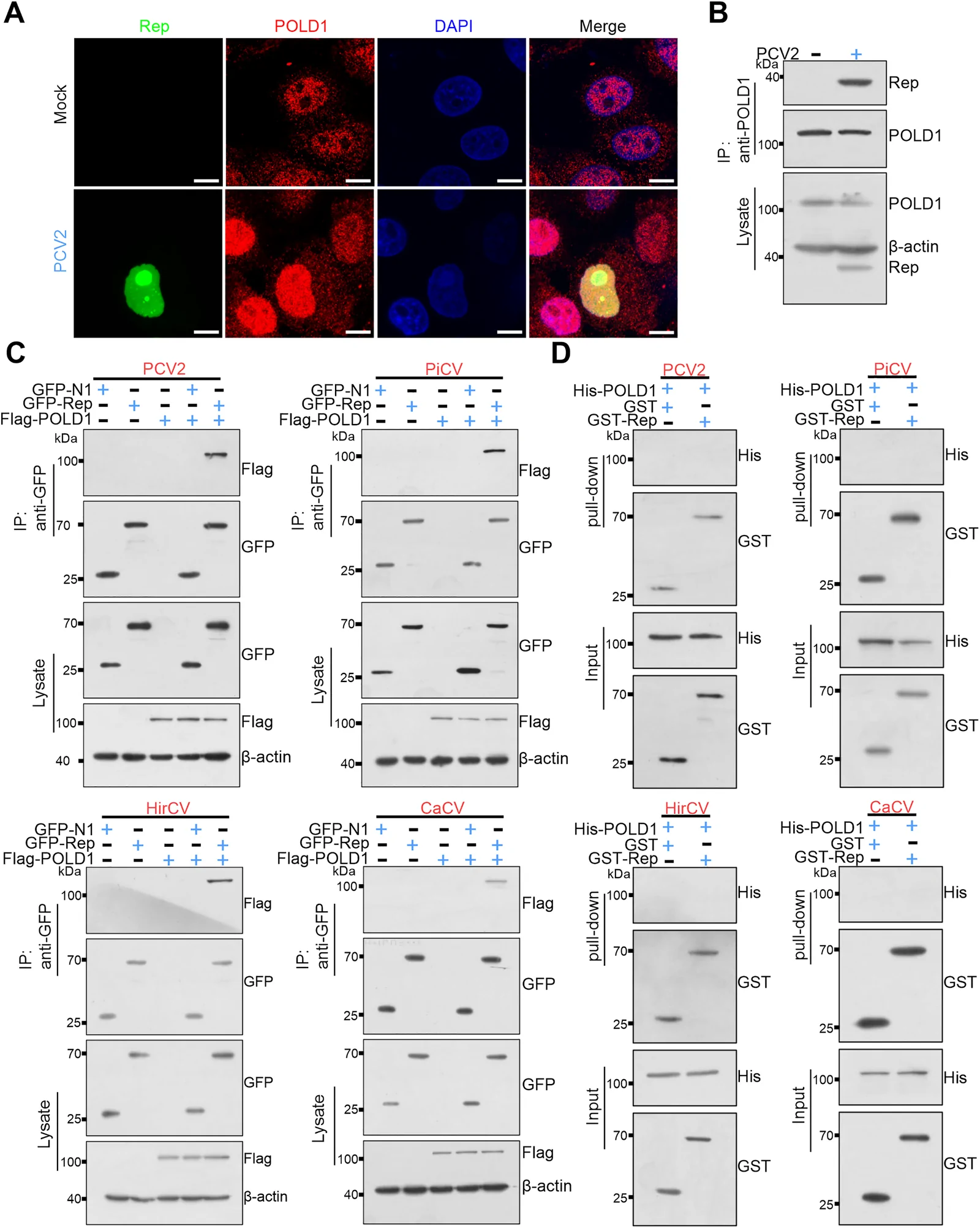
Circovirus Rep does not directly interact with POLD1. **(A)** The localizations of POLD1 was measured using confocal microscopy. The bars = 10 μm were indicated in each panel. **(B)** After PCV2 or mock infection of PK-15 cells for 24 h, co-IP assays to detect the interaction between POLD1 and the PCV2 Rep protein. (C) co-IP assays were performed to detect the interaction between exogenously expressed POLD1 and the exogenously expressed Rep proteins from PCV2, PiCV, HirCV, and CaCV. 293T cells were co-transfected with Flag-tagged POLD1 plasmid and each GFP-tagged Rep construct, with the empty GFP-N1 vector used as a negative control. Immunoprecipitation was carried out using anti-GFP magnetic beads, and the co-precipitated proteins were detected by Western blotting with an anti-Flag antibody. **(D)** GST pull-down assays were further performed to validate the direct interaction between POLD1 and the Rep proteins of PCV2, PiCV, HirCV, and CaCV. GST-Rep fusion proteins were immobilized on glutathione-Sepharose beads and incubated with in vitro-expressed His-POLD1. Bound proteins were detected by Western blotting using an anti-His antibody. GST alone (empty vector) served as a negative control.

### PCNA mediates the interaction of circovirus Rep and POLD1

It has been reported that POLD1 combines with other polymerase subunits to form a heterotetramer, Polδ, which uses proliferating cell nuclear antigen (PCNA) as a sliding clamp to initiate DNA synthesis during cell replication and repair [30]. Thus, we speculated that PCNA may mediate the interaction of circovirus Rep and POLD1. First, we found that PCV2 Rep and PCNA are also primarily co-localized in the nucleus of the infected cells, and PCV2 Rep interacted with endogenous and exogenous PCNA (Fig 4A, 4B, 4C, and S4A, 4B, 4C Fig in S1 File). Then, we checked the interaction between Rep of other circoviruses and PCNA of corresponding species, and the results showed that PiCV Rep, HirCV Rep, and CaCV Rep also interacted with their corresponding PCNA (Fig 4C and S4B, 4C Fig in S1 File). Further, we investigated that PCV2 Rep, PiCV Rep, HirCV Rep, and CaCV Rep all directly bond the corresponding PCNA proteins (Fig 4D and S4D Fig in S1 File). More importantly, we found that the presence of PCNA enabled PCV2 Rep, PiCV Rep, HirCV Rep, and CaCV Rep to interact with POLD1 in an *in vitro* system (Fig 4E and S4E Fig in S1 File). Furthermore, we observed that the knockdown of PCNA expression in PK-15 cells obviously reduced the interaction of PCV2 Rep and POLD1, and the PCV2 replication level was markedly reduced, as evidenced by decreased TCID₅₀, DNA copy numbers, as well as CP and RFP levels (Fig 4F, and S4F, S5A-5D Fig in S1 File). Finally, we performed a biotinylated DNA pull-down assay using wild-type PCV2 DNA or a Rep start-codon mutant as bait. Wild-type PCV2 DNA efficiently pulled down both POLD1 and PCNA, whereas the mutant PCV2 DNA failed to do so. However, when the mutant PCV2 DNA was co-transfected with a Rep-expressing plasmid, the pull-down of POLD1 and PCNA was restored (S5E, 5F Fig in S1 File). These results indicate that circovirus Rep universally recruits POLD1 via PCNA as a bridging factor to regulate viral replication, and Rep is necessary for recruiting the PCNA–POLD1 complex to viral DNA.

**Fig 4:**
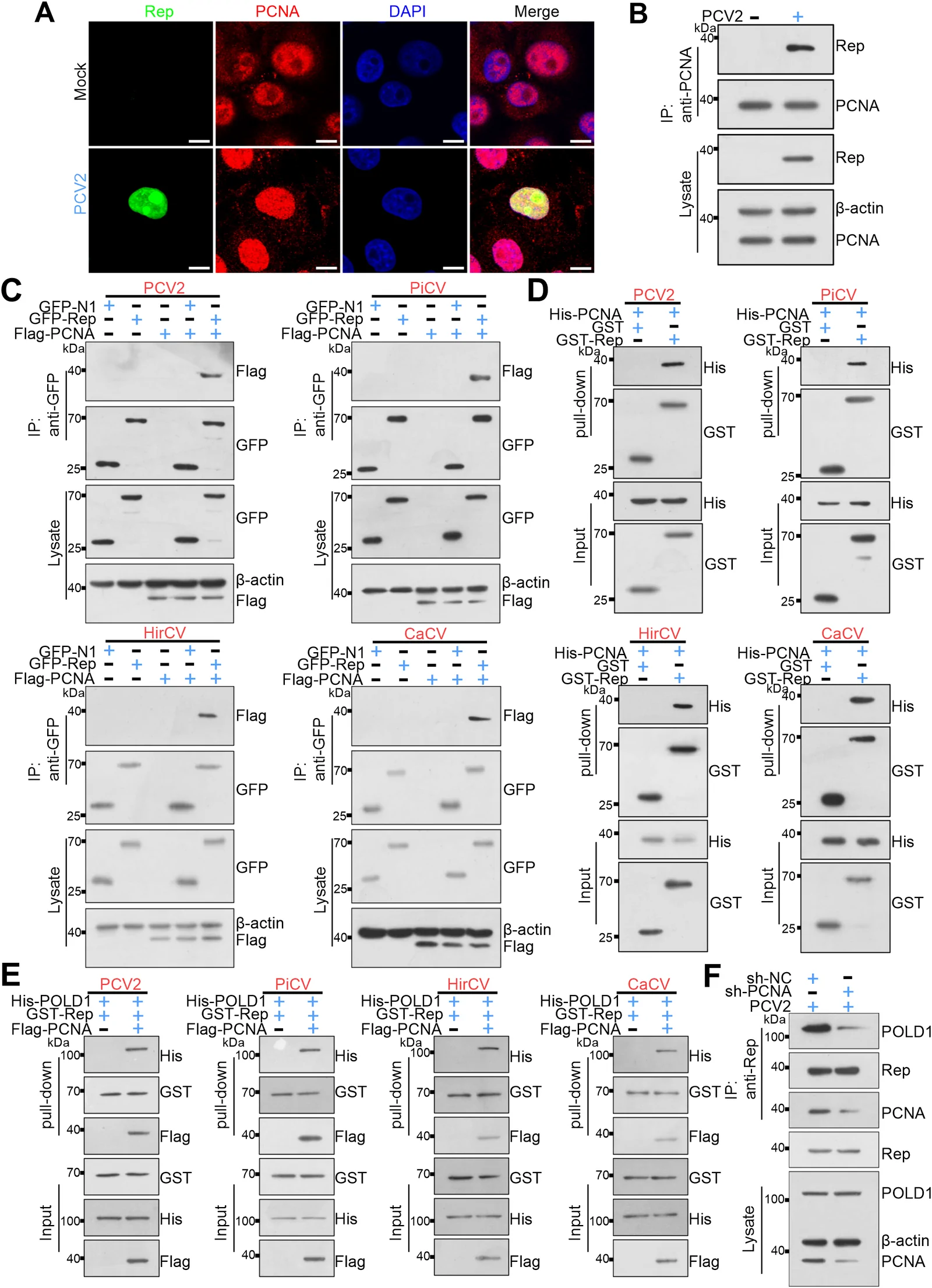
PCNA mediates the interaction of circovirus Rep and POLD1. **(A)** The localizations of PCNA were measured using confocal microscopy. The bars = 10 μm were indicated in each panel. **(B)** After PCV2 or mock infection of PK-15 cells for 24 h, co-IP assays to detect the interaction between PCNA and the PCV2 Rep protein. (C) co-IP assays were performed to detect the interaction between exogenously expressed PCNA and the exogenously expressed Rep proteins from PCV2, PiCV, HirCV, and CaCV. 293T cells were co-transfected with Flag-tagged PCNA plasmid and each GFP-tagged Rep construct, with the empty GFP-N1 vector used as a negative control. Immunoprecipitation was carried out using anti-GFP magnetic beads, and the co-precipitated proteins were detected by Western blotting with an anti-Flag antibody. **(D)** GST pull-down assays were further performed to validate the direct interaction between PCNA and the Rep proteins of PCV2, PiCV, HirCV, and CaCV. GST-Rep fusion proteins were immobilized on glutathione-Sepharose beads and incubated with in vitro-expressed His-PCNA. Bound proteins were detected by Western blotting using an anti-His antibody. GST alone (empty vector) served as a negative control. **(E)** GST pull-down assays to detect the effect of PCNA on the interaction between POLD1 and the Rep proteins of PCV2, PiCV, HirCV, and CaCV. GST-tagged Rep fusion proteins were immobilized on glutathione-Sepharose beads and incubated with in vitro-expressed His-POLD1 in the presence or absence of Flag-PCNA. After extensive washing, bound proteins were resolved by SDS-PAGE and detected by Western blotting using anti-His (for POLD1) and anti-Flag (for PCNA) antibodies. (F) co-IP assays to detect the effect of PCNA interference on the interaction between POLD1 and the PCV2 Rep protein. Immunoprecipitation was carried out using anti-Rep antibody, and the co-precipitated proteins were detected by Western blotting with an anti-POLD1 antibody.

### The LIXXG motif in the N-terminal domain of circovirus Rep interacts with the NXNXK motif on the C-terminal domain of PCNA

To identify the critical regions and amino acid residues mediating the interaction between circovirus Rep and PCNA, we first divided PCV2 Rep into five segments according to its functional domains (Fig 5A). These segments were co-transfected with a Flag-tagged PCNA into PK-15 cells, and the co-IP results showed that only the wild-type Rep, endonuclease domain (ED, named as R1), and ED-oligomerization domain (OD, named as R4) interacted with PCNA (Fig 5B and S6A Fig in S1 File). Next, we divided PCNA into two structural domains, N-terminal domain (PCNA-ND) and C-terminal domain (PCNA-CD) (Fig 5C). co-IP assays revealed that only PCNA-WT and PCNA-CD interacted with PCV2 Rep (Fig 5D and S6B Fig in S1 File). Further application of the AlphaFold3 software predicted that there were four potential interaction site-pairs between the PCV2 Rep endonuclease domain and the porcine PCNA C-terminal domain, namely R33, L104, I105, and G108 on Rep and E143, N177, N179, and K181 on PCNA, respectively (Fig 5E). Mutations were introduced at different sites of PCV2 Rep, and co-IP assays revealed that the mutation of R33 did not visibly affect the interaction of Rep and PCNA, while mutations in the other single residue (L104, I105, and G108) in PCV2 Rep reduced its interaction with porcine PCNA (Fig 5F and S6C, 6D, 6E Fig in S1 File). Moreover, simultaneous mutations of PCV2 Rep at residues L104, I105, and G108 resulted in a complete loss of its ability to bind porcine PCNA (Fig 5F and S6C, 6D, 6E Fig in S1 File). Similarly, the mutation of E143 did not clearly disturb the interaction of PCV2 Rep and PCNA, and the mutation of other single specific residue (N177, N179, and K181) in porcine PCNA did not totally eliminate its binding to PCV2 Rep, whereas simultaneous mutations of porcine PCNA at residues N177, N179, and K181 abolished its interaction with PCV2 Rep (Fig 5G and S6F, 6G Fig in S1 File). Subsequently, a PK-15 cell line with PCNA mutations (N177A-N179A-K181A), named PK-15^mPCNA^, was constructed (S6H, 6I Fig in S1 File). After infecting PK-15^WT^ and PK-15^mPCNA^ with PCV2 for 24 h, co-IP assay results showed that PCV2 Rep failed to bind to POLD1 in PK-15^mPCNA^, unlike in PK-15^WT^ (Fig 5H and S6J Fig in S1 File). Similarly, the *in vitro* pull-down assays showed that the mutant PCNA could not mediate the interaction of PCV2 Rep and POLD1 (Fig 5I). Further analysis results showed that the mutation of porcine PCNA significantly reduced the replication levels of PCV2 in PK-15 cells (Fig 5J-5M). To investigate whether the binding sites of circovirus Rep and their corresponding PCNA are conserved, we aligned the amino acid sequences of PCV2 Rep, PiCV Rep, HirCV Rep, and CaCV Rep, as well as PCNA from different species, and the results showed that all the Rep contain a conservative motif LIXXG, while all the PCNA contain the corresponding NXNXK motif, and all the Rep and PCNA were predicted to bind on these two conserved motifs (Fig 5N, 5O and S6K Fig in S1 File). These findings show that the LIXXG motif on the N-terminal domain of circovirus Rep and the NXNXK motif on the C-terminal domain of PCNA are critical for the Rep-PCNA interaction.

**Fig 5:**
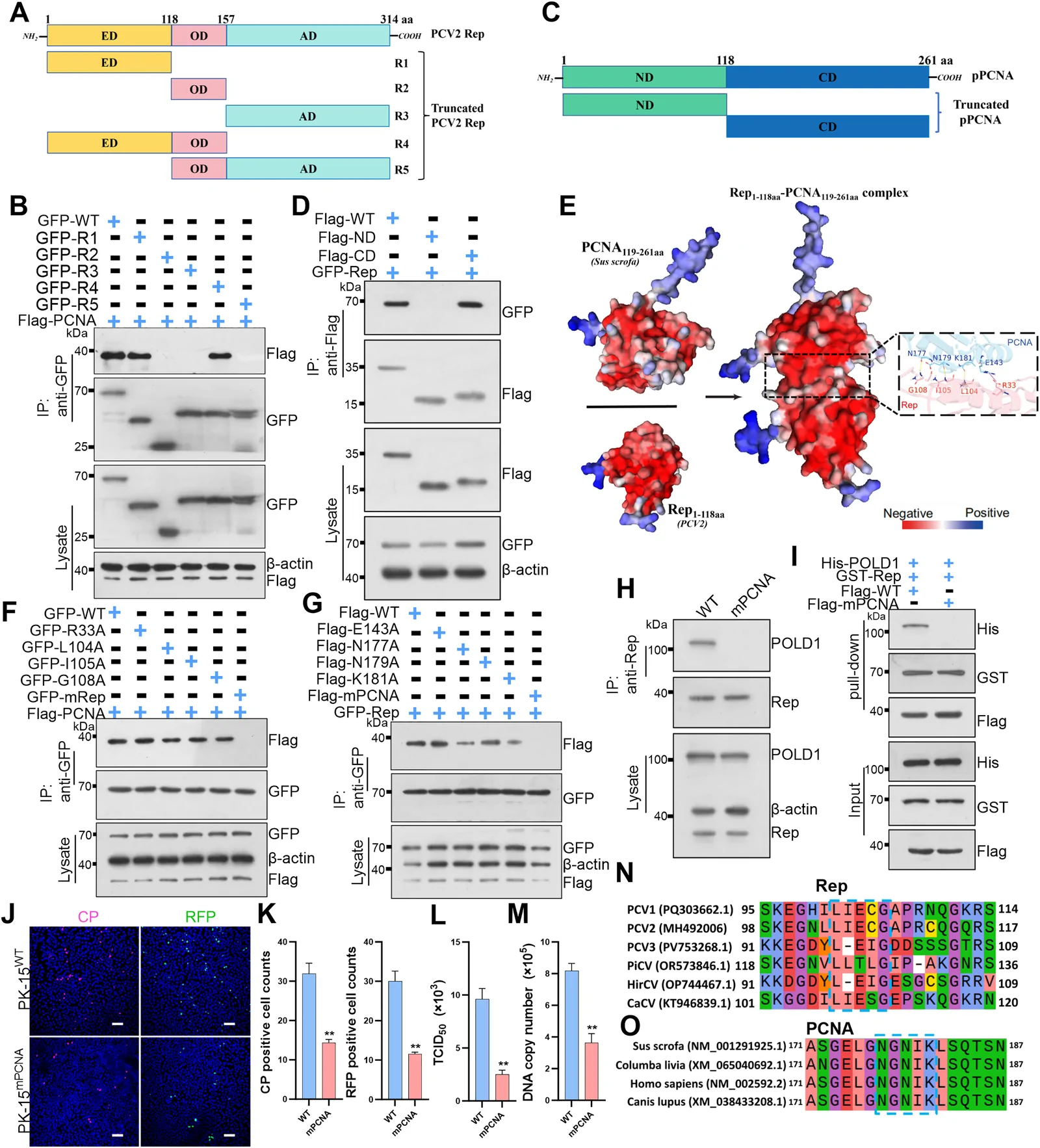
The N-terminal domain of circovirus Rep interacts with the C-terminal domain of PCNA. **(A)** Schematic diagram of the structure of PCV2 Rep; ED domain stands for the endonuclease domain; OD domain stands for the oligomerization domain; AD domain stands for the ATPase domain. (B) co-IP assays to detect the interaction between different truncations of the PCV2 Rep protein and PCNA. **(C)** Schematic diagram of the structure of PCNA. (D) co-IP assays to detect the interaction between different truncations of the PCNA protein and PCV2 Rep. **(E)** Prediction of the interaction sites between PCV2 Rep and PCNA using AlphaFold 3. **(F)** Co-IP assay detecting the interaction between different PCV2 Rep mutants and PCNA; The mRep mutant bears three alanine substitutions at positions 104 (L104A), 105 (I105A), and 108 (G108A), and does not contain the R33A mutation. (G) co-IP assays detecting the interaction between different PCNA mutants and the PCV2 Rep protein; The mPCNA mutant bears three alanine substitutions at positions 177 (N177A), 179 (N179A), and 181 (K181A), and does not contain the E143A mutation. (H) co-IP assays detecting the interaction between PCV2 Rep and POLD1 in PK-15^PCNA-WT^ or PK-15^mPCNA^. **(I)** GST pull-down assay to evaluate the effect of PCNA-WT or mPCNA on the interaction between PCV2 Rep and POLD1. (J, K, L, **M)** The effect of PCNA mutation on PCV2 replication. **(J)** Positive cells were photographed using a fluorescence microscope. Bars = 100 μm are indicated in each panel. **(K)** The counts of CP-positive and RFP-positive cells per 100 cells. **(L)** The PCV2 TCID_50_ of these cells was measured and calculated. **(M)** The DNA copy numbers of PCV2 were detected and calculated by qPCR. **(N)** Sequence alignment of Rep proteins from different circovirus species. **(O)** Sequence alignment of PCNA proteins from different species. **p < 0.01 versus the WT group in **(K)**, (L) and **(M)**.

### R428 inhibits PCV2 replication by disrupting the Rep-PCNA interaction *in vitro*

Based on the identified PCV2 Rep and porcine PCNA motifs as well as their protein structures, we performed a virtual screen using the MTiOpenScreen drug library (https://bioserv.rpbs.univ-paris-diderot.fr/services/MTiOpenScreen/) to identify small molecule compounds that may interfere with the interaction between PCV2 Rep and porcine PCNA. The screening strategy was aimed at identifying candidate molecules that are capable of binding to the identified Rep-PCNA binding interface with high affinity, thereby competitively blocking the Rep-PCNA interaction. After screening a library of 7,173 drugs, we obtained a set of preliminary candidate molecules that may bind to both PCV2 Rep and porcine PCNA (Fig 6A and S2 Table). Seven drugs with binding energies with Rep and PCNA both less than -7.0 kcal/mol were selected for subsequent experiments (Fig 6A). The CC_50_ values of all these drugs on PK-15 cells were measured (Fig 6B and S7A Fig in S1 File). Among them, only R428 suppressed PCV2 DNA replication in a dose-dependent manner at non-cytotoxic concentrations (Fig 6C and S7B Fig in S1 File). TCID_50_ and FISH assays further confirmed that R428 suppresses PCV2 replication (Fig 6D, 6E). To verify whether R428 directly binds to PCNA, we performed molecular docking analysis, and the results showed that R428 directly interacts with the N177, N179, and K181 residues of PCNA (S8A Fig in S1 File). Subsequently, we used DARTS and CETSA to confirm the binding of R428 to porcine PCNA, and the results showed that the treatment of R428 significantly enhanced the stability of PCNA (Fig 6F, 6G). Further, SPR analysis further demonstrated that R428 bound porcine PCNA but not the PCV2 Rep-binding-site mutant of porcine PCNA (Fig 6H, 6I, S8B Fig in S1 File and S3, S4 Tables). co-IP results showed that the treatment of R428 significantly reduced the interaction of Rep and PCNA as well as POLD1 in a dose-dependent manner in PCV2-infected cells (Fig 6J, 6K and S8C, 8D, 8E, 8F Fig in S1 File). Furthermore, to rule out the possibility that R428 suppresses PCV2 replication through its classical AXL inhibition, we performed AXL knockdown experiments using specific shRNA in PK-15 cells. The results showed that AXL knockdown did not significantly affect PCV2 replication, indicating that R428 does not suppress viral replication through its canonical AXL inhibition (S8G, 8H Fig in S1 File). In summary, these findings demonstrate that R428 can inhibit PCV2 replication in PK-15 cells by blocking the interaction between Rep and PCNA, thereby interfering with the recruitment of the host DNA polymerase POLD1 by Rep.

**Fig 6:**
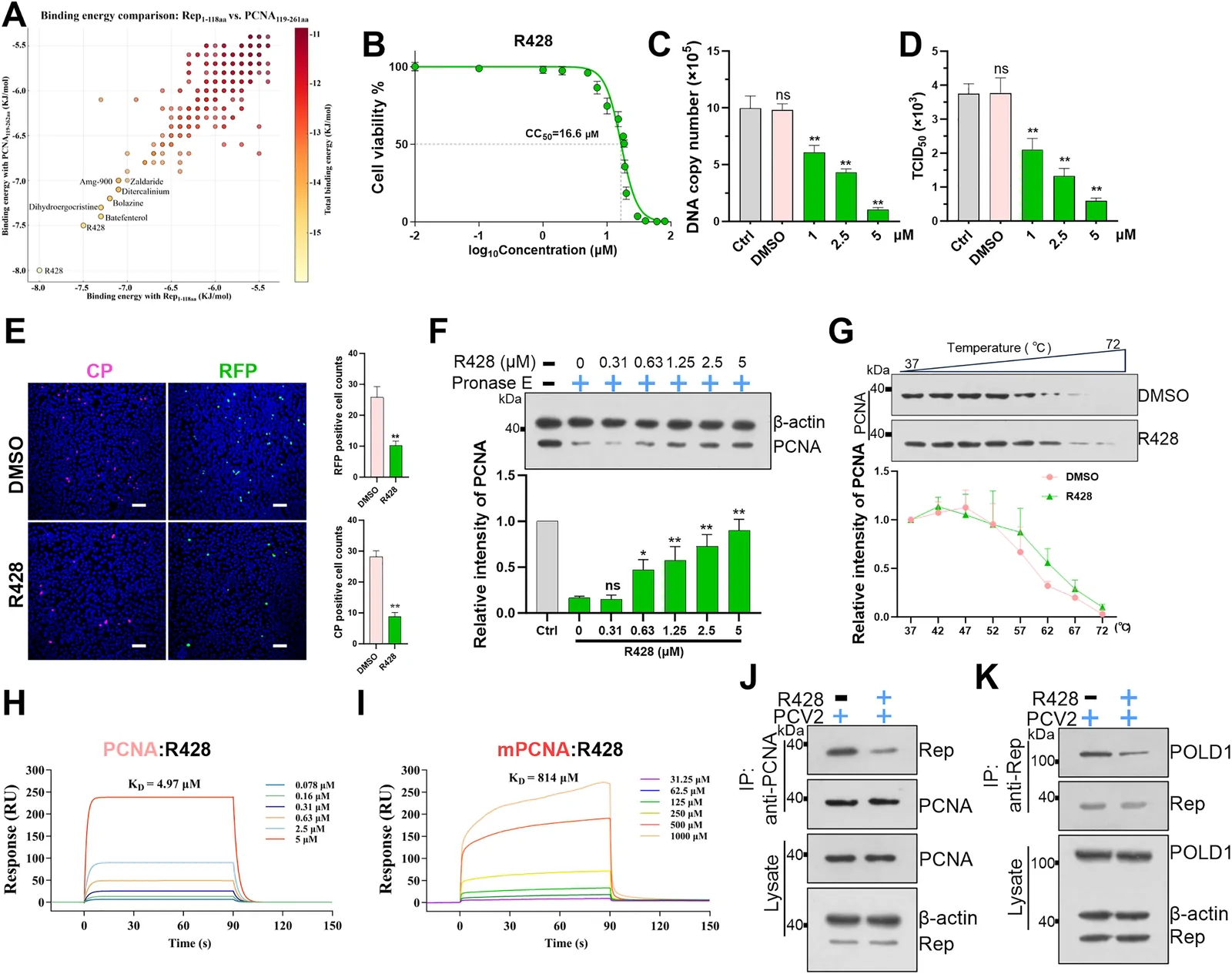
R428 inhibits PCV2 replication by disrupting the Rep-PCNA interaction in vitro. **(A)** Virtual screening of small-molecule compounds targeting the interaction interface between circovirus Rep and PCNA. **(B)** CCK-8 assay to detect the effect of different concentrations of R428 on cell viability. **(C)** The DNA copy numbers of PCV2 were detected and calculated by qPCR. **(D)** The PCV2 TCID_50_ of these cells was measured and calculated. **(E)** Positive cells were photographed using a fluorescence microscope. Bars = 100 μm are indicated in each panel. The counts of CP-positive and RFP-positive cells per 100 cells, **p < 0.01 versus the DMSO group. **(F)** DARTS assay to detect the binding between R428 and PCNA. **(G)** CETSA assay to detect the binding between R428 and PCNA. (H, **I)** SPR assay to detect the binding between R428 and PCNA-WT or mPCNA. (J) co-IP assay to detect the effect of R428 on the Interaction between PCV2 Rep and PCNA. (K) co-IP assay to detect the effect of R428 on the Interaction between PCV2 Rep and POLD1. **p < 0.01 versus the DMSO group in **(C)**, (D) and **(F)**.

### R428 alleviates PCV2 infection-induced pathological changes and reduces viral replication *in vivo*

To systematically evaluate the therapeutic potential of R428 against PCV2 infection in animals, we first conducted experimental validation in a mouse model. Four-week-old mice were infected with PCV2 for 7 days and then administered R428 at two doses (R428-L, 25 mg/kg; R428-H, 75 mg/kg), ribavirin, or PBS daily by oral gavage for the following 14 days. The results showed that both R428 doses significantly alleviated PCV2-induced body-weight loss, with the high dose producing the strongest protective effect. Ribavirin also reduced body-weight loss, but its effect was weaker than that of either R428 dose (Fig 7A). Serum viral-load and TCID_50_ assays showed that R428 and ribavirin significantly suppressed PCV2 replication, with high-dose R428 producing the strongest effect (Fig 7B, 7C). Further histopathological analysis indicated that PCV2 infection induced typical interstitial pneumonia, characterized by thickened alveolar walls, widened interstitial spaces, and inflammatory cell infiltration (Fig 7D, 7E). In the lymph nodes, structural blurring of the white pulp, atrophy of germinal centers, reduced lymphocyte counts, and congestion in the red pulp were observed (Fig 7D, 7E). In contrast, these pathological injuries induced by PCV2 infection in both the lungs and lymph nodes were significantly alleviated by R428 treatment, especially by the high dose of R428 treatment (Fig 7D, 7E). In PCV2-infected piglets, high-dose R428 treatment (8.4 mg/kg) markedly alleviated PCV2-induced wasting, resulting in significantly higher average body weight (Fig 7F). The treatment of R428, especially the high dose of R428, significantly suppressed the replication of PCV2 in piglets (Fig 7G, 7H). Histopathological analysis confirmed that PCV2 infection also induced interstitial pneumonia in the lungs and structural damage in the lymph nodes of piglets, while R428 treatment markedly mitigated these histopathological changes (Fig 7I, 7J). In summary, this study demonstrates that R428 treatment effectively inhibits PCV2 replication in both mouse and piglet models and significantly alleviates PCV2 infection-induced tissue damage, indicating its potential as a candidate anti-PCV2 therapeutic agent.

**Fig 7:**
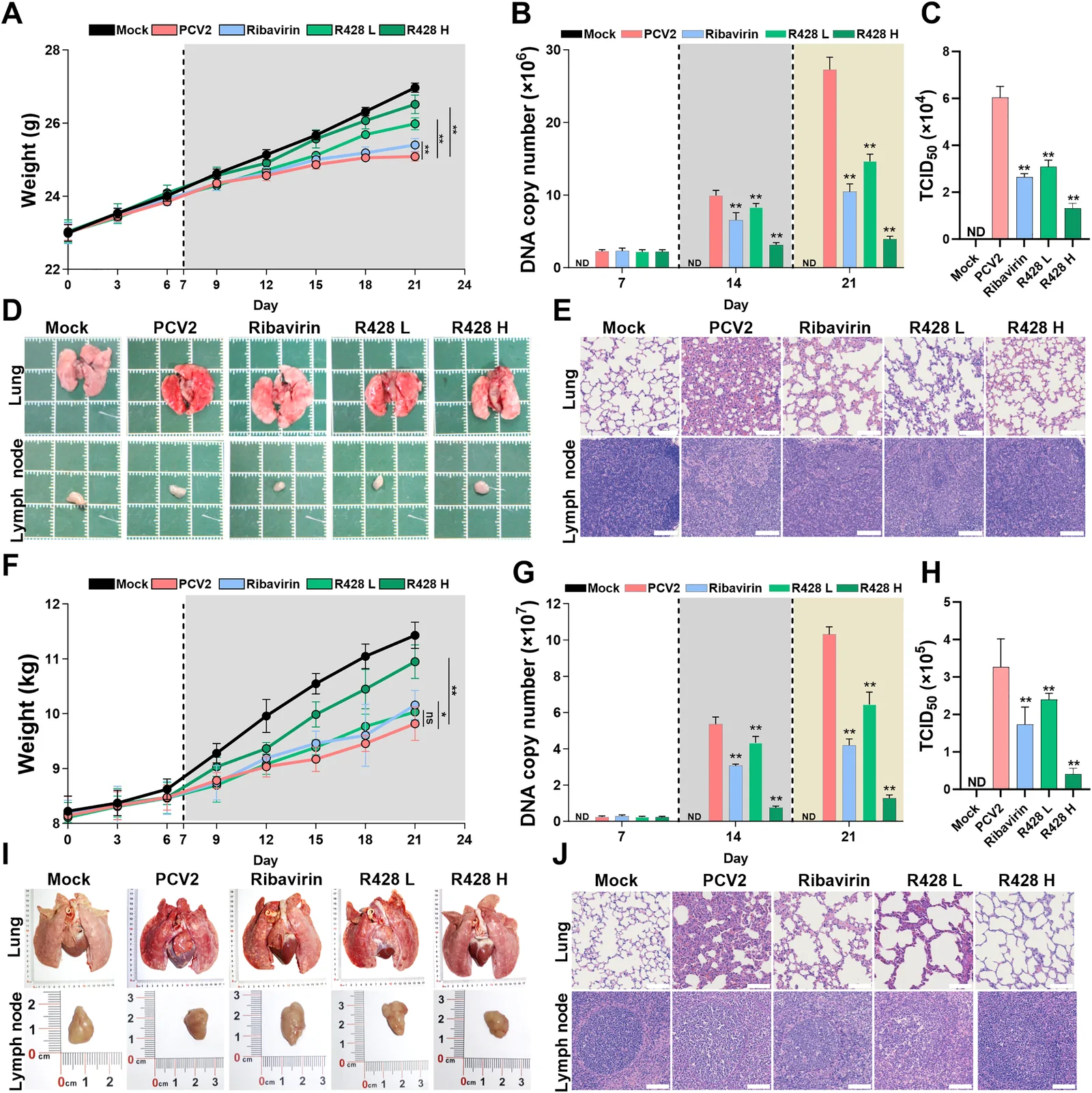
R428 alleviates PCV2-induced pathogenesis and reduces viral replication in vivo. **(A)** Changes in body weight of mice. **(B)** The DNA copy numbers of PCV2 were detected and calculated by qPCR. **(C)** The PCV2 TCID_50_ of these cells was measured and calculated. **(D)** Gross observation of lungs and lymph nodes of mice. **(E)** Pathological sections of lungs and lymph nodes of mice. **(F)** Changes in body weight of piglets. **(G)** The DNA copy numbers of PCV2 were detected and calculated by qPCR. **(H)** The PCV2 TCID_50_ of these cells was measured and calculated. **(I)** Lungs and lymph nodes of piglets in different treatment groups. **(J)** Pathological sections of lungs and lymph nodes of piglets. **p < 0.01 versus the PCV2 group in **(A)**, **(B) (C)**, **(F)**, (G) and **(H)**. R428 L (25 mg/kg) and R428 H (75 mg/kg) in mice. R428 L (2.8 mg/kg) and R428 H (8.4 mg/kg) in piglets.

## Discussion

Circoviruses exhibit a broad host range, including pigs [4], pigeons [31], geese [32], dogs [2], and humans [10,12], and some are associated with numerous immunosuppressive diseases of their hosts [11,33]. Porcine circovirus type 2 (PCV2) is the most extensively studied representative member, having been found to lead to immunosuppression in piglets and cause significant economic losses to the pig farming industry for decades [34]. Currently, widespread vaccine use has reduced PCV2-associated losses. However, these vaccines do not eliminate PCV2 from infected pigs or fully prevent viral spread within herds [35]. More importantly, there are still no available commercial vaccines for other circoviruses, such as PCV3, PCV4, BFDV, and PiCV, which have also been associate with a range of diseases [36]. In this present study, we found that circoviruses, including PCV2, PiCV, HirCV, and CaCV, hijack host DNA polymerase POLD1 through the interaction of their replication-associated protein Rep and host PCNA to facilitate their genomic DNA replication, and R428 efficiently blocked the Rep-PCNA interaction to suppress PCV2 replication and alleviate PCV2 infection-induced pathological changes in animals. These results highlight the potential utility of R428 as a candidate anti-circovirus therapeutic agent in the future.

In the past, there have been studies showing that circoviruses have a single-stranded circular DNA genome with a stem-loop structure, and it undergoes rolling-circle replication. The replication-associated protein Rep is a crucial non-structural protein for circovirus replication, and truncation or mutations in Rep of PCV2 lead to a significant reduction in viral protein synthesis and completely abrogate DNA replication [37,38]. However, Rep has only site-specific nickase and ligase activities and lacks canonical helicase and DNA polymerase activities. Consequently, the elongation and strand displacement of the viral genome primarily rely on the host replication machinery. More than 15 distinct DNA polymerases have been identified in host cells [39]. Using DNA pull-down, we found that POLD1, the core catalytic subunit of DNA polymerase δ (Pol δ), was employed by circoviruses to replicate their genomic DNA. Since Rep is the major protein encoded by circoviruses to regulate viral DNA replication, we hypothesized that POLD1 might interact with Rep. However, our further results showed that POLD1 did not directly bind to Rep, instead, the interaction was mediated by PCNA, which has been found to be crucial for regulating POLD1 enzymatic activity [40]. Similarly, there are studies that show that the PCNA-POLD1 complex can be hijacked by AAV (a small linear single-stranded DNA virus) and HBV (a double-stranded circle DNA virus) to replicate the viral DNA [29,41,42], indicating that the PCNA-POLD1 complex may be a prime target for small DNA viruses seeking to replicate their own genomes. In this study, we found that the Rep of porcine, human, pigeon, and canine circoviruses all directly bind to their host PCNA through a pair of highly conserved motifs (LIXXG on Rep and NXNXK on PCNA). The high conservation of these interaction sites suggests that different circoviruses use essentially the same DNA replication mechanism, which lays the structural foundation for developing broad-spectrum circovirus control strategies.

Currently, no specific commercially available antiviral drugs exist for treating circovirus infections. The clinically used drugs for circoviruses (mainly PCV2 and Pigeon circovirus, PiCV) associated diseases primarily focus on symptomatic treatment and preventing secondary infections, commonly including antibiotics and supportive care medications. While vaccines for PCV2 have been widely adopted and have been effective in reducing the severity of disease, their efficacy can be challenged by the continuous emergence of new viral genotypes and the high mutation rate inherent to single-stranded DNA viruses. The threat of vaccine-breakthrough infections, coupled with the lack of therapeutic options for other pathogenic circoviruses affecting a wide range of species, creates a compelling need for the development of novel, direct-acting antiviral agents. In recent years, aided by advances in computer-aided drug design and structural biology, particularly the application of molecular docking technologies, a series of antiviral drugs have been developed. Molecular docking and high-throughput virtual screening have emerged as powerful methodologies for identifying compounds that disrupt virus-host interactions, with applications ranging from SARS-CoV-2 main protease inhibitors to multi-target anticancer drug design [43–46]. Similarly, a combination of computer-aided drug design and biophysical assays successfully identified a promising inhibitor of porcine reproductive and respiratory syndrome virus (PRRSV), providing a lead candidate for the major swine pathogen [47].

Through structure-based virtual screening, we identified R428 as a potent inhibitor of the Rep-PCNA interaction. Both Drug Affinity Responsive Target Stability (DARTS) and Cellular Thermal Shift Assay (CETSA) are used to evaluate whether a compound binds to its target protein and enhances its stability. Specifically, DARTS assesses binding by measuring the protection of the target protein from protease digestion, whereas CETSA evaluates binding by monitoring changes in the thermal denaturation profile of the protein. Our results demonstrated that R428 can directly bind to PCNA, and further surface plasmon resonance (SPR) experiments showed that R428 specifically binds to wild-type PCNA but not to the mutant PCNA. Mechanistically, we demonstrated that R428 binds PCNA and disrupts its interaction with Rep, thereby preventing POLD1 recruitment and halting viral DNA synthesis. A classical rescue experiment to further validate the specificity of R428 is not feasible in our system, because R428 directly targets the Rep-interaction interface on PCNA; any mutation that abolishes R428 binding would inevitably disrupt Rep interaction and thus cannot support PCV2 replication, rendering a rescue experiment uninterpretable. We provide multiple lines of direct evidence: R428 inhibits the Rep–PCNA interaction in a dose-dependent manner, disrupts Rep–POLD1 recruitment, and molecular docking analysis shows that R428 occupies the Rep-binding surface on PCNA. Together with the antiviral activity data, these findings support the conclusion that R428 targets the Rep–PCNA interface. It should be noted, however, that R428 was originally developed as an anti-tumor agent targeting AXL receptor tyrosine kinase [48–50], and at the low micromolar concentrations used in this study, it may exert additional off-target or broad-spectrum kinase inhibitory effects. Our AXL knockdown experiments have excluded the canonical AXL pathway as the primary mediator of the observed antiviral activity, yet the possibility of other off-target contributions cannot be completely ruled out. While comprehensive kinome-wide profiling is beyond the scope of the current study, our cumulative evidence—spanning biophysical binding assays, mutational analysis, and functional antiviral readouts—strongly supports a specific, on-target mechanism via the Rep–PCNA interface. Nevertheless, the precise molecular targets and potential polypharmacology of R428 in the context of PCV2 infection warrant further chemoproteomic and structural investigations. In both cell culture and animal models, including mice and piglets, R428 treatment significantly reduced viral load and ameliorated PCV2-associated tissue pathology. R428 has recently been implicated in antiviral activity against RSV [51], Zika Virus [52], and SARS-CoV-2 [53]. As an investigational drug currently being evaluated in clinical trials, R428 may have potential for future clinical applications [54]. However, R428 may not be an ideal drug for animals, as its current formulation and cost profile necessitate further optimization. Structure-activity relationship studies aimed at improving potency, reducing toxicity, and lowering production costs will be critical for large-scale agricultural deployment. Future antiviral development may also benefit from emerging technologies, including deep learning-driven virtual screening, multi-scale modeling strategies, and automated closed-loop screening platforms integrating artificial intelligence with experimental validation. Our team is actively pursuing analog synthesis and screening to identify more cost-effective derivatives suitable for veterinary applications. Such advances would have significant implications for animal health and the economic sustainability of livestock production, particularly given the global prevalence of PCV2 and its substantial economic impact on the swine industry. More importantly, the continuous discovery of novel circoviruses in both animals and humans, especially the circoviruses found in organ transplant patients [11,55], further underscores the broader relevance of this work for human health.

In summary, this work fundamentally redefines the molecular basis of circovirus DNA replication by identifying a conserved host-factor hijacking mechanism centered on the Rep–PCNA–POLD1 axis. The *in vivo* efficacy of R428 provides compelling proof-of-concept for host-targeted therapeutics against circoviruses. By bridging the fundamental gap in understanding circovirus DNA replication while addressing the urgent need for new antiviral strategies, this study provides both a critical theoretical foundation and a promising new avenue for controlling diseases caused by the circovirus family.

## Materials and methods

### Ethics approval and consent to participate

All animal experiments were approved by the Institutional Animal Care and Use Committee of Northwest A&F University, China (permit number: IACUC 2025–1104) and were performed according to the Animal Ethics Procedures and Guidelines of the People’s Republic of China.

### Cell lines

All cell lines used in this study tested negative for mycoplasma contamination. PK-15 and HEK293T cells were maintained in our laboratory and cultured in complete DMEM medium. These cells were maintained at 37°C in a humidified incubator with 5% CO_2_.

### Viruses

PCV2 (GenBank: MH492006) was stocked in our laboratory and propagated in PK-15 cells. The 50% tissue culture infective dose (TCID_50_) of PCV2 was measured as follows: the viral preparation was serially diluted and then incubated with PK-15 cells. Post 48 h incubation, the PCV2-infected cells were detected by indirect immunofluorescence assay with a PCV2 Cap antibody, and the TCID_50_ was calculated according to the Reed–Muench method.

### shRNA

The shRNA oligonucleotides targeting the indicated genes were designed using the RNAi designer tool from OligoEngine (http://www.oligoengine.com) and synthesized by Tsingke Biotechnology Co., Ltd. (Beijing, China). Each pair of forward and reverse oligonucleotides was annealed in annealing buffer (10 mM Tris-HCl pH 8.0, 50 mM NaCl, 1 mM EDTA) at a final concentration of 50 µM by heating at 95°C for 5 min, followed by gradual cooling to room temperature over approximately 1 h. The annealed double-stranded shDNA fragments were then ligated into the *Bam*H I and *Eco*R I restriction sites of the pSIH1-H1-copGFP-T2A-Puro vector (System Biosciences). The resulting shRNA constructs were verified by Sanger DNA sequencing (Tsingke Biotechnology Co., Ltd.). The specific primer sequences are listed in S5 Table.

### Cytotoxicity assays

PK-15 cells were maintained under standard conditions and seeded into 96-well plates at 1 × 10^5^ cells per well. Cells were cultured until approximately 80% confluence and then exposed to serial two-fold dilutions of the test compound prepared in DMEM. A total of 100 μL of each concentration was added per well, with six replicate wells per condition. Negative control wells were included. Cells were incubated at 37°C in 5% CO_2_ for 48 h. Cell viability was assessed using a CCK-8 assay (Solarbio, CA1210). After treatment, culture medium was replaced with 100 μL DMEM containing 10 μL CCK-8 reagent per well, followed by incubation for 2 h at 37°C. Absorbance was measured at 450 nm using a microplate reader. Cell viability was calculated using the following formula: Cell viability (%) = [(OD_treatment − OD_blank)/ (OD_control − OD_blank)] × 100%.

### Western blot

Protein samples were separated by SDS-PAGE and subsequently transferred onto PVDF membranes. The membranes were blocked with 5% skimmed milk in PBS for 2 h at room temperature, followed by incubation with primary antibodies overnight at 4°C. After washing with PBS containing 0.1% Tween 20 (PBST), the membranes were incubated with horseradish peroxidase (HRP)-conjugated secondary antibodies for 2 h at room temperature. Following another series of washes with PBST, the blots were developed using an enhanced chemiluminescence (ECL) substrate and exposed to X-ray film in a darkroom. Band intensities were quantified using ImageJ software. The following primary antibodies used for Western blot were all used at a dilution of 1:1000 (unless otherwise indicated): Selleck Anti-POLD1 (host: Rat) Catalog #: F2835; Selleck Anti-PCNA (host: Rat) Catalog #: F0018; Selleck Anti-β-actin (host: Rat) Catalog #: F0012; Selleck Anti-GFP (host: Rat) Catalog #: F0055; Selleck Anti-Flag (host: Rat) Catalog #: F3506; HRP-conjugated secondary antibody (Abmart, M21009); The anti-PCV2 Rep polyclonal antibody was generated in our laboratory by immunizing rabbit with purified prokaryotically expressed PCV2 Rep protein.

### DNA pull-down and immunoprecipitation

The Biotin-labeled primers (GAACCGCGGGCTGGC(dT-Bio)GAACTC and GCACCGCGGAAATTTCTGACAAACGTTACA) were used for PCR amplification of the full length of PCV2 DNA. The PCR products were cyclized and transfected into PK-15 cells for 12 h. The cells were lysed on ice, and the PCV2 DNA-interacting proteins were pulled down using streptavidin-conjugated beads. The pulled proteins were then mixed with loading buffer, denatured by boiling, and separated by SDS-PAGE. The target gel bands were excised, followed by in-gel digestion. The resulting peptides were then analyzed by Liquid Chromatography-Mass Spectrometer/Mass Spectrometry (LC-MS/MS).

### Quantitative PCR (qPCR)

Total DNA was extracted from cells with phenol-chloroform and treated with RNase to eliminate potential viral RNA contamination. Quantification of copies of the circovirus genome was performed as previously described [56]. After infecting cells with PCV2 for different time periods, the cells were harvested, total RNA was extracted, and analysed by RT-qPCR. The primer sequences were as follows: qPCV2-F: 5’-ATAACCCAGCCCCTCTCTAC-3’; qPCV2-R: 5’-GGCCTACGTGTGTTCTAC-ATTTCC-3’; qPOLD1-F5’-CAACTCTGTGTACG-GCTTCACT-3’; qPOLD1-R5’-TGCTTTGTCTTCTCGATCATCT-3’; qGAPDH-F: 5’-ACATCATCCCTGCTTCTA-CTGG-3’; qGAPDH-R: 5’-CTCGGACGCCTGCTTCAC-3’.

### TCID_50_

PK-15 cells were seeded into 96-well plates at a density of 1–2 × 10⁴ cells per well and cultured overnight at 37 °C in 5% CO_2_ to reach 70–80% confluence. The PCV2 stock was serially diluted 10-fold in maintenance medium (DMEM supplemented with 2% foetal bovine serum), and 100 μL of each dilution was added to eight replicate wells; control wells received maintenance medium only. After virus adsorption at 37 °C for 1 h, the inoculum was removed and replaced with fresh maintenance medium, and the cells were incubated for a further 48–72 h. Subsequently, the medium was discarded, and the cells were fixed with ice-cold 80% acetone for 15 min, followed by blocking with 5% bovine serum albumin in PBS for 30 min at room temperature. The cells were then incubated with a PCV2-specific primary antibody (diluted 1:200 in blocking buffer) overnight at 4 °C, washed three times with PBST, and incubated with a fluorescein-conjugated secondary antibody (1:500) for 1 h at 37 °C in the dark. After thorough washing, the cells were examined under a fluorescence microscope, and wells displaying distinct green fluorescence were scored as positive. The number of positive wells at each dilution was recorded, and the viral titre (TCID₅₀ per mL) was calculated according to the Reed–Muench method.

### Fluorescence in situ hybridization (FISH)

Two probes targeted for PCV2 DNA genomes (CP) or DNA replication form (RFP) were designed and synthesized. The probe for CP was labeled by CY3 (Red), and the sequence was 5’-CY3-TTTGATTATTTTTGTGGCGAGGAGGTAGGTAGGA-GGATAGAGGAGGAGGAG-3’. The probe for RFP was labeled by FAM (Green), and the sequence was 5’-FAM-CCTTCCTTCCTCTCCCTCGCCAATAAAATAATC-AAA-3’. The detailed procedure for detecting PCV2 replication by FISH was performed as previously described.

The PK-15 cells were seeded on round coverslips. The cells were infected by 1 MOI PCV2 for 24 h and then washed twice. The cells on the coverslips were fixed with ethanol for 30 min and permeabilized with 0.1% Triton X-100 for 20 min. After three washes, the cells were incubated with 2 × SSC at 37°C for 30 min. Cells were then dehydrated with 75%, 90%, and 100% ethanol, respectively. The freshly prepared hybridization buffer containing the probes was used to incubate the cells in the dark at 37°C overnight. The hybridization buffer was replaced by 0.4 × SSC containing 0.3% Tween20, preheated at 65°C, and the cells were incubated at 37°C for 2 min. The cells were subsequently incubated with preheated 2 × SSC containing 0.1% Tween20 at 37°C for 2 min. Finally, the cells were stained with DAPI at 37°C for 15 min. After three washes, the cells were prepared for imaging.

### Chromatin immunoprecipitation (ChIP)

PK-15 cells were infected with 1 MOI PCV2 or mock for 12 h. Alternatively, HEK293T cells were transfected with a Flag-tagged POLD1 plasmid for 12 h, followed by a second transfection with in vitro-circularized porcine circovirus DNA for an additional 12 h. Cells were cross-linked with formaldehyde and lysed with the nuclei lysis buffer to release chromatin. The chromatin lysate was sonicated, then quantified and divided into 100 μg DNA portions. The DNA portions were precleared by protein A (G) - agarose/salmon sperm DNA beads and then incubated with the POLD1 (or Flag) monoclonal antibodies and irrelevant antibodies at 4°C overnight. Protein A (G) - agarose/salmon sperm DNA beads were added to the samples again to bind to the antibody-bound immune complexes. Then the compounds were digested by proteinase K, and extracted with phenol: chloroform to purify nucleic acids. The nucleic acids were resuspended in H_2_O and analyzed by PCR. The primer sequences were as follows: PCV2-Forward: 5’-GATGATCTACTGAGACTGTGT-3’; PCV2-Reverse: 5’- ATA-GAGAGCTTCTACAGCTG-3’; PiCV-Forward: 5’-TGACTTCCACTACGCCATA-3’; PiCV-Reverse: 5’-TTCTCATTGTCCTCGTCAC-3’; HiCV-Forward: 5’-AGGCTT-GTCCTTCGGTAT-3’; HiCV-Reverse: 5’-CACAATCCTCCATAAC-ATATTTAG-3’; CaCV-Forward: 5’-AAGTGGAAGACGGAAGTG-3’; CaCV-Reverse: 5’-GAGGG-TACCGGTCCAAAAG-3’; β-actin-Forward: 5’-GGACT-TCGAGCAGGAGATGG-3’; β-actin-Reverse: 5’-TTCCGCTGTGGCCACCAC-3’.

### Confocal microscopy assay

The PK-15 cells were seeded on round coverslips in 24-well plates overnight. After PCV2 infection for 24 h, the cells were fixed with 4% paraformaldehyde for 30 min and permeabilized with 0.1% Triton X-100 for 20 min. After blocking with 2% BSA, the cells were incubated with primary antibodies and then with the corresponding fluorescent secondary antibodies, followed by staining with 4,6-diamidino-2-phenylindole (DAPI) for 10 min and finally examined with confocal microscopes.

### Co-immunoprecipitation (co-IP) assay

The 293T cells were seeded in 10 cm cell culture dishes, and then co-transfected with plasmids for 24 h. The cells were harvested and lysed in an ice bath for 30 min, then centrifugated at 12,000 rpm for 10 min, and the supernatants were collected. The obtained supernatants were incubated with GFP-Nanoab-Magnetic Beads (LABLEAD, GNM-25–1000) or Flag-Nanoab-Magnetic Beads (LABLEAD, FNM-25–1000) at 4°C for 6 h, then the beads were washed five times, and the proteins were eluted for Western blot.

### Expression and purification of GST-tagged proteins and his-tagged proteins

The pGEX-6P-1 empty vector, GST-tagged Rep constructs, and pET-28a plasmids encoding His-tagged proteins were transformed into *E. coli* Rosetta (DE3) competent cells. Recombinant protein expression was induced under optimized conditions. Cells were harvested and resuspended in lysis buffer containing 20 mmol/L Tris-HCl, 150 mmol/L NaCl, 10% glycerol, PMSF, and protease inhibitors. Following sonication and centrifugation, the soluble fractions were collected for protein purification. GST-tagged proteins were purified using glutathione-Sepharose beads, while His-tagged proteins were purified using Ni-NTA agarose beads according to standard affinity purification procedures. Purified proteins were washed and eluted, followed by dialysis against PBS or buffer A and storage at −80 °C until further analysis. Protein expression and purification were confirmed by SDS-PAGE and western blotting using anti-GST or anti-His antibodies.

### GST pull-down assay

Equal amounts of GST or GST-tagged proteins immobilized on glutathione-Sepharose beads were incubated with purified His-tagged proteins in buffer A at 4 °C for 3 h. After incubation, the beads were washed three times with buffer A. Bound proteins were eluted by boiling in protein loading buffer and analyzed by SDS-PAGE and western blotting using anti-GST and anti-His antibodies.

### Drug affinity responsive target stability (DARTS) analysis

PK-15 cells were seeded evenly in cell culture dishes at an appropriate density. Upon reaching 70–80% confluence, the cells were infected with PCV2 at an MOI of 1 for 24 h. After infection, cells were collected, washed with PBS, and lysed using RIPA lysis buffer (without protease inhibitors) for 30 min. The lysates were centrifuged at 12,000 × g and 4 °C for 15 min, and the supernatant was collected. Protein concentration was determined by BCA assay and adjusted to 2 μg/μL. Aliquots of 100 μL were dispensed into seven tubes labeled A, B, C, D, E, F, and G. Tubes A, B, C, D, and E were supplemented with R428 stock solution (0.31 μM, 0.63 μM, 1.25 μM, 2.5 μM, and 5 μM, final DMSO concentration 0.5%), while tubes F and G received an equivalent volume of DMSO. All samples were gently agitated at room temperature for 1 h. Subsequently, 2 μL of pronase stock solution (0.5 mg/mL) was added to tubes A-F, followed by gentle shaking at room temperature for 5 min. The reaction was terminated immediately by adding 5 μL of 100 × protease inhibitor cocktail and placing the samples on ice. An equal volume of 20% trichloroacetic acid (TCA) was then added, and the samples were incubated at 4 °C for 30 min. After centrifugation at 12,000 g and 4 °C for 15 min, the pellets were washed once with cold acetone and air-dried. Pellets were resuspended in 30 μL of 1 × SDS loading buffer and denatured in a boiling water bath for 10 min. Finally, samples were analyzed by Western blot.

### Cellular thermal shift assay (CETSA)

PK-15 cells were randomly divided into two groups: the experimental group was treated with 5 μM R428 for 4 hours, while the control group was treated with an equal volume of DMSO for the same duration. After treatment, cells were digested with trypsin, collected, and washed three times with PBS. The cells were then resuspended in lysis buffer and disrupted by sonication to prepare cell lysates. The lysates were equally aliquoted into eight PCR tubes and subjected to a temperature gradient (37°C, 42°C, 47°C, 52°C, 57°C, 62°C, 67°C, 72°C) for 3 minutes of heating. After heating, the samples were centrifuged at high speed, and the supernatant was carefully transferred to new tubes. The protein concentration of each supernatant was determined using the BCA assay, and target proteins were finally detected by Western blot.

### Surface plasmon resonance (SPR) assay

Binding affinity and kinetics evaluation of small molecule compounds with target proteins were performed using a Biacore 1K system (Cytiva). Recombinant PCNA and its mutant form (mPCNA) were immobilized onto a Series S Sensor Chip CM5 (BR-1005–30, Cytiva) via amine-coupling chemistry under standard conditions (20 μg/mL in 10 mM sodium acetate buffer, pH 4.5) at a continuous flow rate of 10 μL/min for 10 minutes at 25°C. Analyte R428 was serially diluted in running buffer (1 × PBS-P + , Cytiva) containing 5% (v/v) DMSO. A solvent correction procedure was performed by injecting running buffers with DMSO gradients ranging from 4.5% to 5.8% (v/v) to account for bulk refractive index effects. For each binding cycle, the association phase was monitored for 90 seconds, followed by a 300-second dissociation phase in running buffer. All experiments were conducted in triplicate. Sensorgram data were processed and analyzed using the Biacore 1K Evaluation Software (version 3.0). Binding kinetics and affinity constants were derived by globally fitting the data to a 1:1 Langmuir binding model.

### Mutant cell line construction

A specific pegRNA targeting the designated region of the PCNA gene (Gene ID: 692192, genomic coordinates: NC_010459.5 (14137746..14142584, complement)) was designed using the PRIDICT platform (https://www.pridict.it/), utilizing the PRIDICT2.0 and ePRIDICT algorithms for systematic pegRNA design as described [57]. The designed pegRNA encodes the desired 6-nucleotide substitutions within its reverse transcription (RT) template. The synthesized double-stranded oligonucleotides for the pegRNA were cloned into the pU6-pegRNA-GG-acceptor vector using the Golden Gate assembly method. Correspondingly, the double-stranded sgRNA oligonucleotides were ligated into the MLM3636 vector using T4 DNA ligase. All ligation products were verified by sequencing and used for subsequent experiments. For transfection, a mixture of 750 ng of the PE2-encoding plasmid and 250 ng of the pegRNA-encoding plasmid was prepared. This plasmid mixture was transfected into 3 × 10⁶ PK-15 target cells using Lipo6000 transfection reagent (Beyotime Biotechnology). Post-transfection, cells were seeded into 24-well plates and cultured in medium without nutlin-3a for 7 days to allow recovery and expansion. Subsequently, nutlin-3a was added to the culture medium at a final concentration of 20 µM for positive selection. After continuous selection for 20 days, a stably edited polyclonal cell population was obtained. This cell population was then transferred to T25 flasks for further expansion, preparing it for subsequent monoclonal cell isolation and genotypic characterization.

The primer sequences were as follows:

PegRNA construct mPCNA spacer-F: CACCGCAAGTGGAGAACTCGGAA-AGTTTC; mPCNA spacer-R: CTCTGAAACTTTCCGAGTTCTCCACTTGC; mPCNA extension-F: GTGCTCGTTTGTGACAACGCAATAGCTCCAGCTCCGA-GTTCTCCACTT; mPCNA extension-R: AAAAAGTGGAGAACTCGGAGCTGGA-GCTATTGCGTTGTCACAAACGA. pegRNA scaffold _Optimized: GTTTCAGA-GCTATGCTGGAAACAGCATAGCAAGTTGAAATAAGGCTAGTCCGTTATCAACTTGAAAAAGTGGCACCGAGTCGGTGC; pegRNA: GCAAGTGGAGAACTCG-GAAAGTTTCAGAGCTATGCTGGAAACAGCATAGCAAGTTGAAATAAGGCTAGTCCGTTATCAACTTGAAAAAGTGGCACCGAGTCGGTGCTCGTTTGTGACAACGCAATAGCTCCAGCTCCGAGTTCTCCACTT. Primers for genomic PCR: PCNA199-F: 5’- TGCCGAGATCTCAGTCACAT -3’; PCNA199-R: 5’- AGTTCA-GGTACCTCAGTGCA -3’

### Virtual screening

To identify potential inhibitors that disrupt the Rep–PCNA interaction, an *in silico* screening of a ligand library was conducted using the MTiOpenScreen web platform. This platform is specifically designed for structure-based virtual screening of large compound libraries. Receptor grids were constructed centered on the predicted Rep–PCNA interaction site, as determined by AlphaFold-Multimer. The key residues defined for the interaction site were L104, I105, G108 on Rep, and N177, N179, K181 on PCNA. Screening parameters were optimized to ensure both high throughput and reliable binding predictions, including flexible ligand docking under rigid receptor constraints to preserve protein structural integrity. The docking algorithm employed an empirical scoring function that evaluated binding conformations based on van der Waals forces, hydrogen-bonding potential, and hydrophobic contacts. After screening, compounds were ranked according to their docking scores, and those predicted to bind both Rep and PCNA were selected for further experimental validation.

### Animal experiments and histopathology

Male BALB/c mice (18 ± 2 g) were randomly divided into five groups (n = 6 per group): Mock group, untreated PCV2-infected group, PCV2 + R428 L (25 mg/kg) group, PCV2 + R428 H (75 mg/kg) group, and PCV2 + Ribavirin group. All groups except Mock were intraperitoneally injected with 0.5 mL of PCV2 (10^5.5^ TCID_50_/mL) suspension to establish the infection model. Seven days after infection, mice were administered low-dose R428 (25 mg/kg), high-dose R428 (75 mg/kg), ribavirin, or PBS once daily by oral gavage for 14 consecutive days, while the untreated PCV2-infected group was fed a standard diet. During the treatment, serum samples were collected on days 7 and 14 post-PCV2 infection to determine the PCV2 DNA copy number in the serum. At the end of the treatment period, mice were deeply anesthetized with 5% isoflurane with oxygen (1 L/min) until the toe retraction reflex disappeared. Terminal blood (0.8–1.0 mL) was collected via cardiac puncture using a 1 mL syringe, followed by thoracotomy to ensure exsanguination. Lung tissues were then harvested. Secondary confirmation of death was performed by cervical dislocation, and the absence of respiration and heartbeat was monitored for at least 5 minutes prior to carcass disposal. Serum was separated from terminal blood for the quantification of PCV2 DNA copies, TCID₅₀. A portion of the isolated mouse lung and lymph nodes tissue was used for the detection of PCV2 DNA copies and TCID₅₀, while the remainder was fixed in 4% paraformaldehyde for 24 h, embedded in paraffin, and subsequently processed for histological staining and analysis.

Male piglets (4 weeks) were randomly divided into five groups (n = 3 per group): Mock group, untreated PCV2-infected group, PCV2 + R428 L (2.8 mg/kg) group, PCV2 + R428 H (8.4 mg/kg) group, and PCV2 + Ribavirin group. All groups except Mock were intraperitoneally injected with 0.5 mL of PCV2 (10^5.5^ TCID_50_/mL) suspension to establish the infection model. Seven days after infection, piglets were administered R428 L, R428 H, or PBS once daily by oral gavage for 14 consecutive days, while the untreated PCV2-infected group was fed a standard diet. During the treatment, serum samples were collected on days 7 and 14 post-PCV2 infection to determine the PCV2 DNA copy number in the serum. At the end of the treatment period, piglets were gently restrained, and a venous access was established via the auricular vein. A sodium pentobarbital solution (150 mg/kg body weight) was administered intravenously by slow injection until cardiac and respiratory arrest occurred. Death was confirmed by the absence of corneal reflex, pupillary dilation, and auscultation of heartbeat for more than 2 minutes. Serum was separated from terminal blood for the quantification of PCV2 DNA copies, TCID₅₀. A portion of the isolated piglet lung and lymph-node tissues was used for the detection of PCV2 DNA copies and TCID₅₀, while the remainder was fixed in 4% paraformaldehyde for 24 h, embedded in paraffin, and subsequently processed for histological staining and analysis.

### Statistical analysis

The data are presented as the mean ± SD. Statistical analysis was performed in Prism 8 (GraphPad) software. Comparisons between two groups were performed by an unpaired Student’s t-test, whereas data from multiple groups were analyzed by ANOVA, followed by the Bonferroni post hoc test. Statistically significant and highly significant results were defined as *p* < 0.05 and *p* < 0.01.

## Supporting information

S1 File All Supplementary Figures in the manuscript. (DOCX)

S2 File Rawgel. All original uncropped and unadjusted images of the blot or gel results reported in the manuscript.
(PDF)

S3 File Source Data. All tabular data, original FISH and IFA images, molecular docking and sequence alignment images, as well as gross images and histopathological sections of the lungs and lymph nodes from mice and piglets, are included in the manuscript.
(XLSX)

S1 Table LC-MS/MS identification results of seven DNA polymerases. (XLS)

S2 Table Virtual screening results for small-molecule compounds that target the binding site between PCNA and PCV2 Rep. (XLSX)

S3 Table SPR binding analysis of wild-type PCNA with different concentrations of R428. (XLS)

S4 Table SPR binding analysis of mutant PCNA (mPCNA) with different concentrations of R428. (XLS)

S5 Table All shRNA sequence in the manuscript. (DOCX)
