# Supplementary material for: Hijacking of host PCNA by circovirus replication-associated protein to recruit POLD1 drives viral DNA replication and is inhibited by R428
Source: PLoS Pathog. 2026 Sep 28;22(9):e1014575. doi: 10.1371/journal.ppat.1014575 (PMC13618890; doi:10.1371/journal.ppat.1014575)

Figure 1C

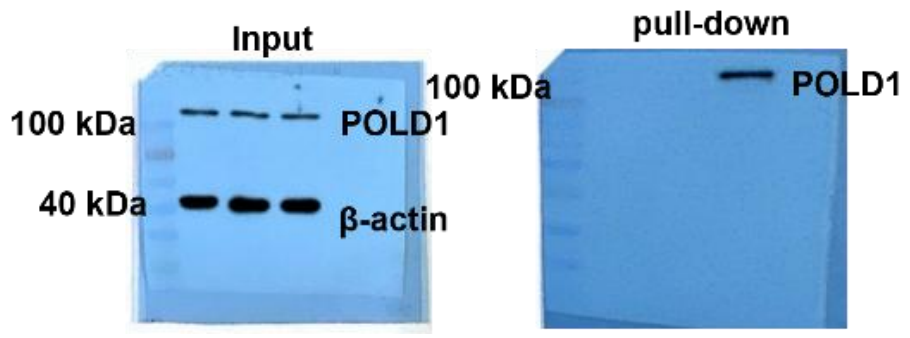

Figure 1D

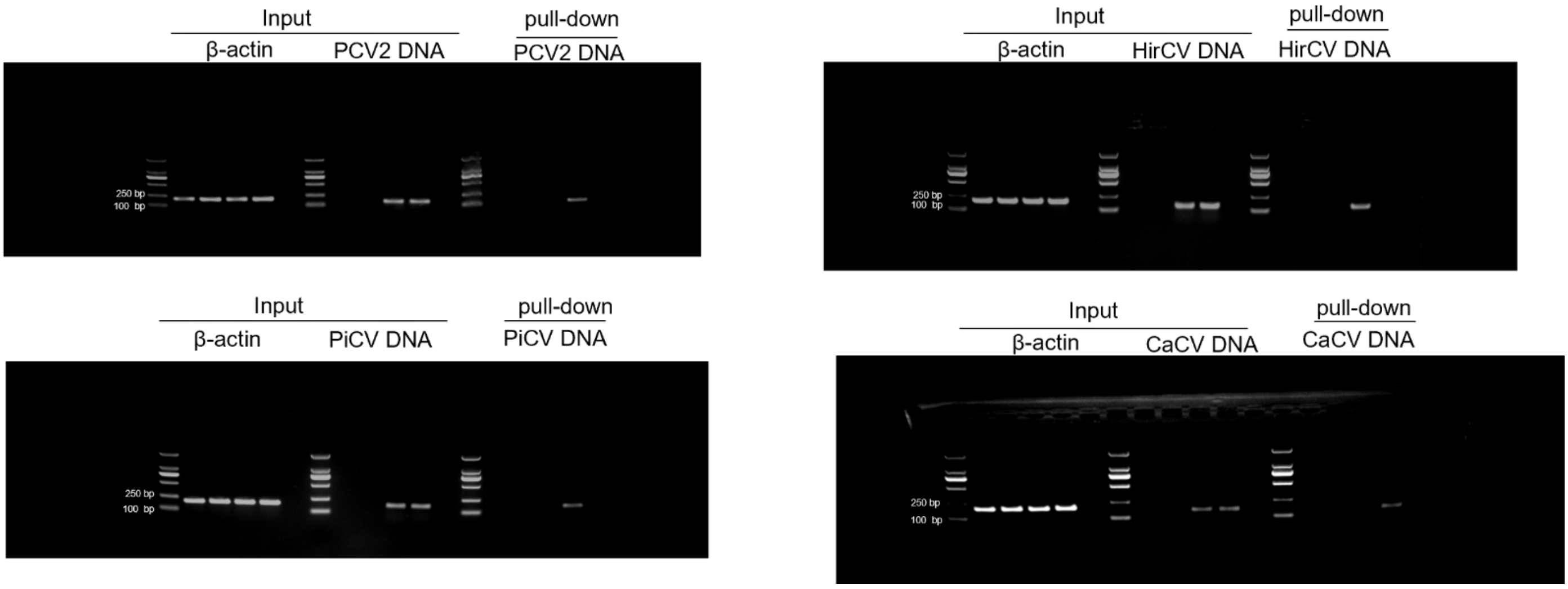

Note: The bright horizontal streak near the well line in the Figure 1D CaCV gel, observed at the very edge of the gel (outside all sample lanes and not in the middle of the gel), is an imaging artifact (edge glare/overexposure) and does not represent a specific DNA band; it has no impact on the interpretation of the experimental results.

Figure 2B

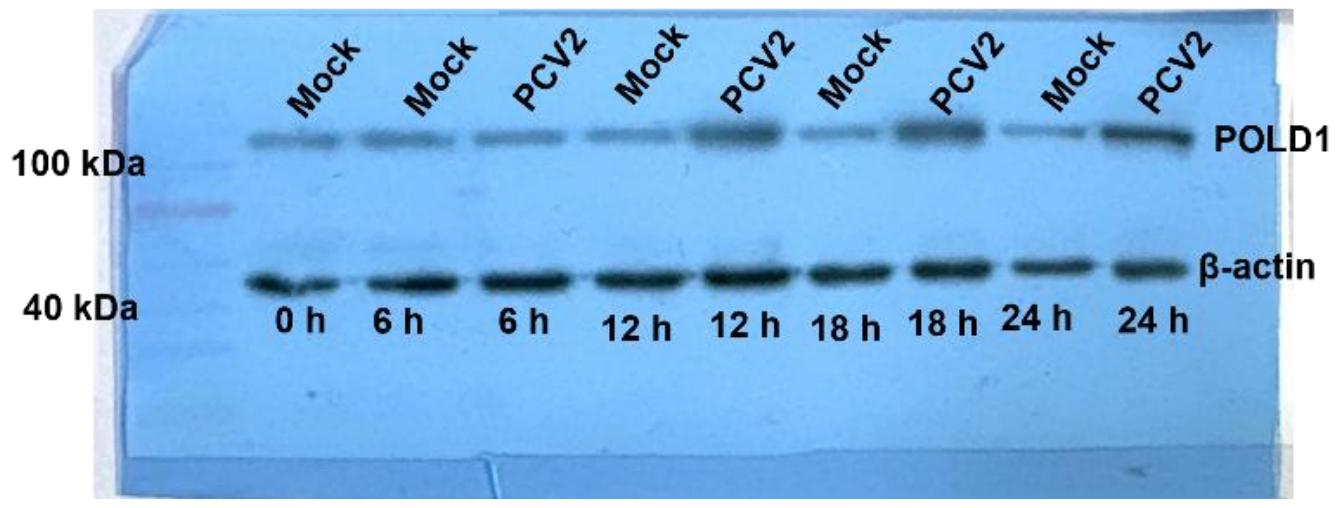

Figure 2D

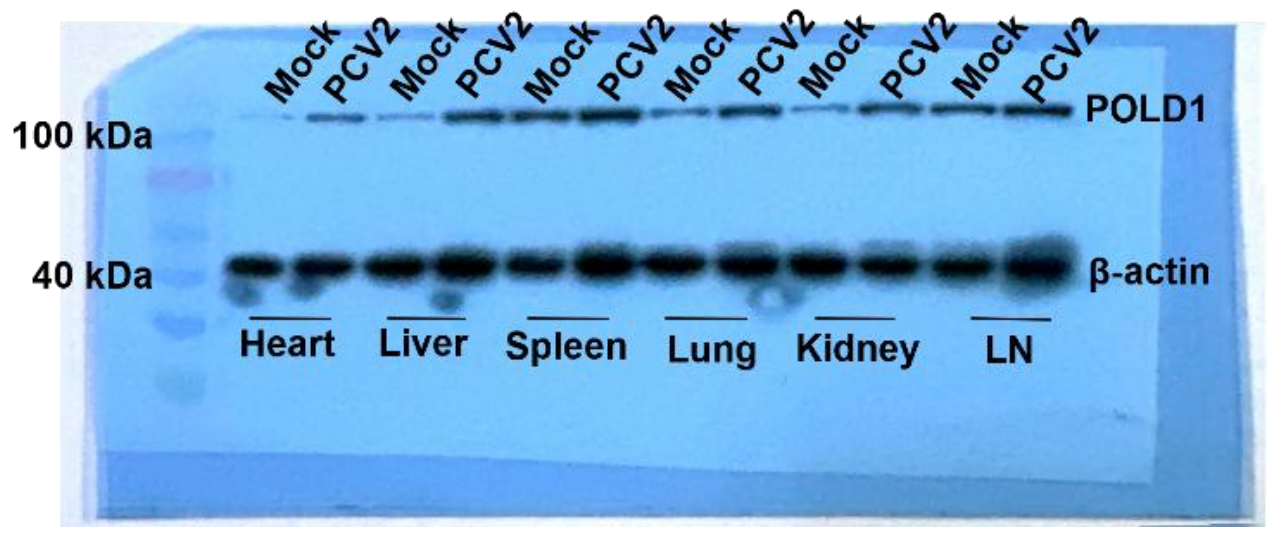

Figure 3B

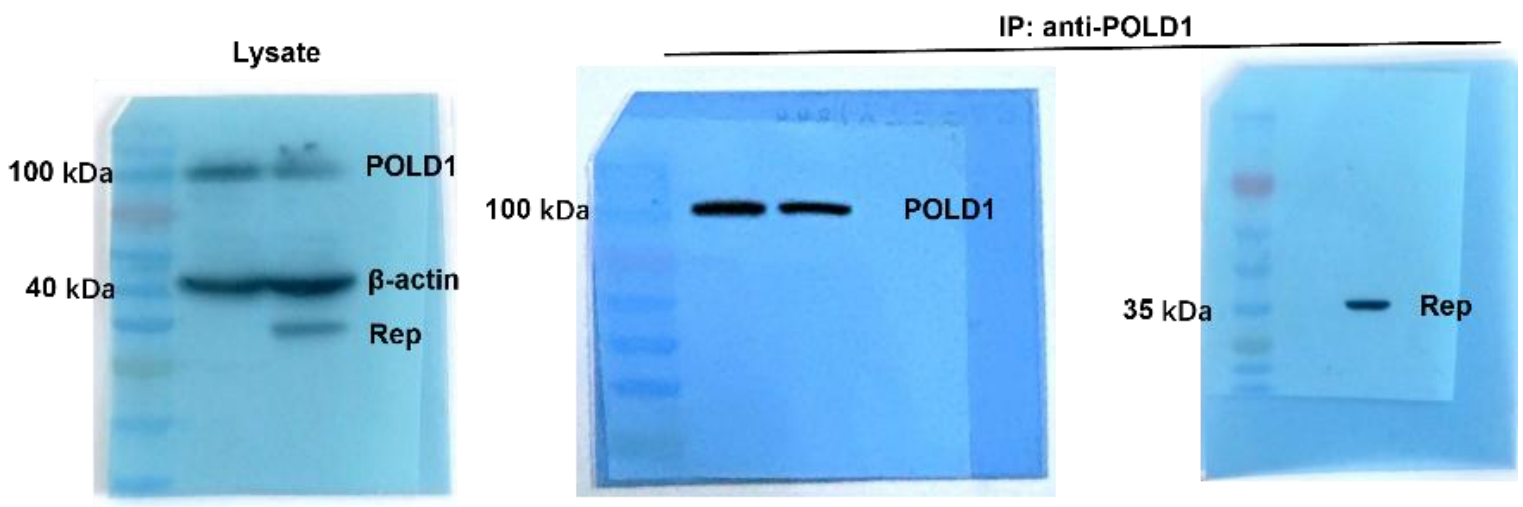

Figure 3C

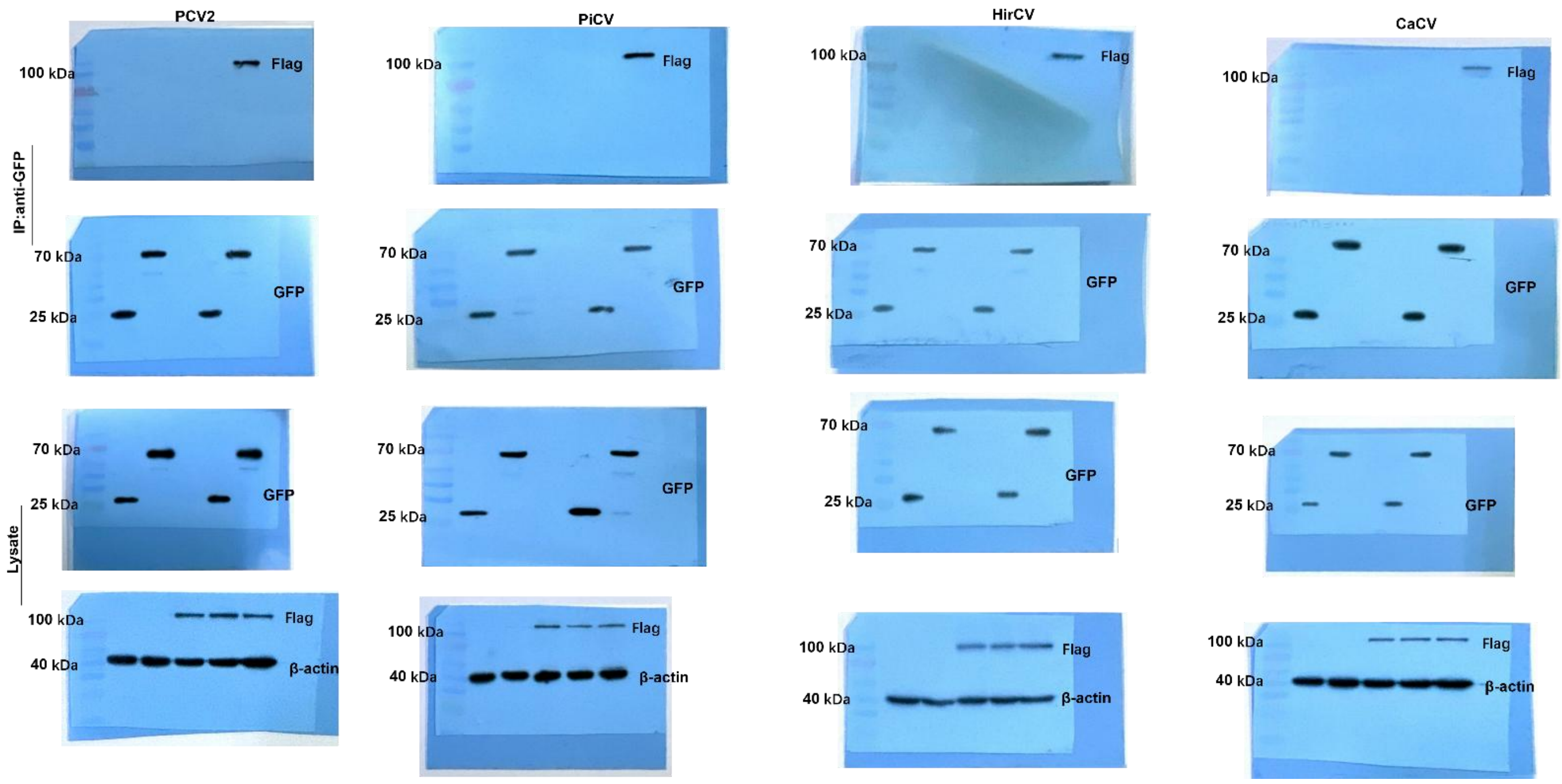

Figure 3D

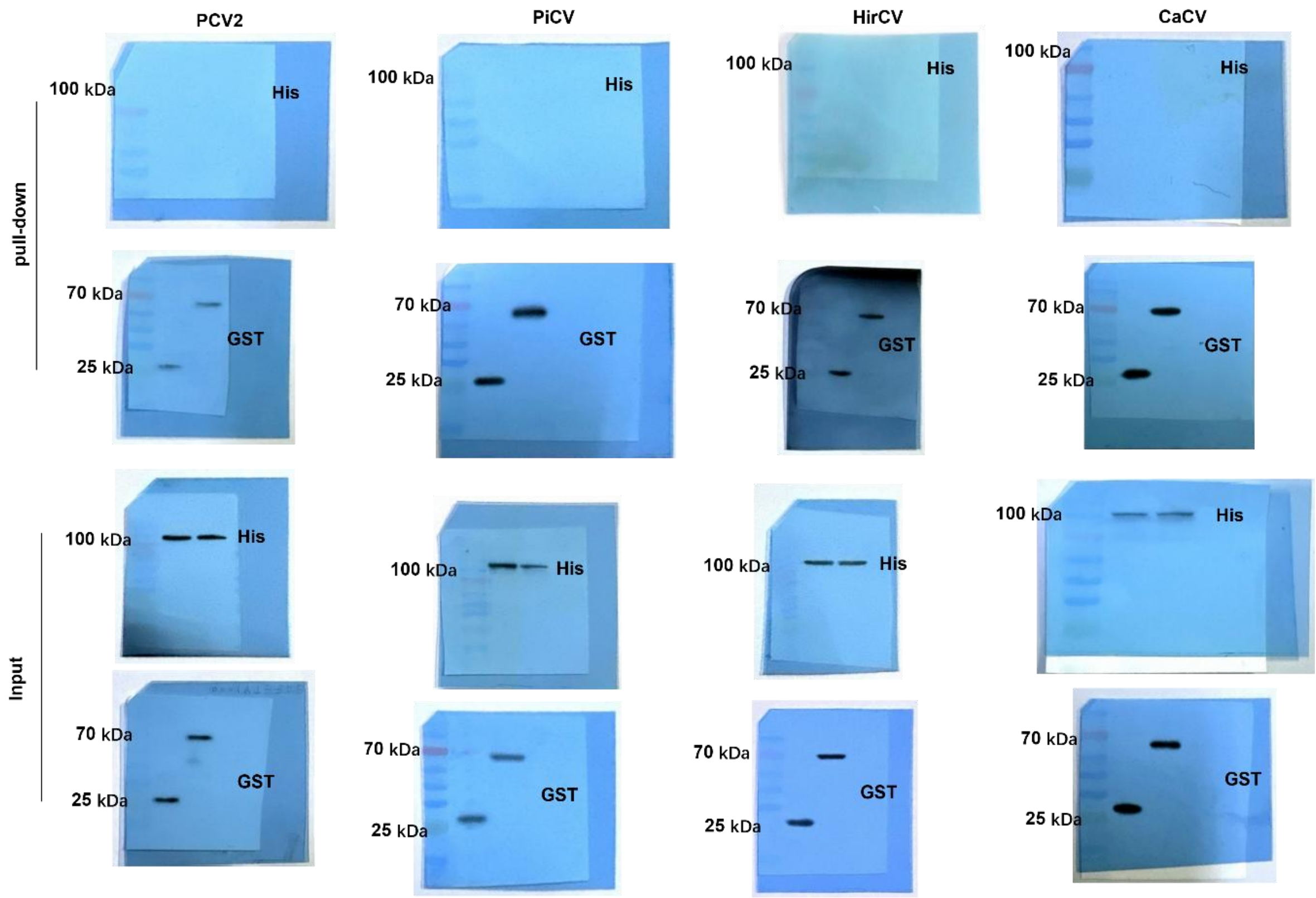

Figure 4B

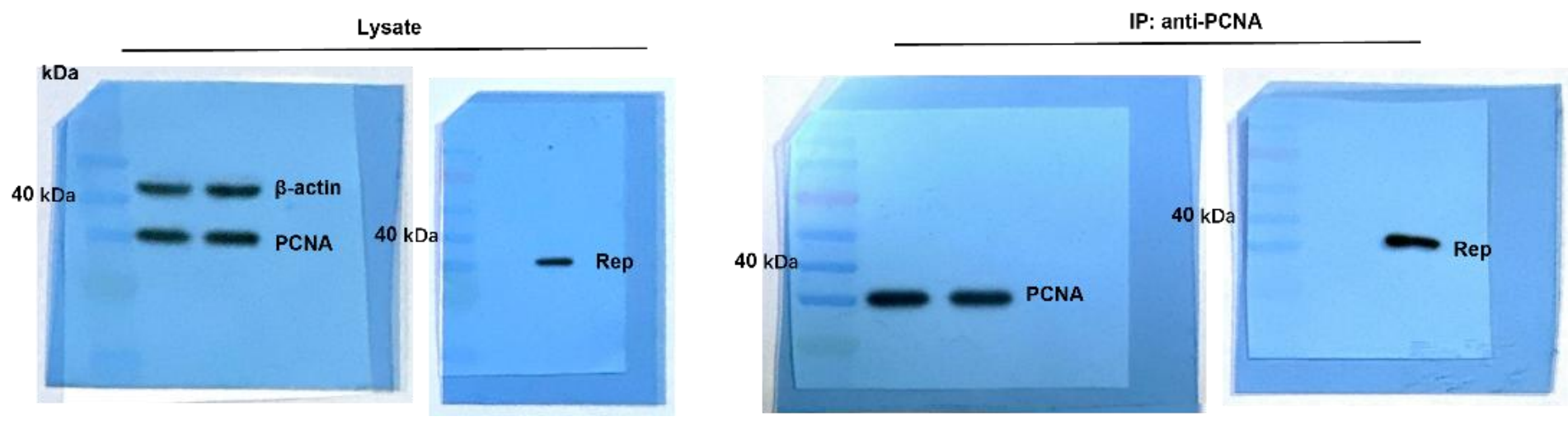

Figure 4C

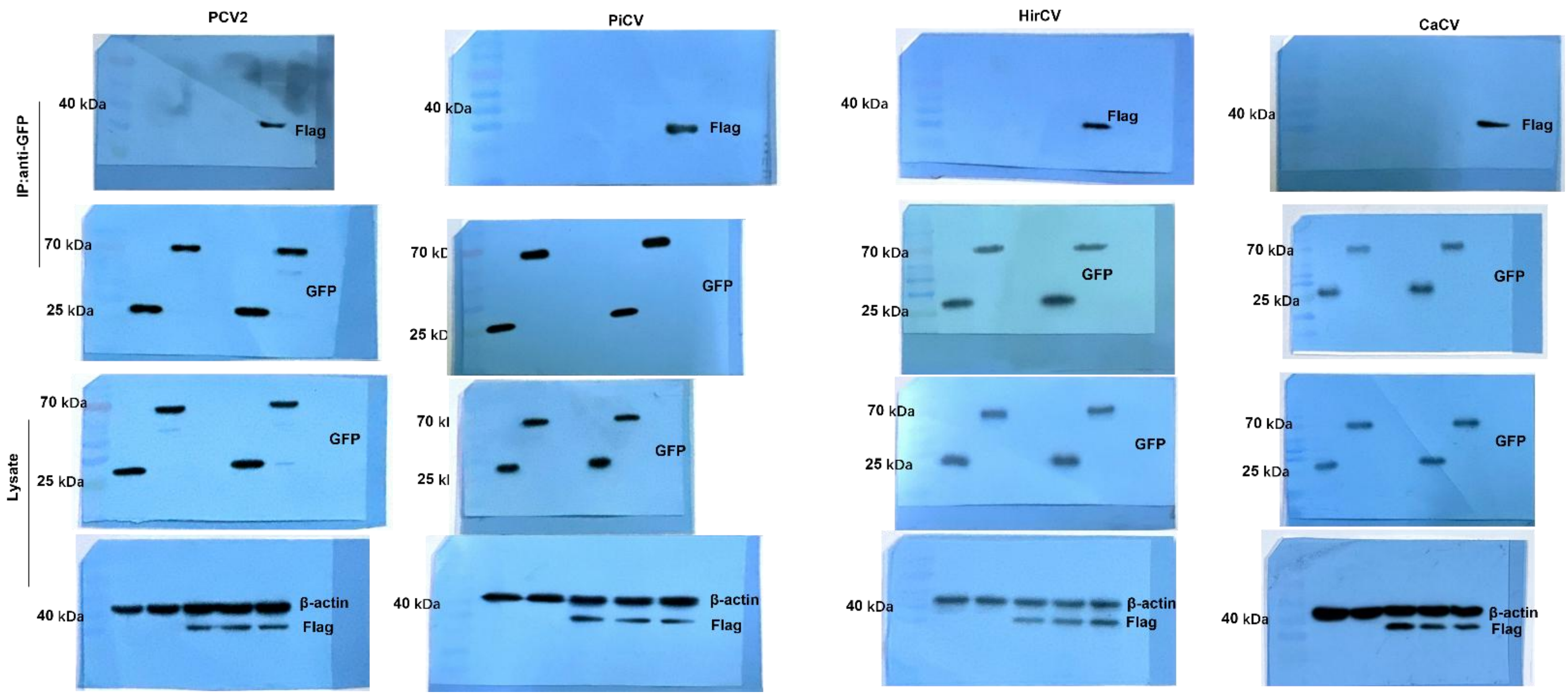

Figure 4D

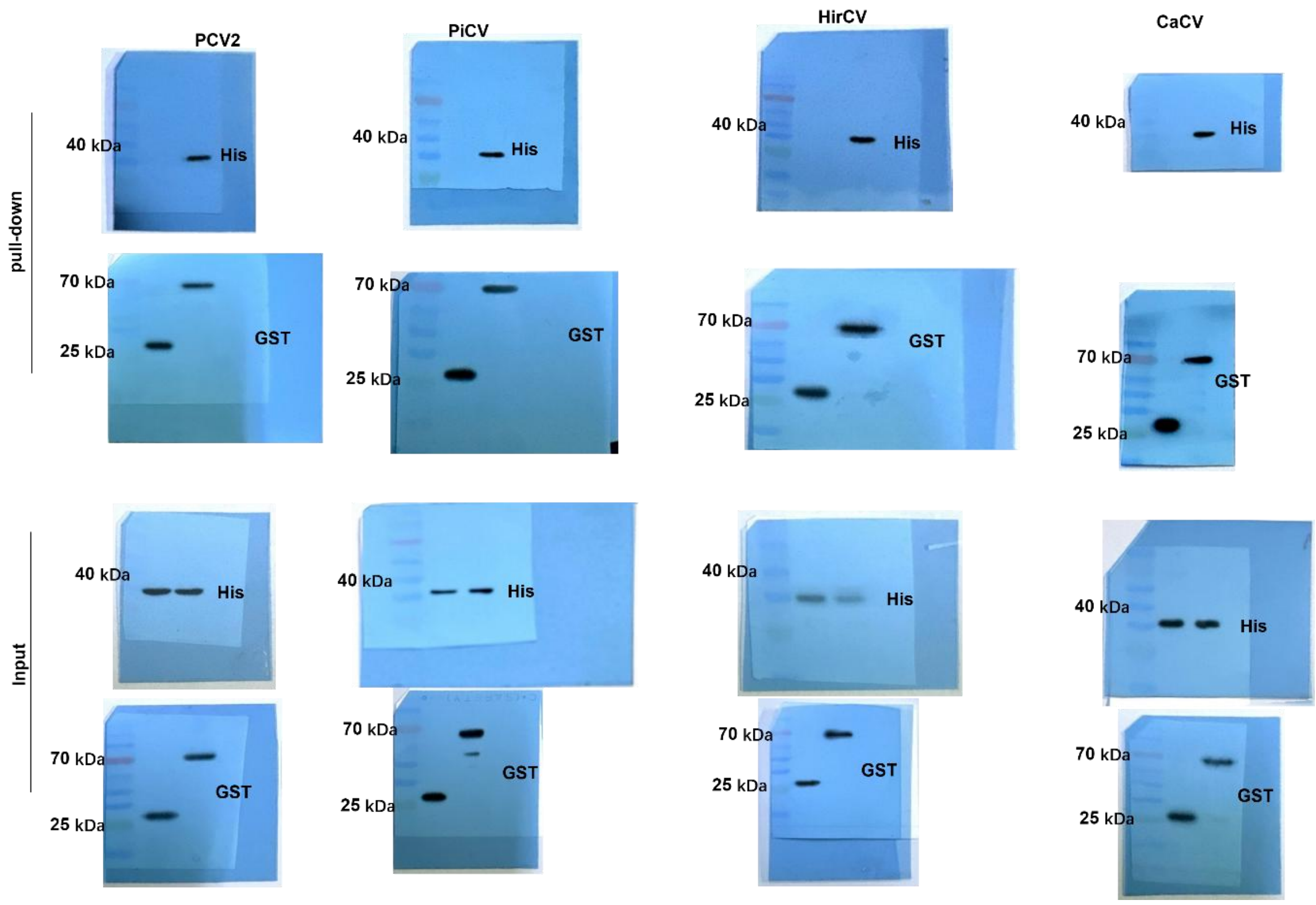

Figure 4E

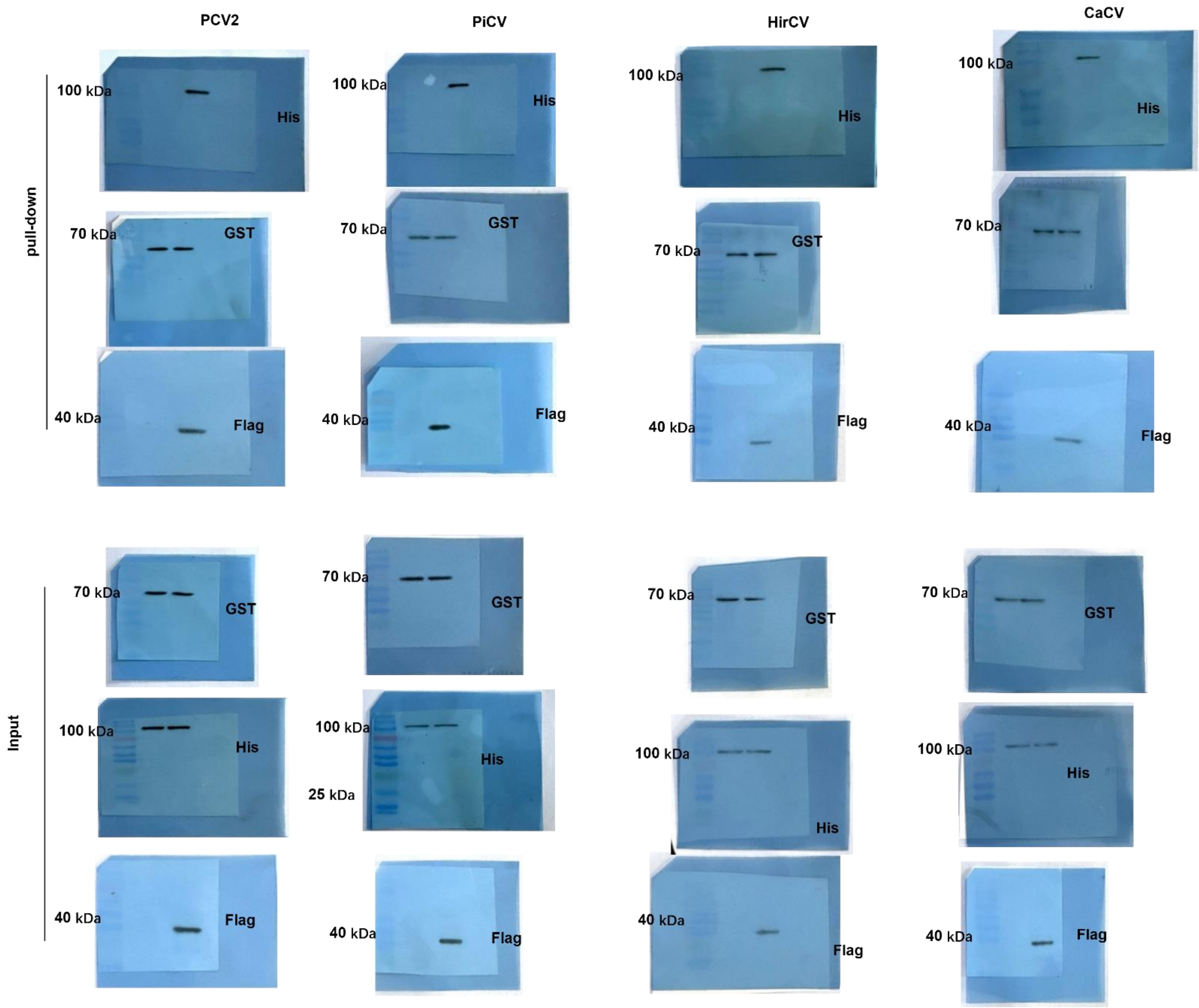

Figure 4F

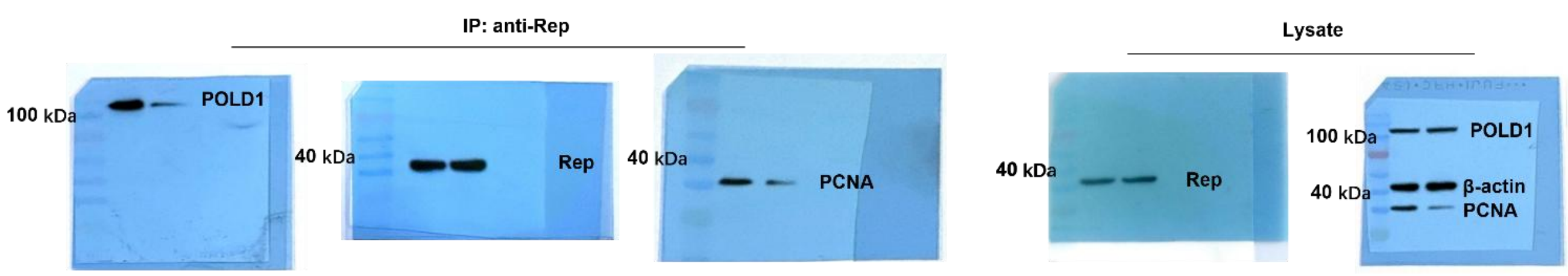

Figure 5B

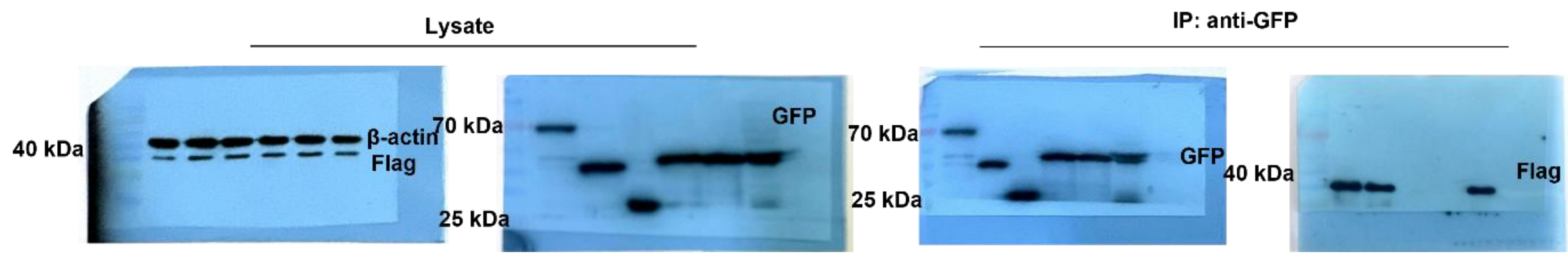

Figure 5D

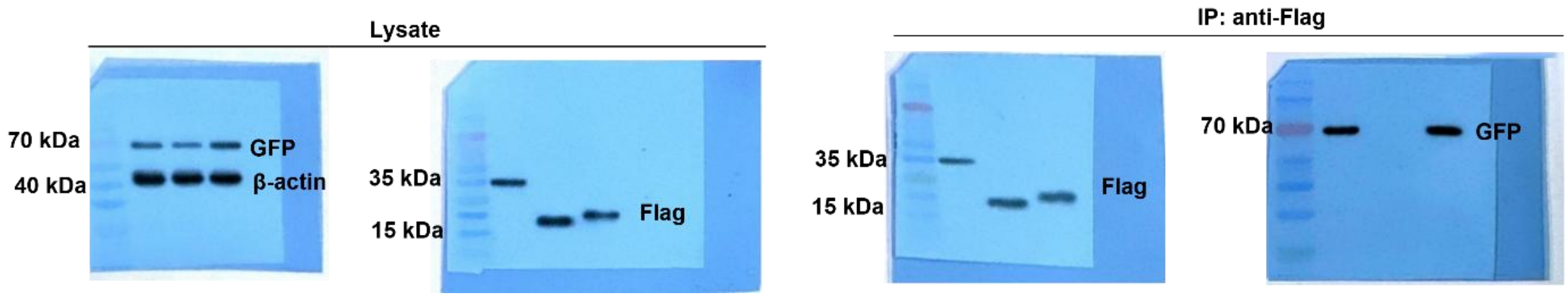

Figure 5F

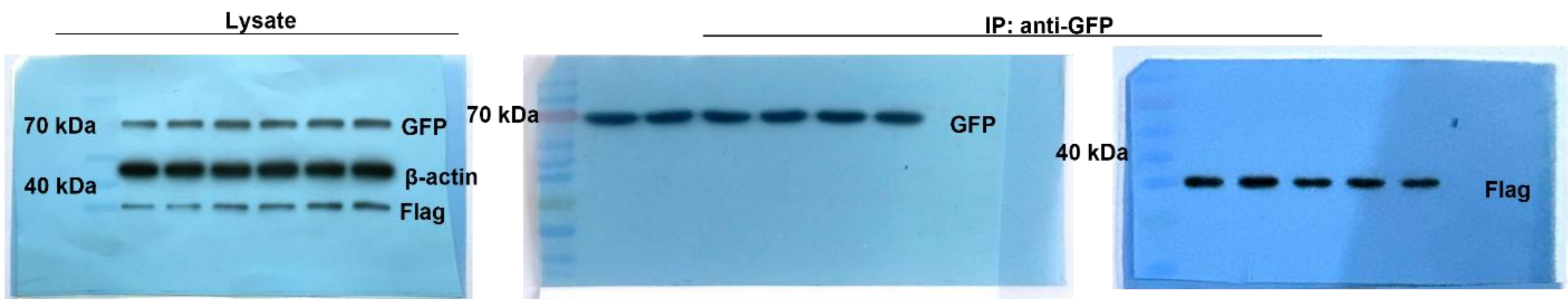

Figure 5G

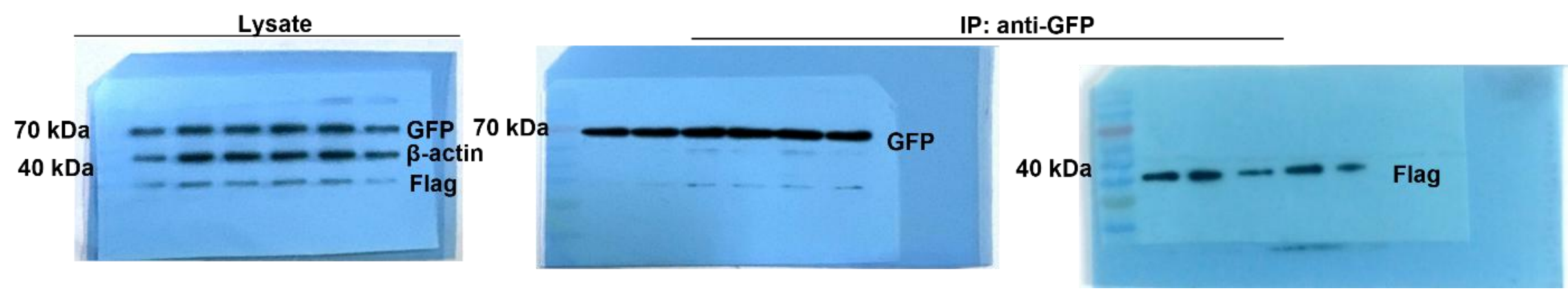

Figure 5H

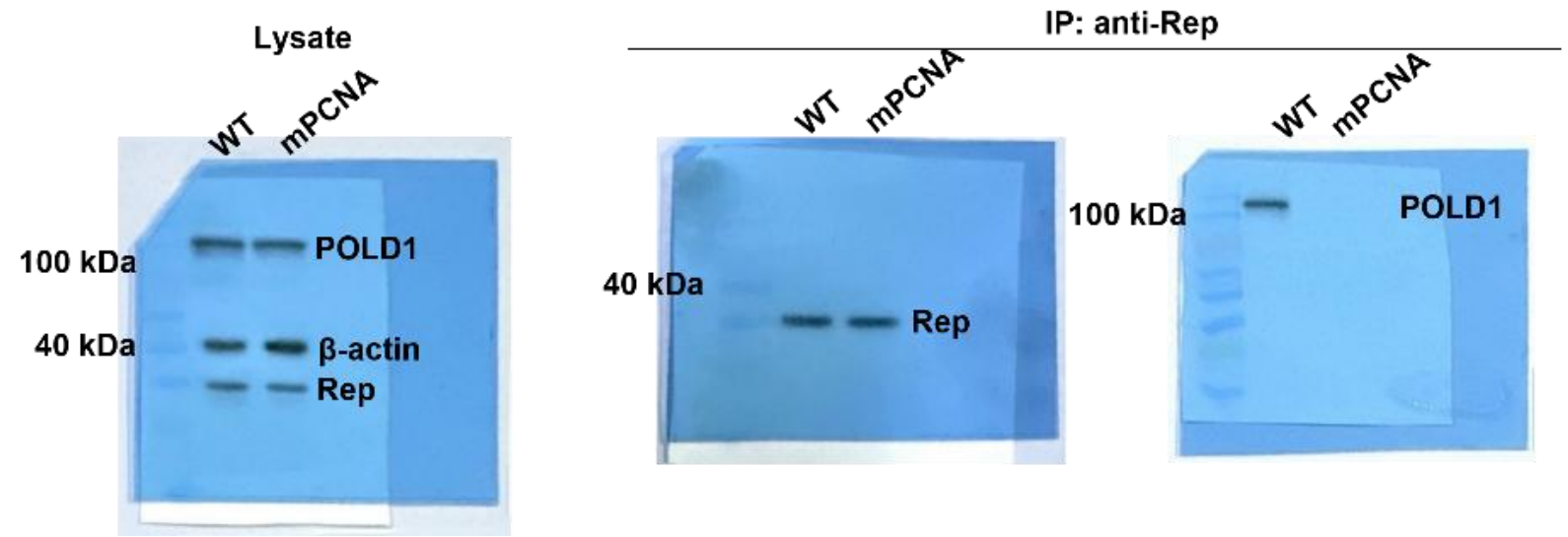

Figure 5I

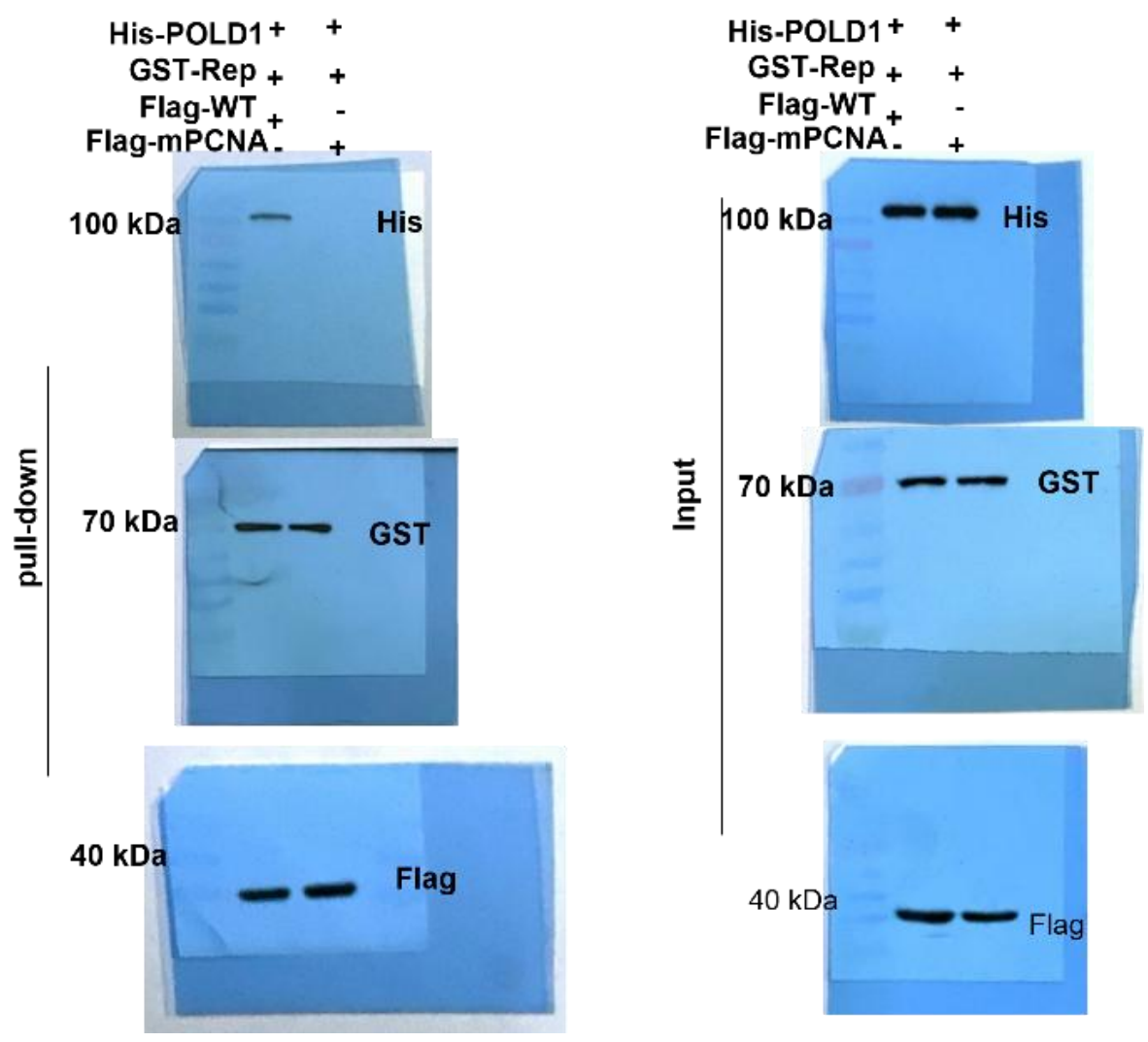

Figure 6F

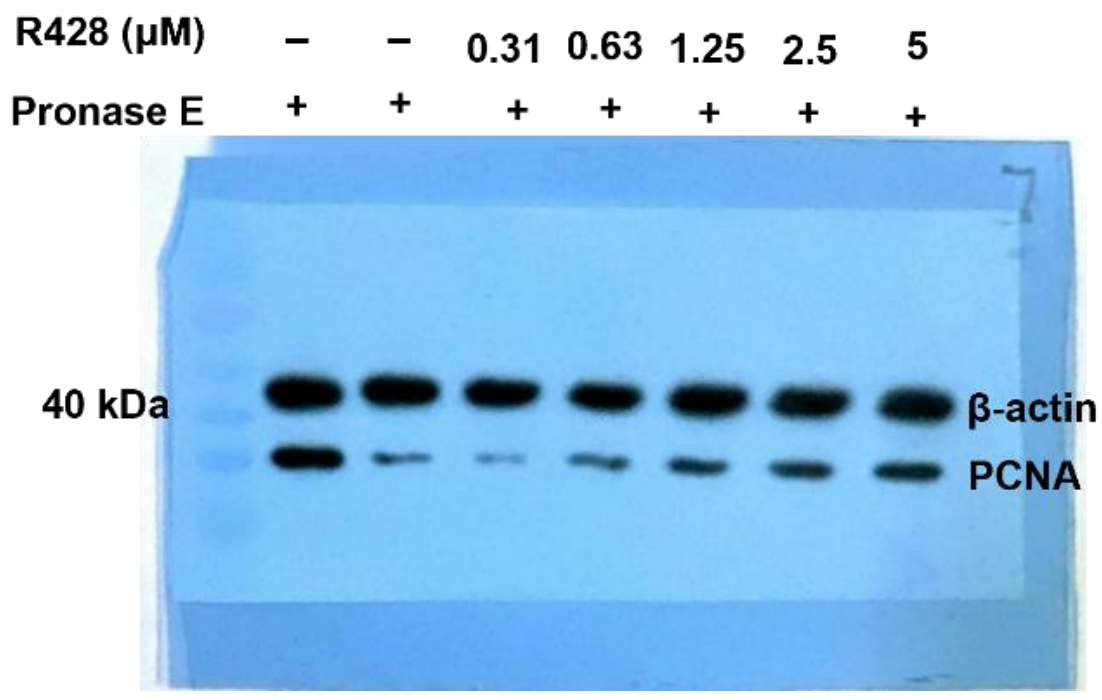

Figure 6G

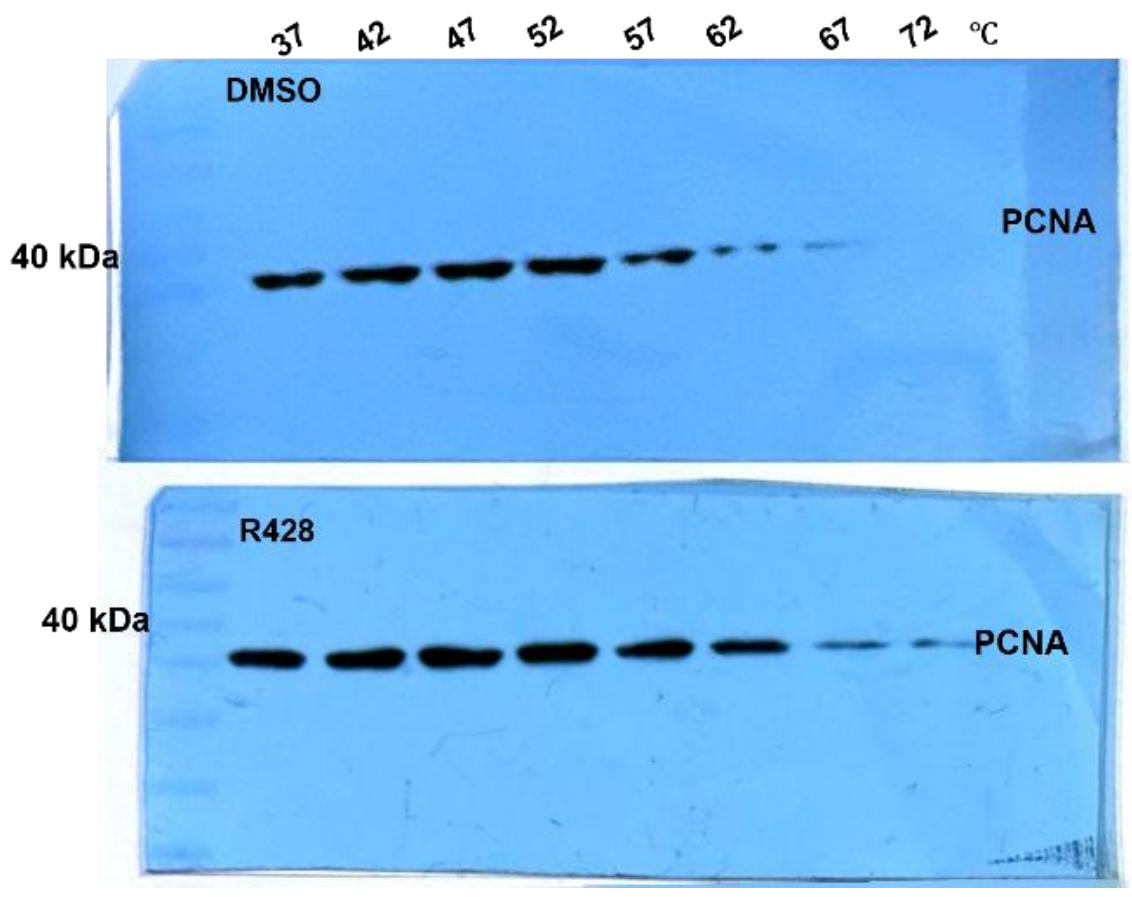

Figure 6J

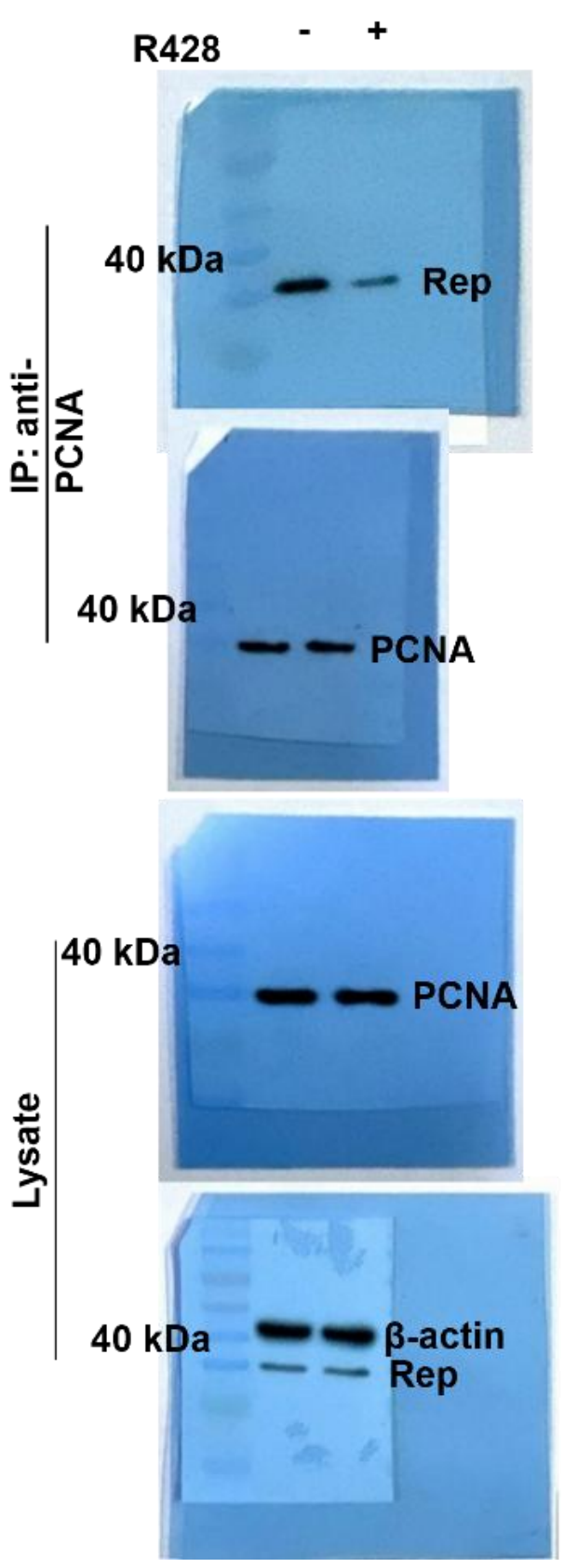

Figure 6K

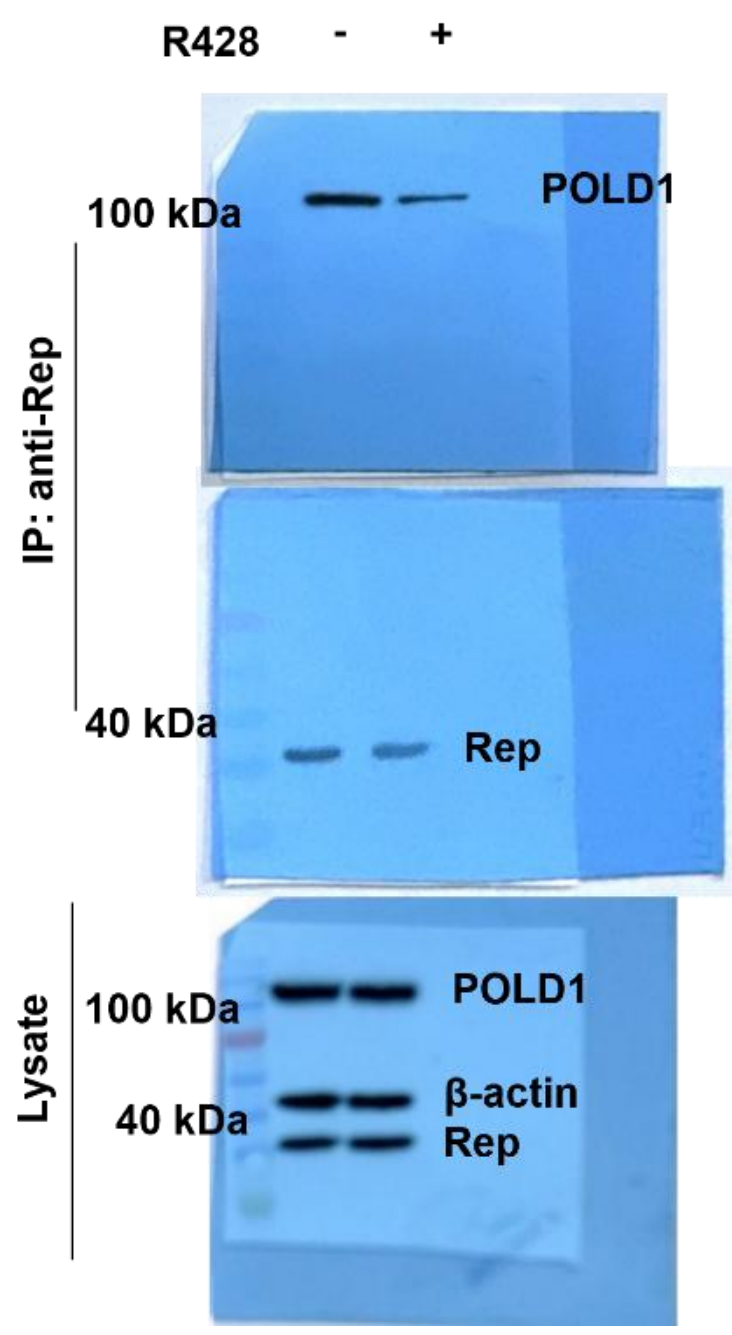

Supplementary Figure 1C

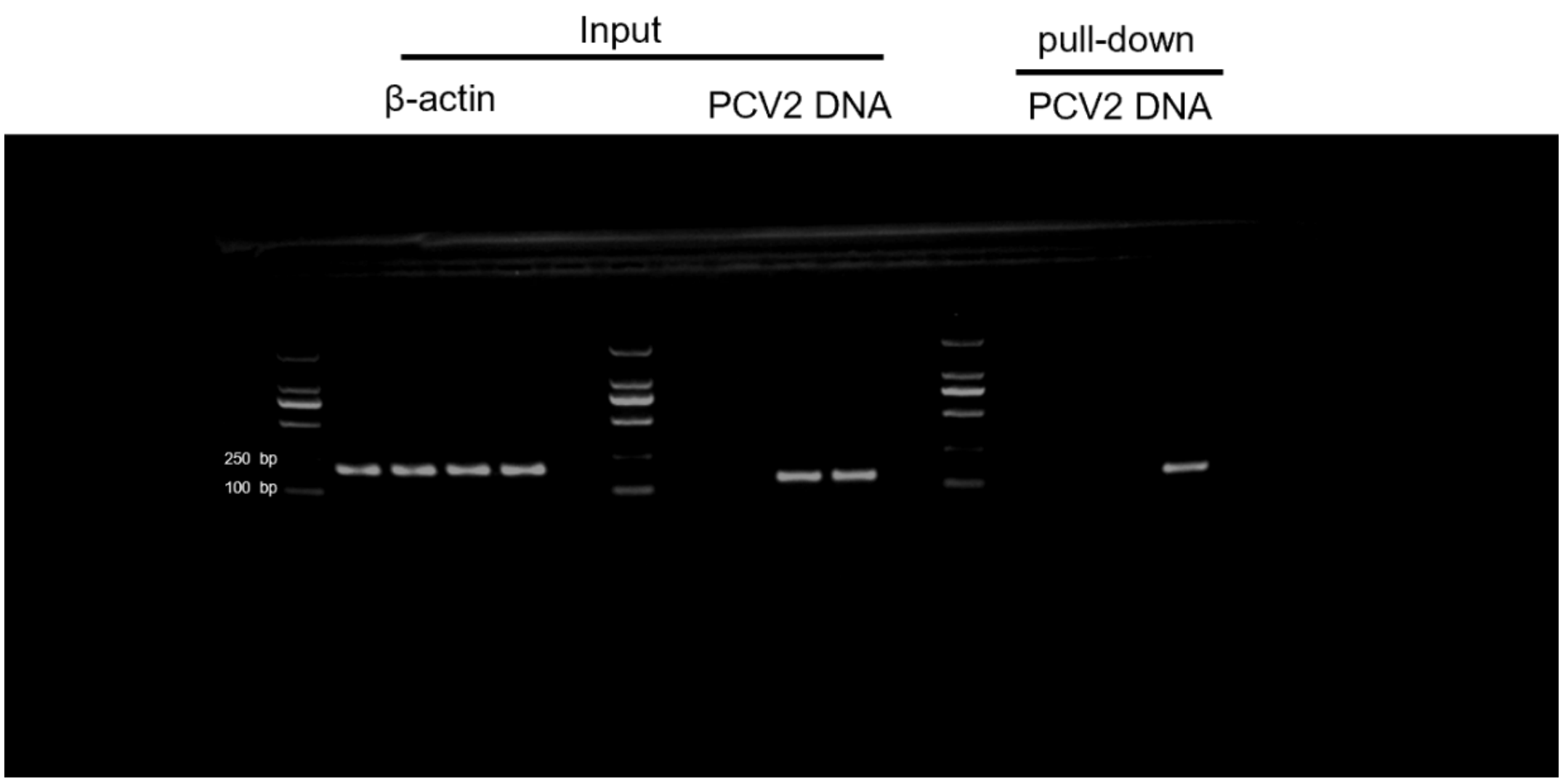

Supplementary Figure 2A

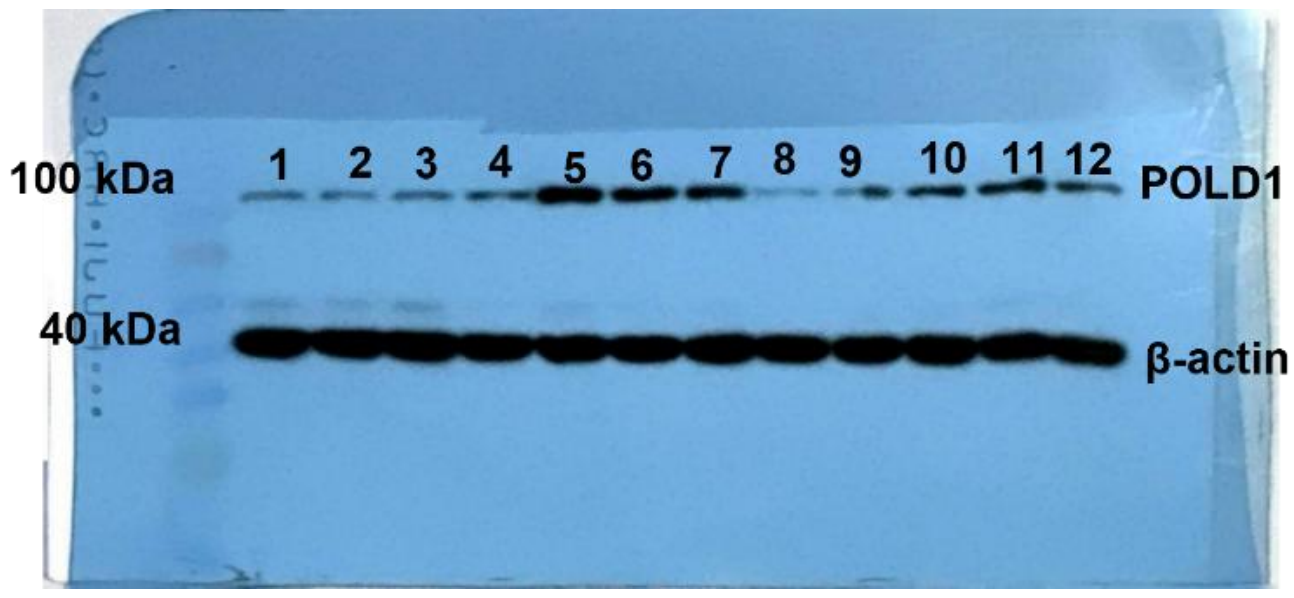

Supplementary Figure 2B

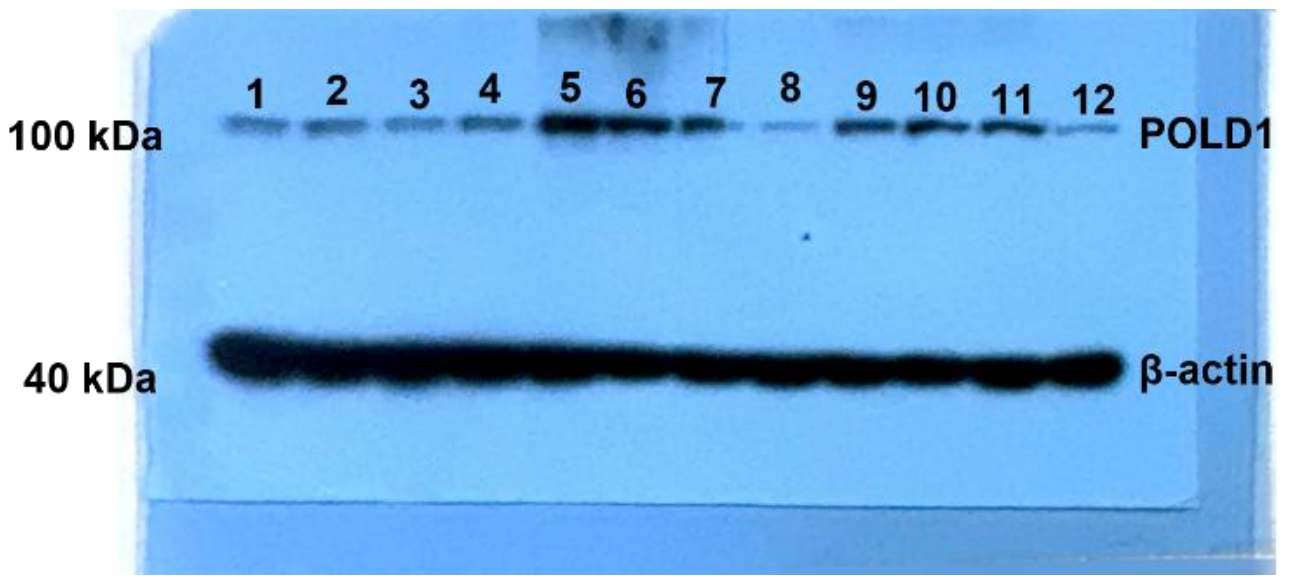

Supplementary Figure 3A

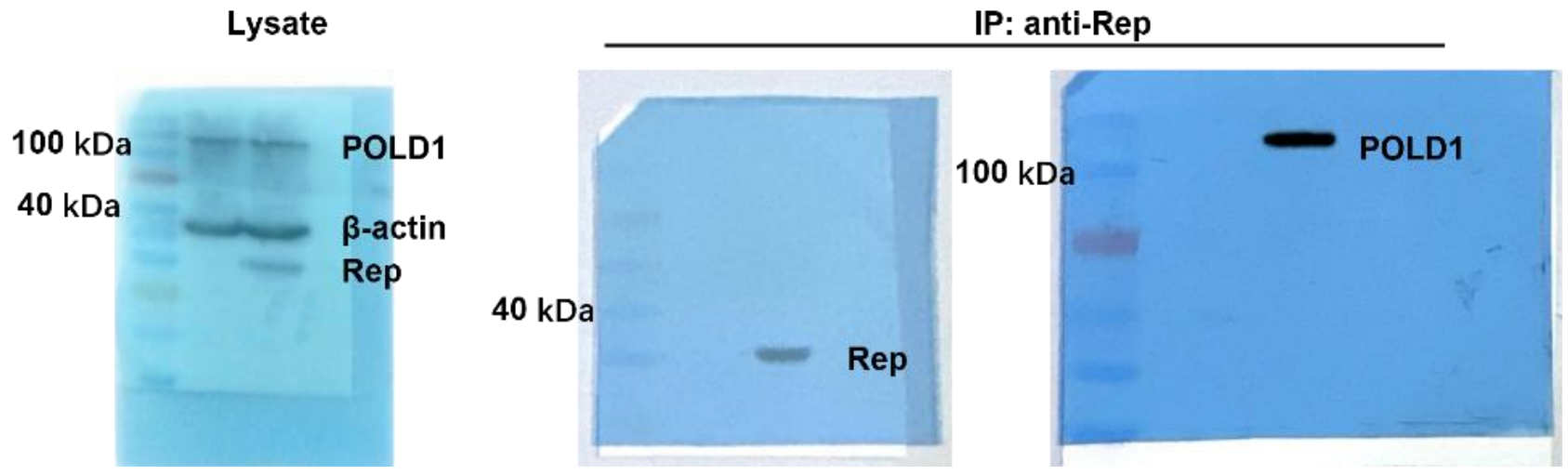

Supplementary Figure 3B

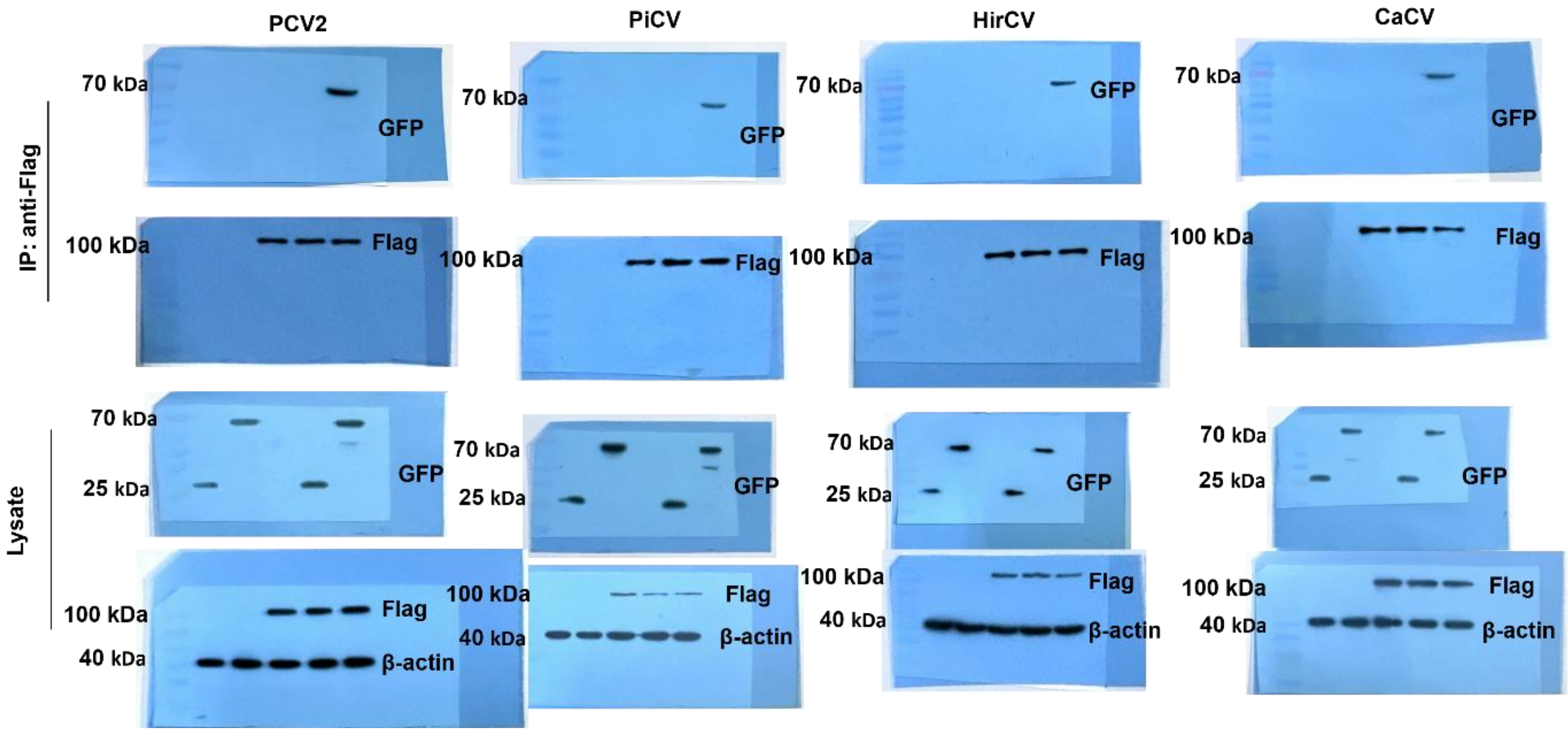

Supplementary Figure 3C

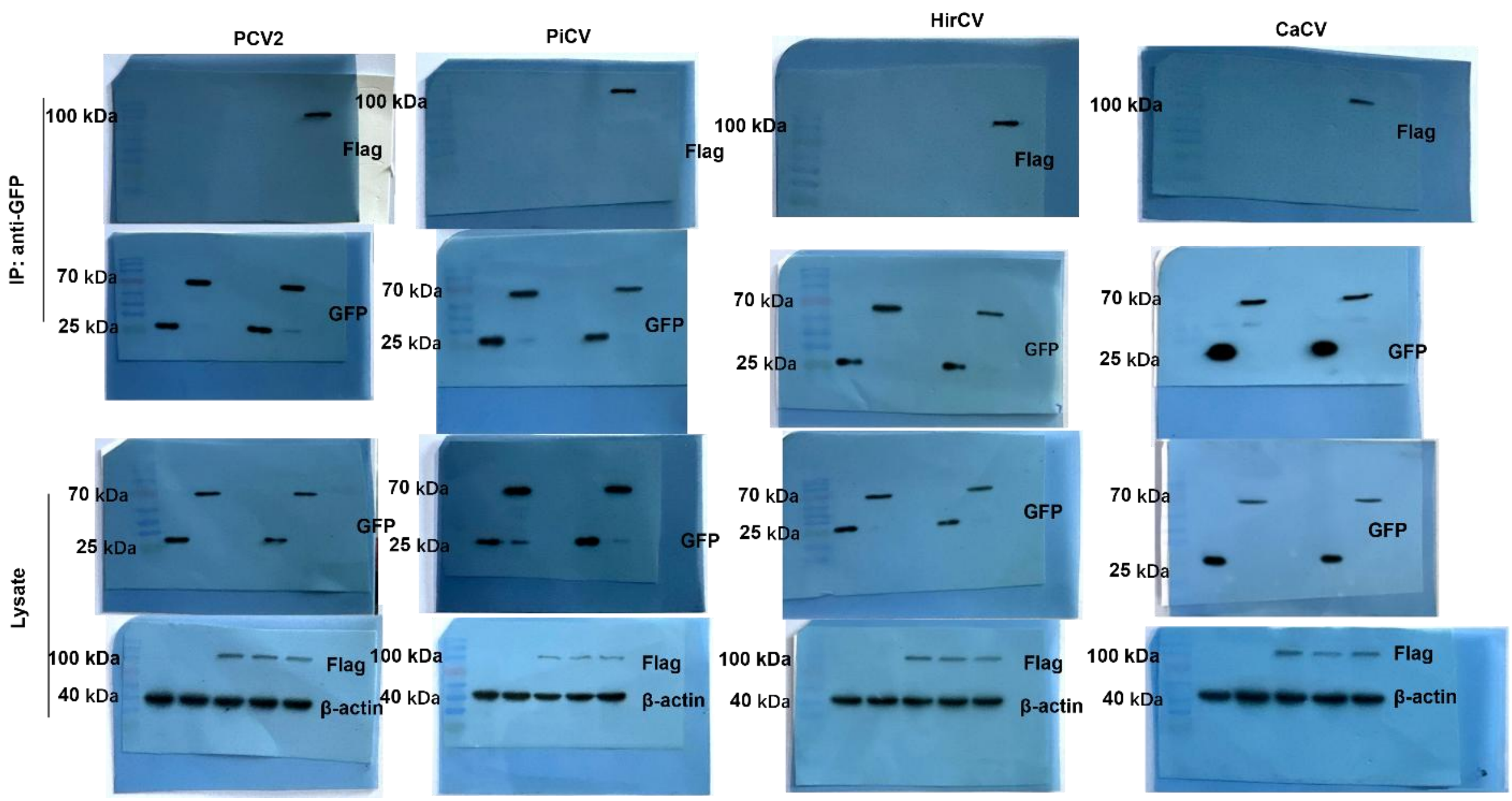

Supplementary Figure 3D

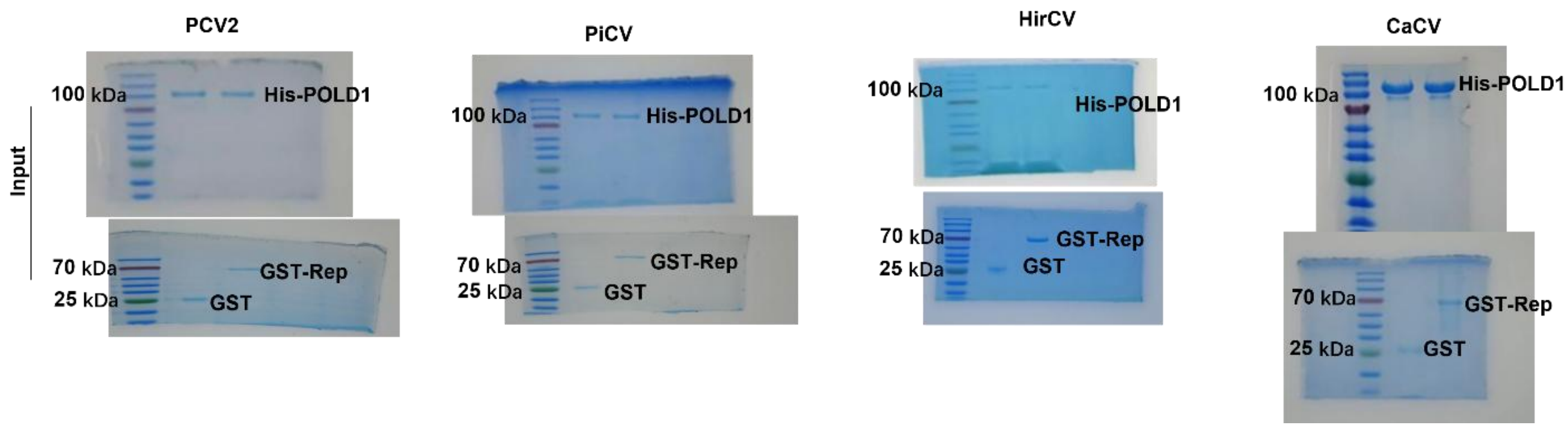

Supplementary Figure 4A

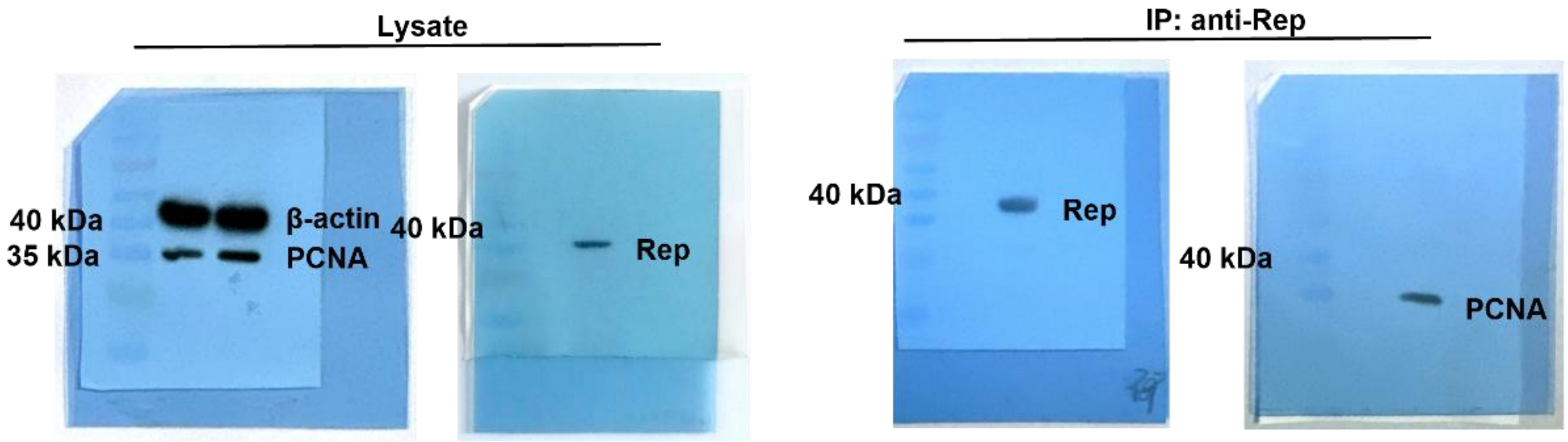

Supplementary Figure 4B

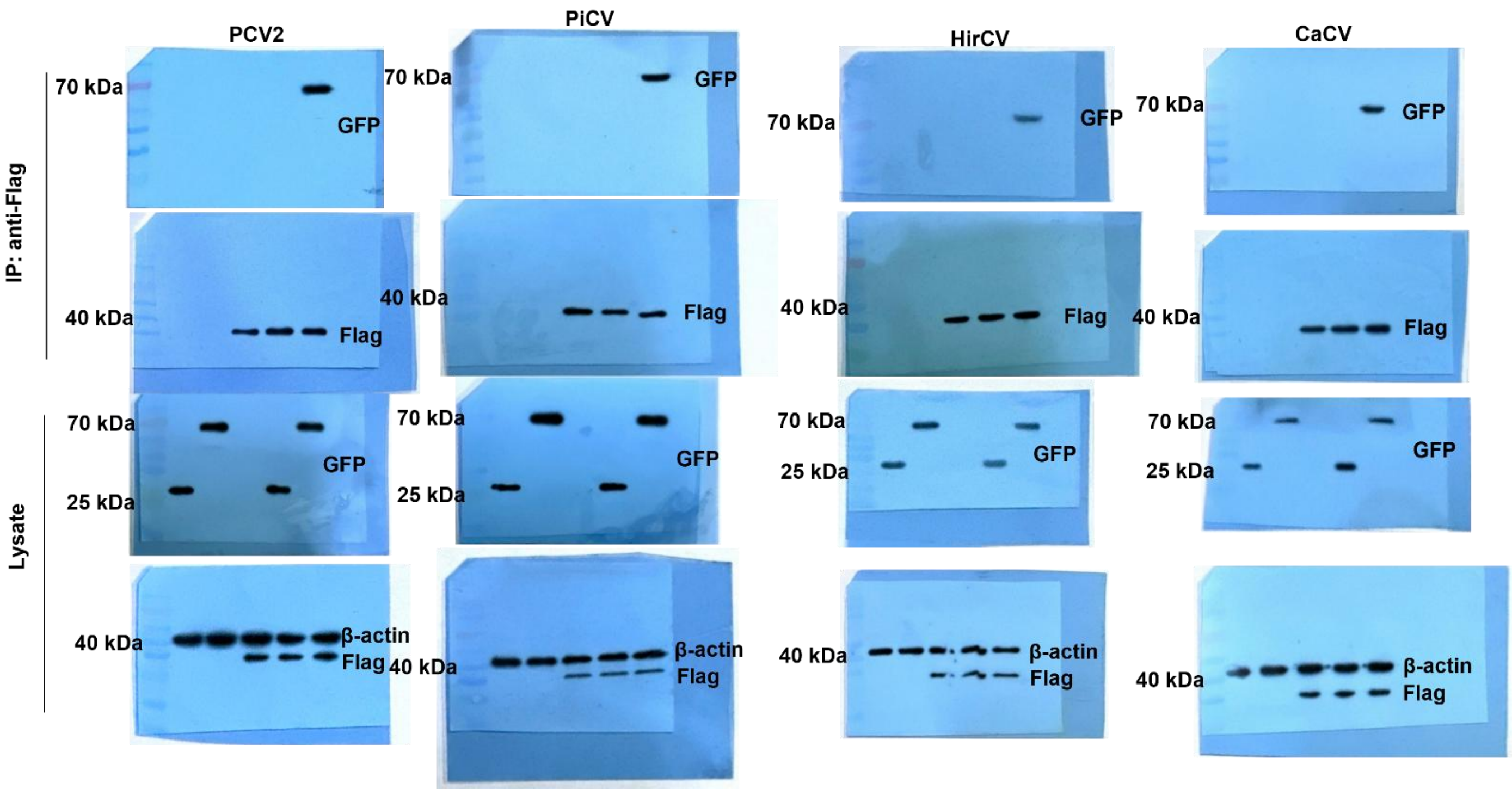

Supplementary Figure 4C

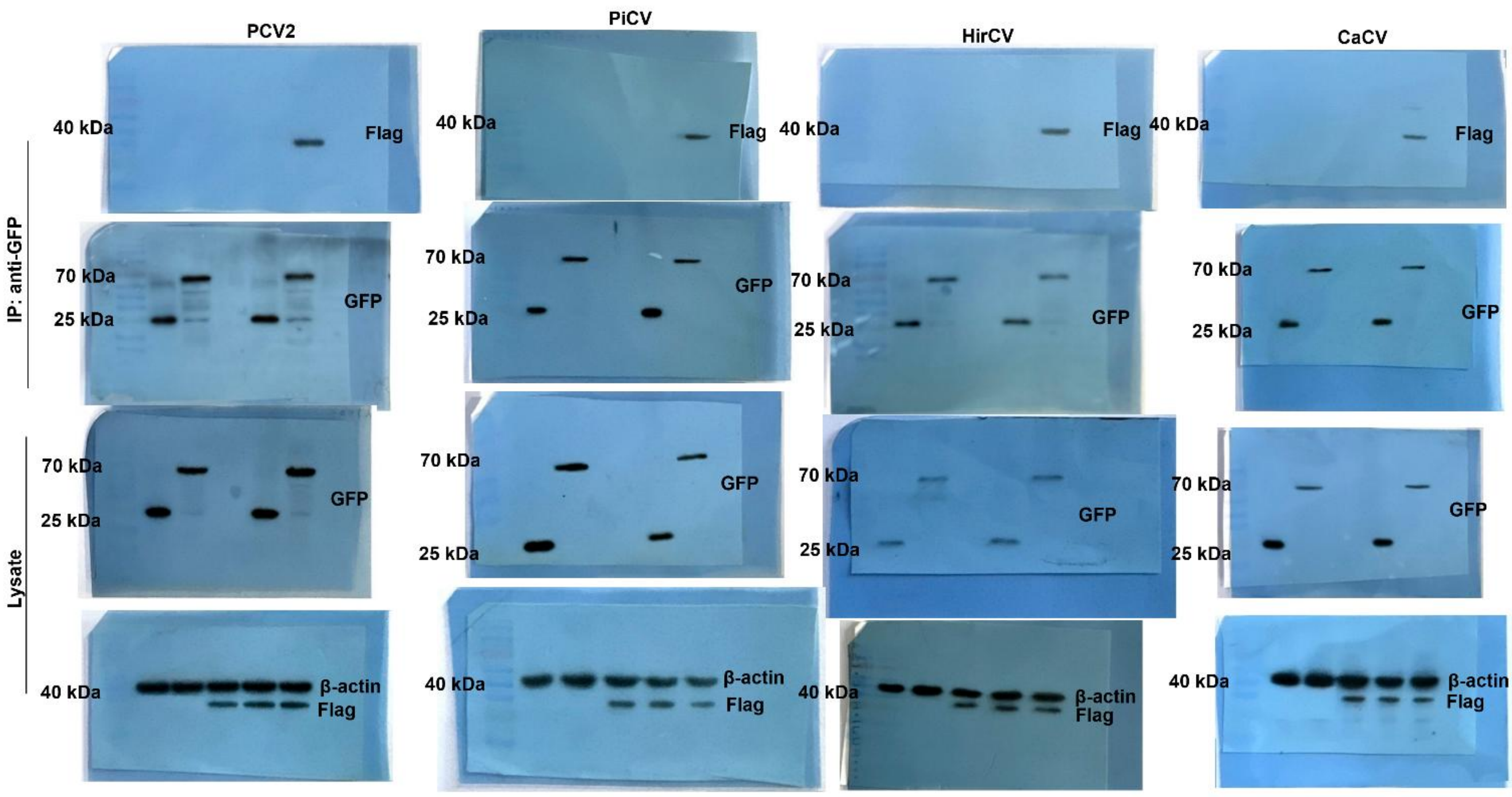

Supplementary Figure 4D

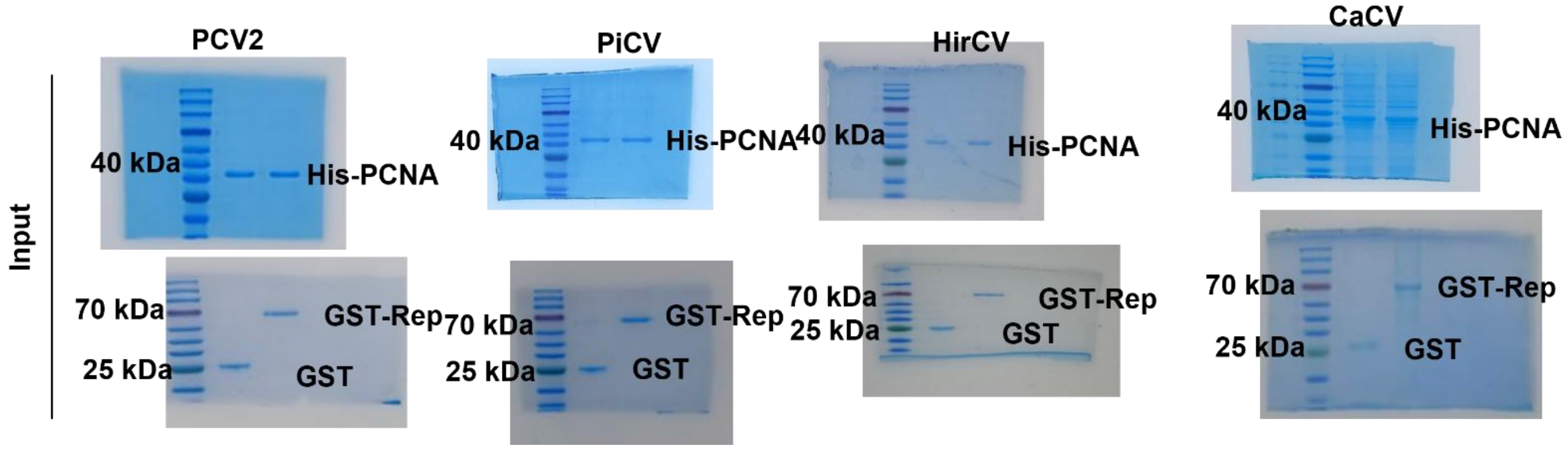

Supplementary Figure 4E

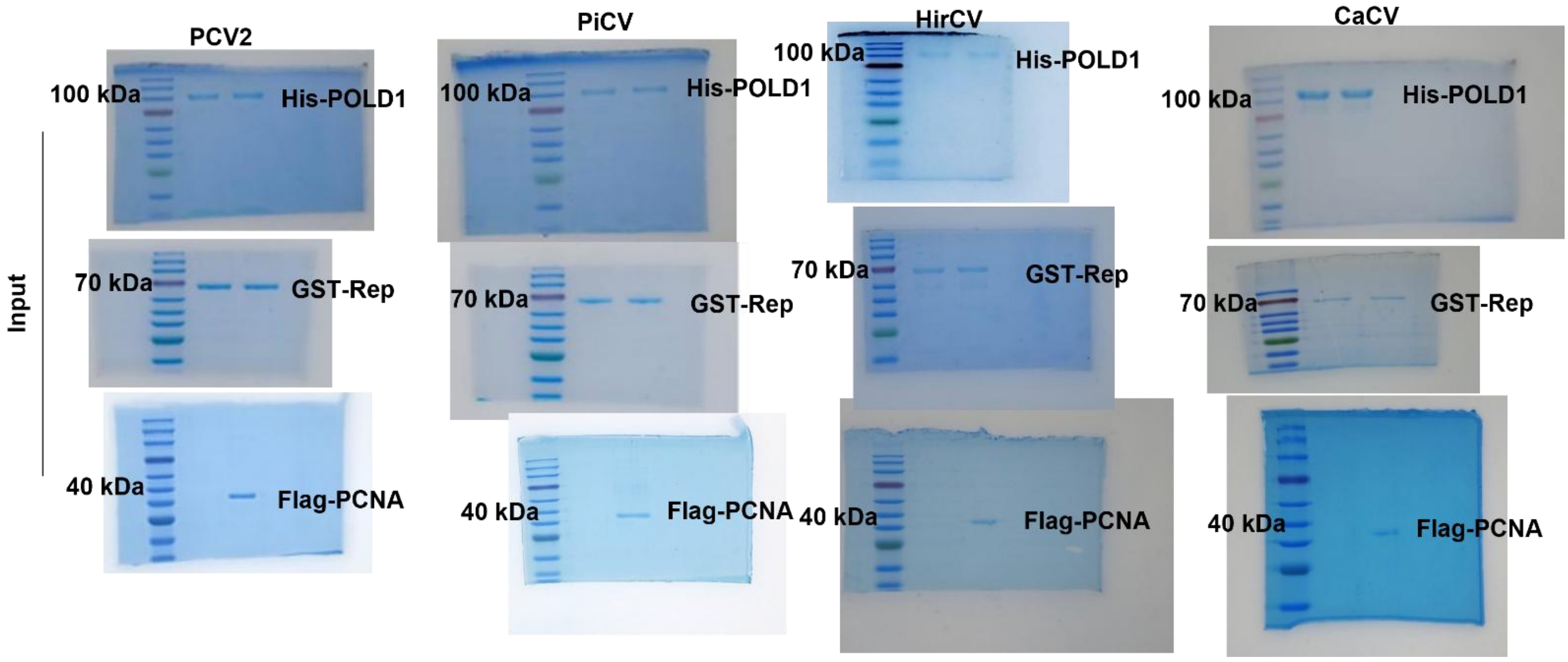

Supplementary Figure 4F

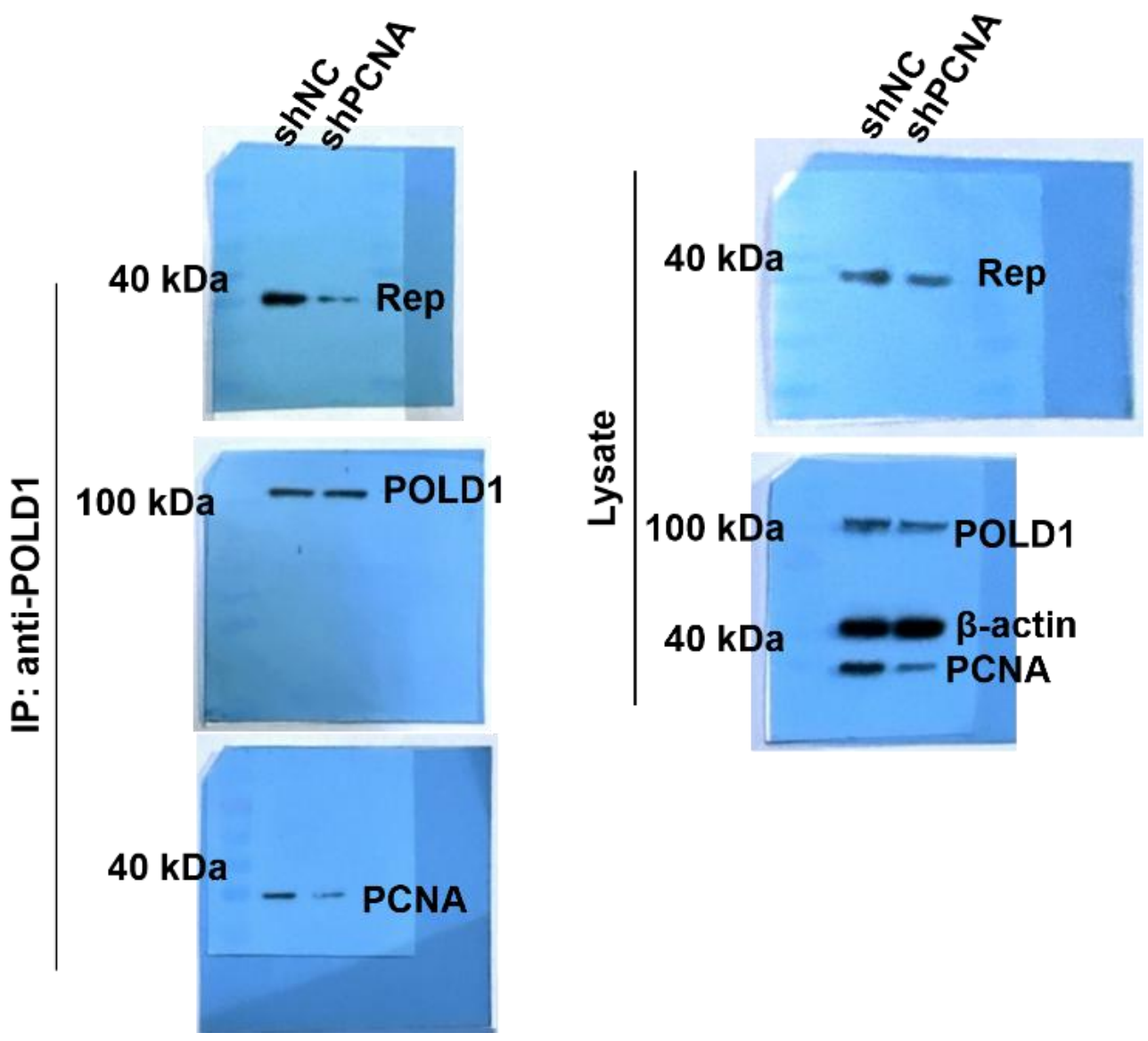

Supplementary Figure 5E

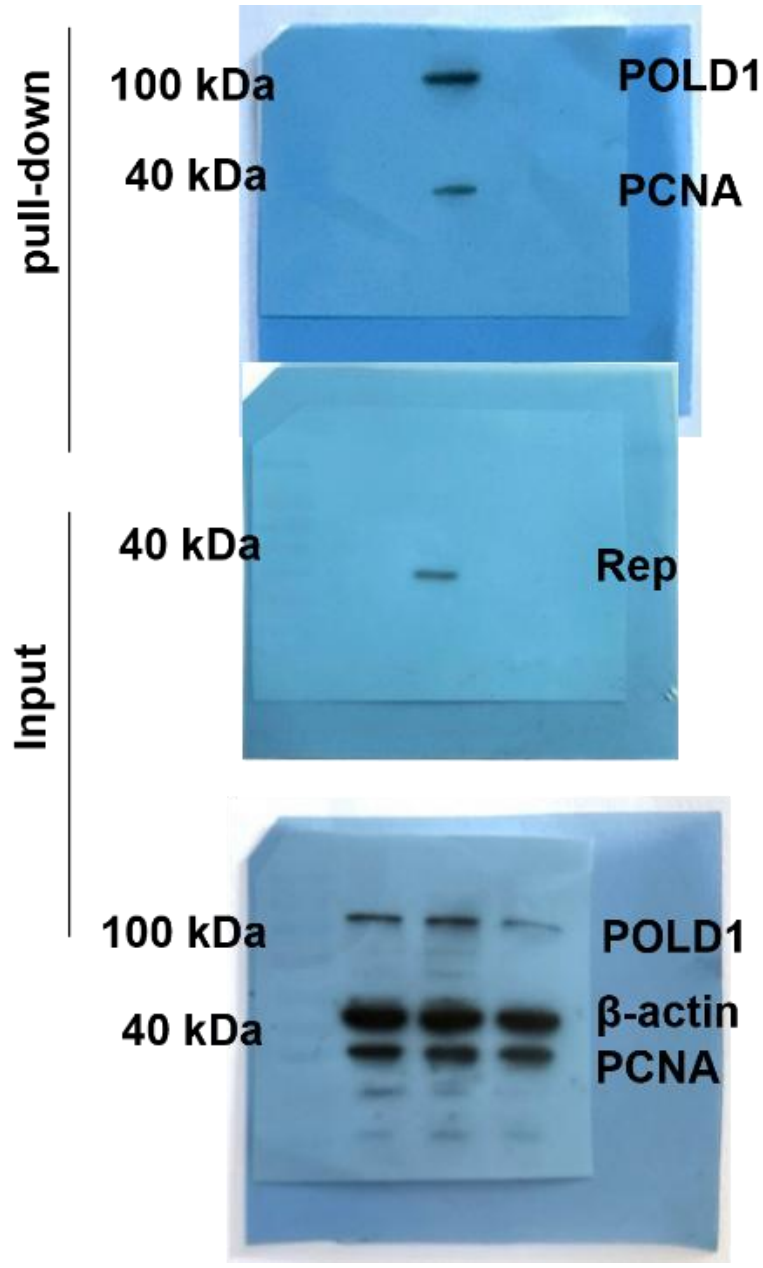

Supplementary Figure 5F

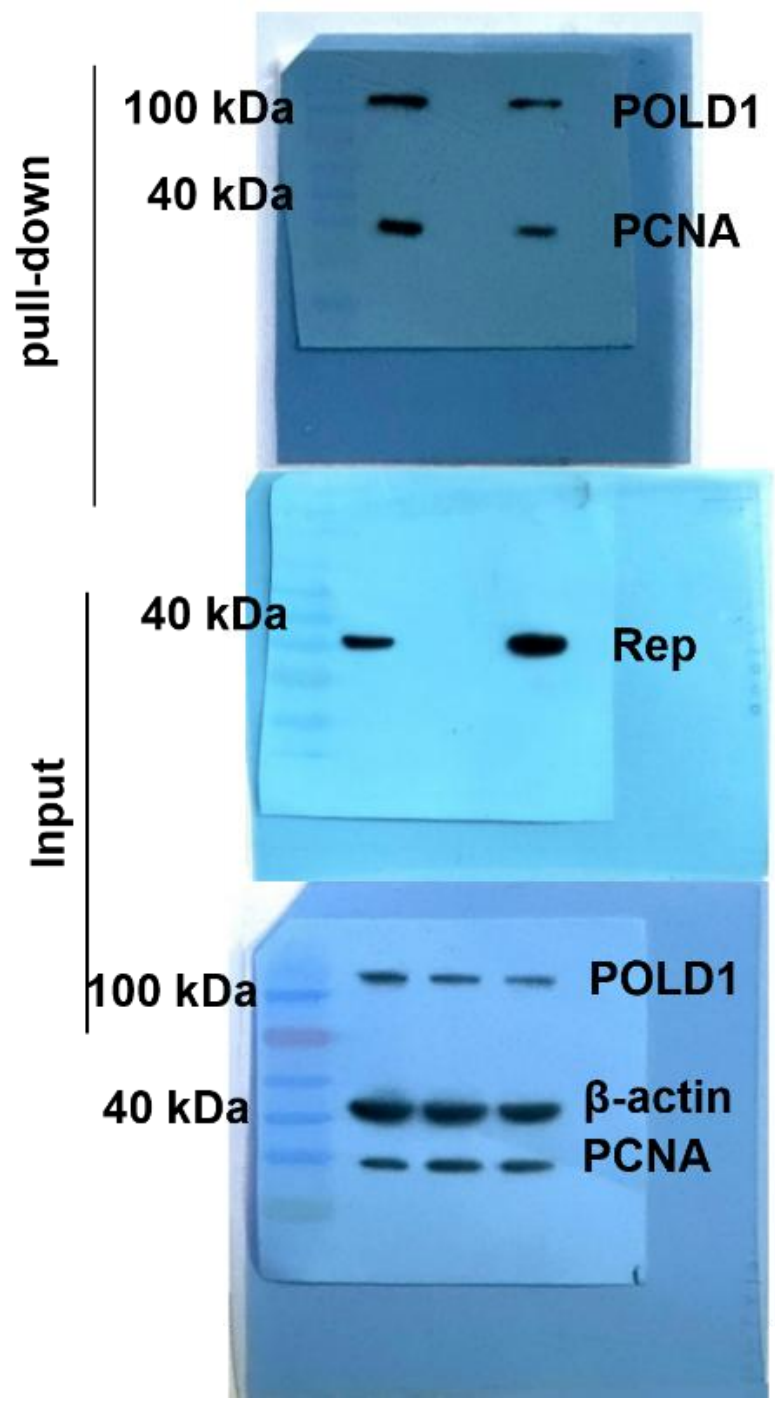

Supplementary Figure 6A

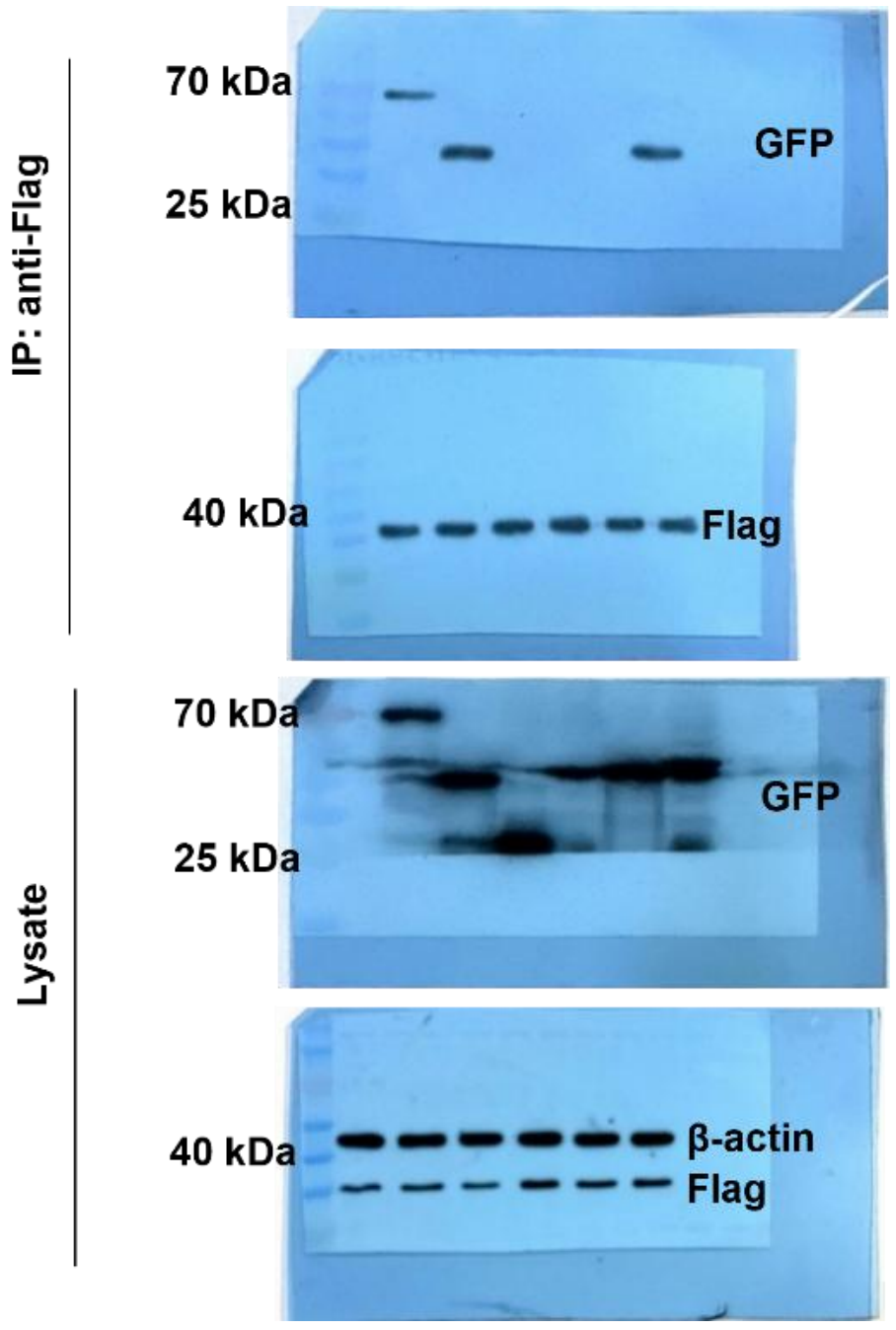

Supplementary Figure 6B

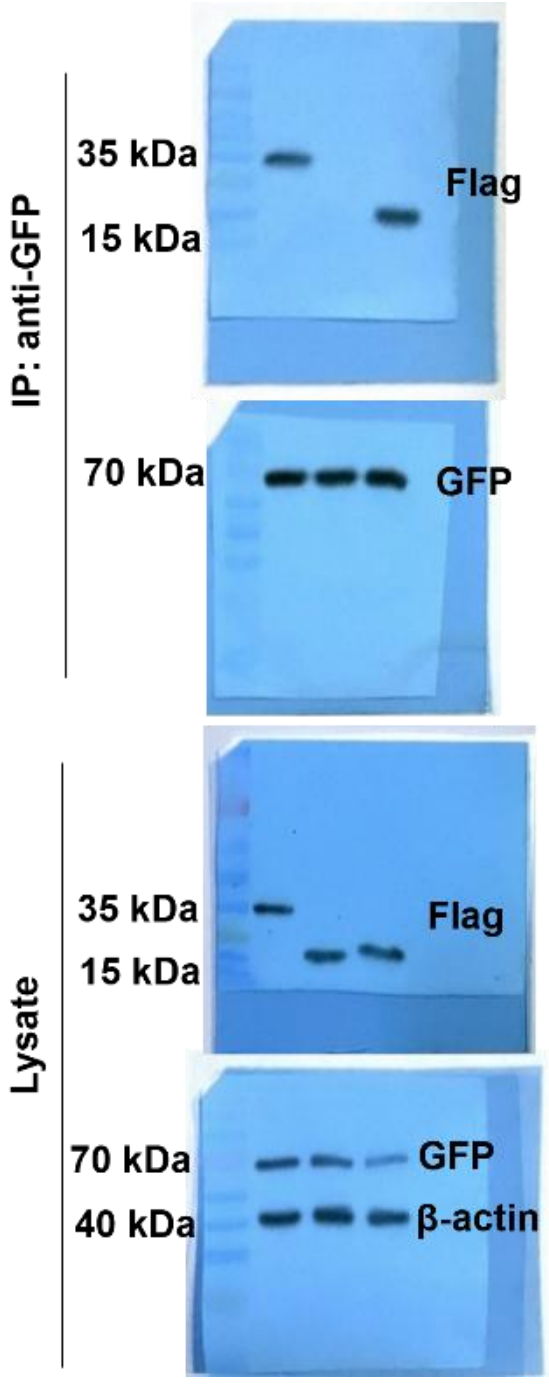

Supplementary Figure 6C

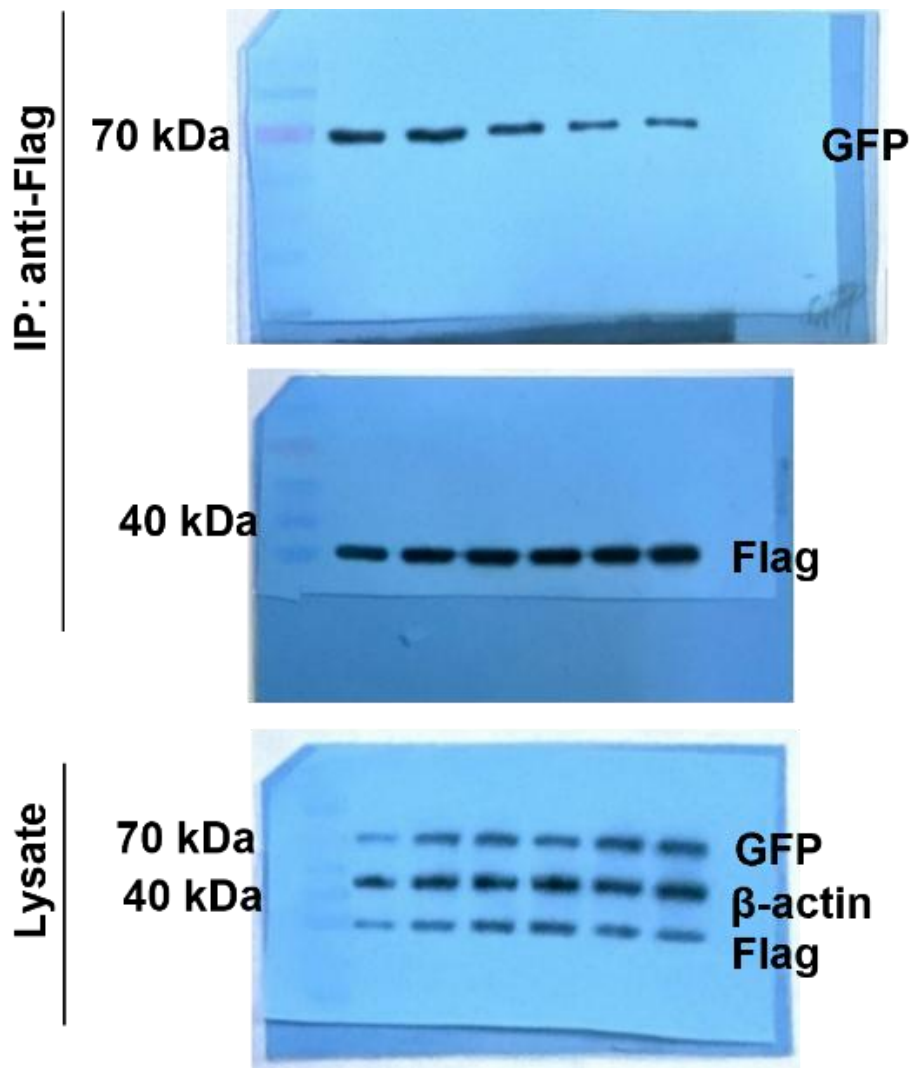

Supplementary Figure 6F

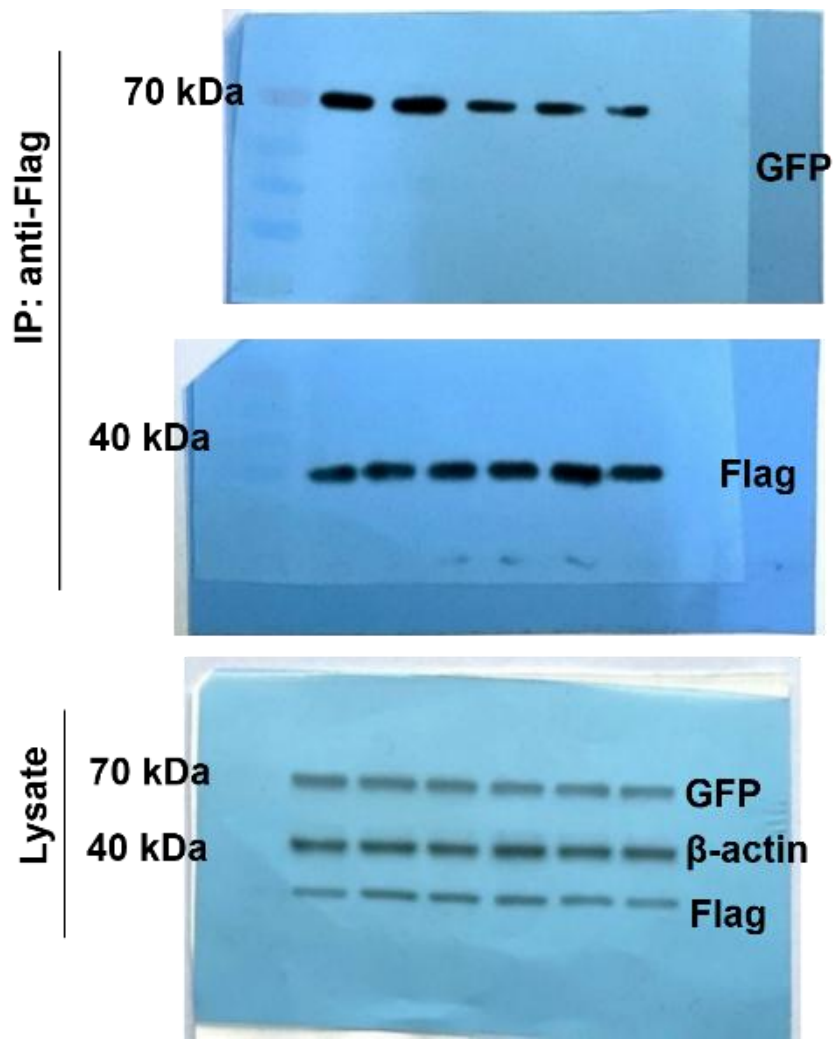

Supplementary Figure 6I

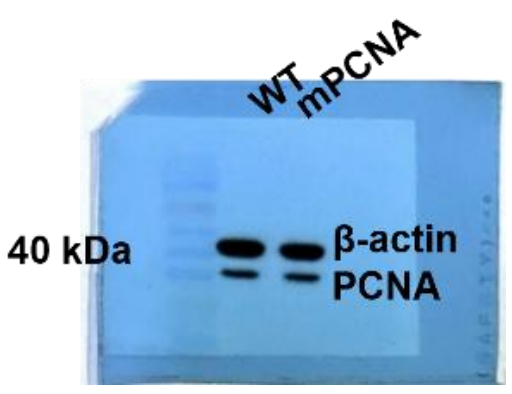

Supplementary Figure 6J

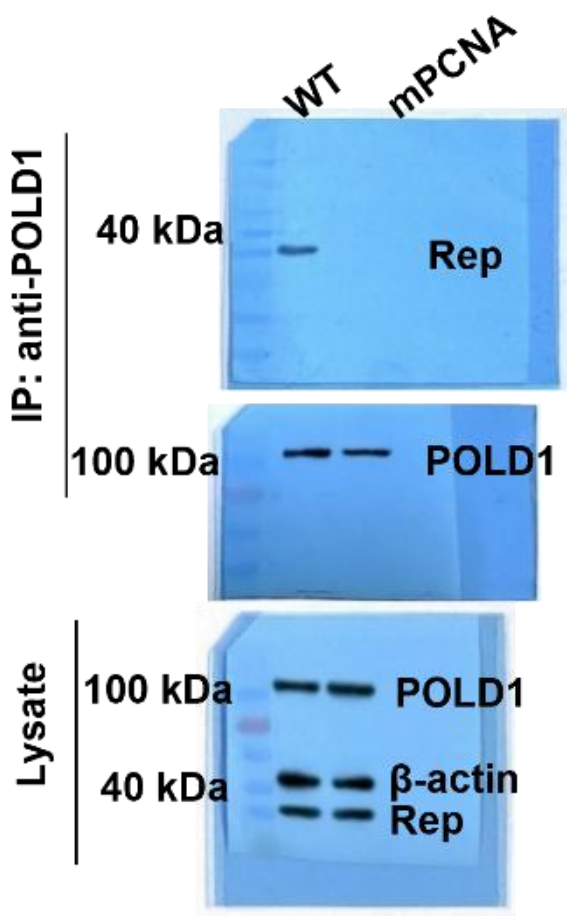

Supplementary Figure 8B

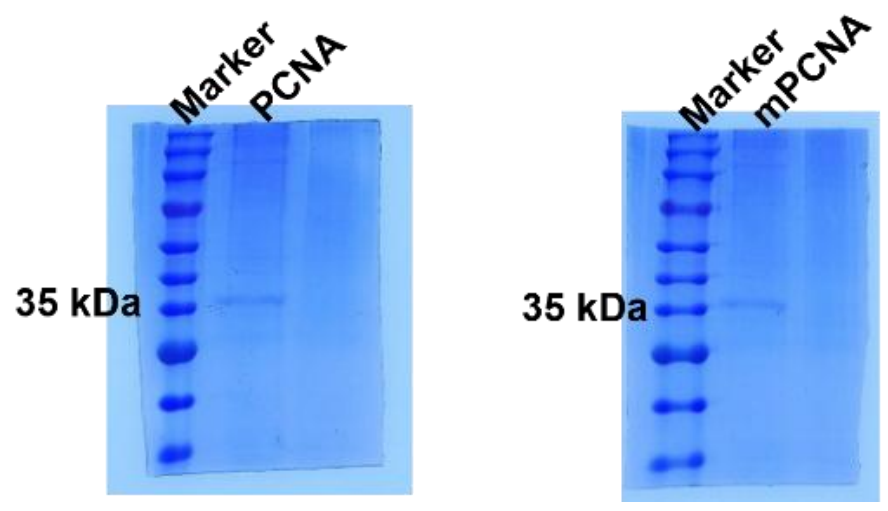

Supplementary Figure 8C

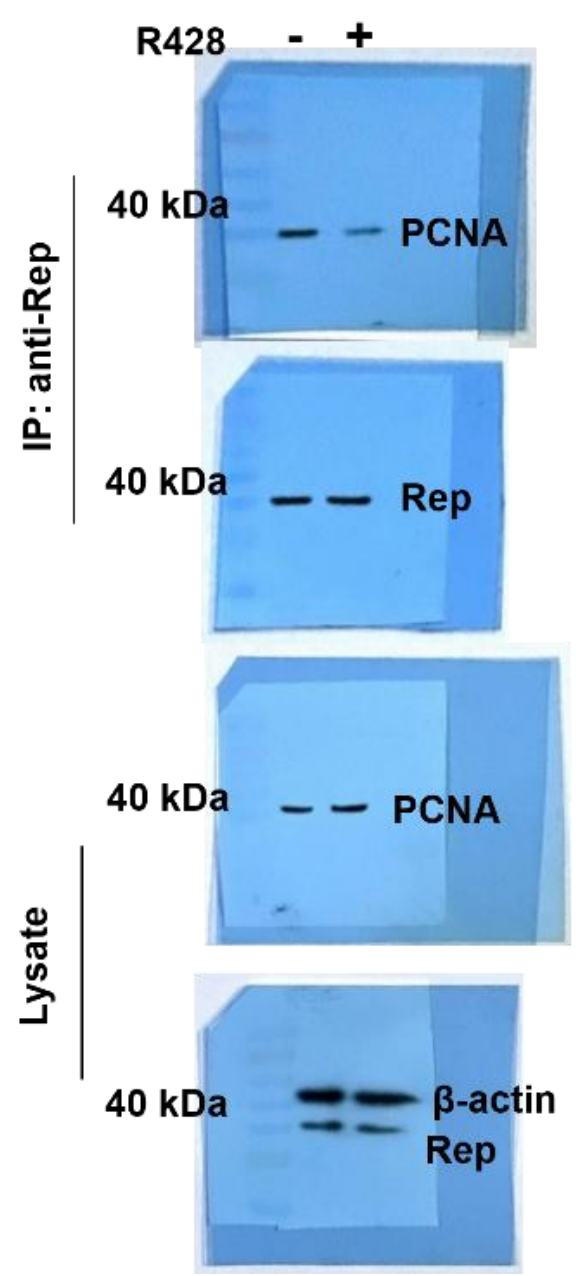

Supplementary Figure 8D

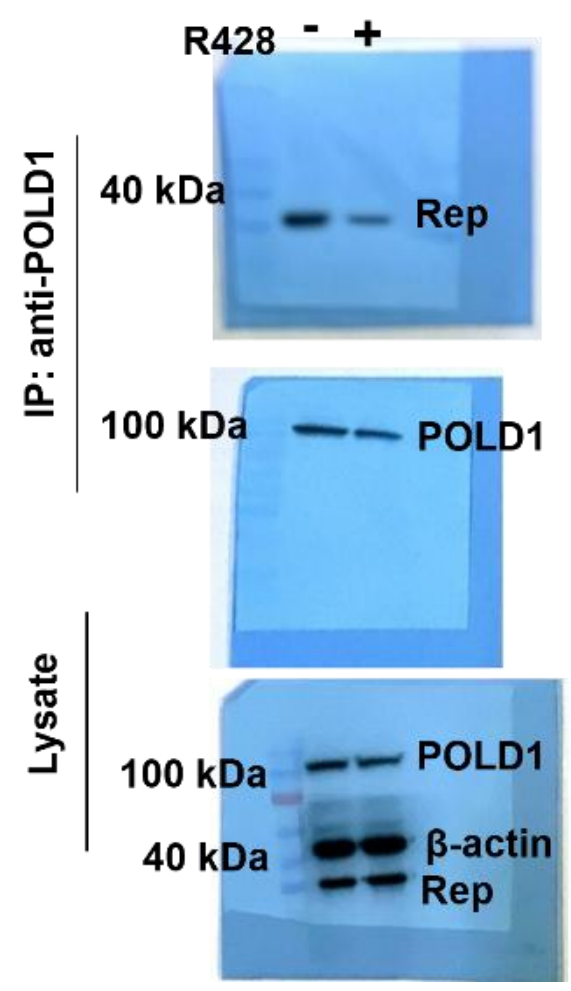

Supplementary Figure 8E

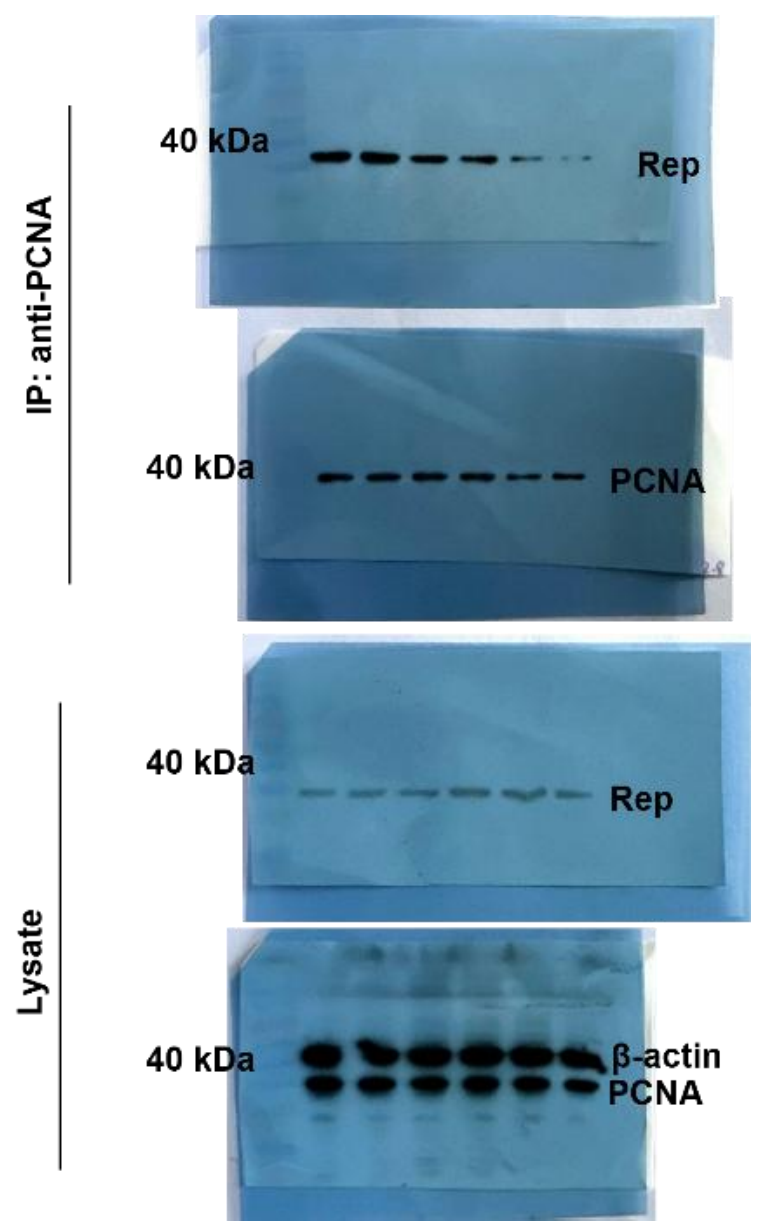

Supplementary Figure 8G

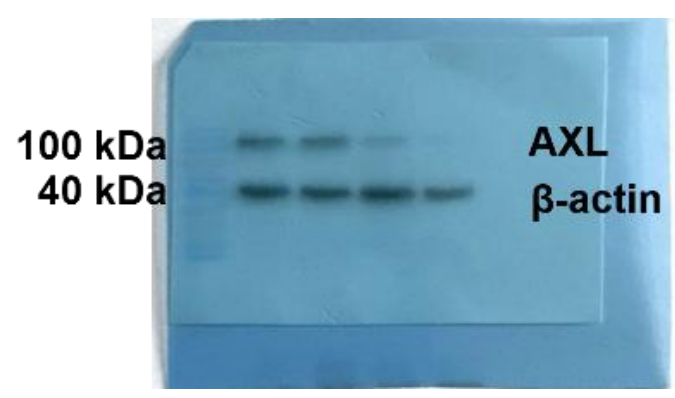

Supplement: Supplementary file 1 [file ppat.1014575.s002.pdf]
