# Supplementary material for: Hijacking of host PCNA by circovirus replication-associated protein to recruit POLD1 drives viral DNA replication and is inhibited by R428
Source: PLoS Pathog. 2026 Sep 28;22(9):e1014575. doi: 10.1371/journal.ppat.1014575 (PMC13618890; doi:10.1371/journal.ppat.1014575)
Supplement: Supplementary file 2 [file ppat.1014575.s001.docx]

**Supplementary Fig. 1.** (**A**) qPCR was performed to determine the effect of PCV2 interference on the mRNA expression of DNA polymerase. (**B**) PK-15 cells were transfected with sh-POLD1, followed by transfection with a Flag-tagged POLD1 plasmid for rescue, and then infected with PCV2 for 24 h, after which viral DNA copy number were quantified by qPCR. (**C**) PK-15 cells were infected with PCV2 or Mock for 12 h, and then ChIP assay was performed using POLD1 antibody to detect the interaction between PCV2 DNA and POLD1. (**D**) CCK-8 assay was used to investigate the effect of POLD1 interference on cell proliferation. (**E**) CCK-8 assay was used to investigate the effect of POLD1 overexpression on cell proliferation. ***p* < 0.01 versus the Mock group in (**A**); ***p* < 0.01 versus the shPOLD1 + Vector group in (**B**).

**Supplementary Fig. 2.** (**A, B**) qPCR was performed to quantify PCV2 loads in the spleen and lymph nodes of different piglets, and Western blot analysis was conducted to determine POLD1 expression in these tissues.

**Supplementary Fig. 3.** (**A**) PCV2 infection of PK-15 cells was performed for 12 h, and co-IP assay was performed to detect the interaction between the PCV2 Rep and POLD1. (**B**) co-IP assays were performed to detect the interaction between exogenously expressed POLD1 and the exogenously expressed Rep proteins from PCV2, PiCV, HirCV, and CaCV. 293T cells were co‑transfected with Flag‑tagged POLD1 plasmid and each GFP‑tagged Rep construct, with the empty GFP‑N1 vector used as a negative control. Immunoprecipitation was carried out using anti‑Flag magnetic beads, and the co‑precipitate proteins were detected by Western blotting with an anti‑GFP antibody. (**C**) co-IP assays were performed to detect the interaction between exogenously expressed POLD1 and the exogenously expressed Rep proteins from PCV2, PiCV, HirCV, and CaCV. 293T cells were co‑transfected with Flag‑tagged POLD1 plasmid and each GFP‑tagged Rep construct, with the empty GFP‑C1 vector used as a negative control. Immunoprecipitation was carried out using anti‑GFP magnetic beads, and the co‑precipitated proteins were detected by Western blotting with an anti‑Flag antibody. (**D**) The expression of Input proteins was detected by Coomassie Brilliant Blue staining.

**Supplementary Fig. 4.** (**A**) PCV2 infection of PK-15 cells was performed for 12 h, and co-IP assay was performed to detect the interaction between the PCV2 Rep and PCNA. (**B**) co-IP assays were performed to detect the interaction between exogenously expressed PCNA and the exogenously expressed Rep proteins from PCV2, PiCV, HirCV, and CaCV. 293T cells were co‑transfected with Flag‑tagged PCNA plasmid and each GFP‑tagged Rep construct, with the empty GFP‑N1 vector used as a negative control. Immunoprecipitation was carried out using anti‑Flag magnetic beads, and the co‑precipitated proteins were detected by Western blotting with an anti‑GFP antibody. (**C**) co-IP assays were performed to detect the interaction between exogenously expressed PCNA and the exogenously expressed Rep proteins from PCV2, PiCV, HirCV, and CaCV. 293T cells were co‑transfected with Flag‑tagged PCNA plasmid and each GFP‑tagged Rep construct, with the empty GFP‑C1 vector used as a negative control. Immunoprecipitation was carried out using anti‑GFP magnetic beads, and the co‑precipitated proteins were detected by Western blotting with an anti‑Flag antibody. (**D**) The expression of Input proteins was detected by Coomassie Brilliant Blue staining, and GST pull-down assays were further performed to validate the direct interaction between PCNA and the Rep proteins of PCV2, PiCV, HirCV, and CaCV. (**E**) The expression of Input proteins was detected by Coomassie Brilliant Blue staining, and GST pull-down assays were further performed to detect the effect of PCNA on the interaction between POLD1 and the Rep proteins of PCV2, PiCV, HirCV, and CaCV. (**F**) co-IP assays to detect the effect of PCNA interference on the interaction between POLD1 and the PCV2 Rep protein. Immunoprecipitation was carried out using anti‑POLD1 antibody, and the co‑precipitated proteins were detected by Western blotting with an anti‑Rep antibody.

**Supplementary Fig. 5.** (**A**) CCK-8 assay was used to investigate the effect of PCNA interference on cell proliferation. (**B**) The PCV2 TCID_50_ of these cells was measured and calculated. (**C**) The DNA copy numbers of PCV2 were detected and calculated by qPCR. (**D**) Positive cells were photographed using a fluorescence microscope. Bars = 100 μm are indicated in each panel, The counts of CP-positive and RFP-positive cells per 100 cells. (**E**) PK-15 cells transfected with PCV2 DNA‑WT or Rep‑deficient mutant (PCV2 DNA‑mut) were subjected to DNA pull‑down, and the bound proteins were detected by Western blotting with anti‑POLD1 and anti‑PCNA. (**F**) PK‑15 cells were transfected with PCV2 DNA‑WT or the Rep‑deficient mutant (PCV2 DNA‑mut), and the PCV2 DNA‑mut‑transfected cells were rescued with His‑Rep. DNA pull‑down assays were then performed, followed by immunoblotting with anti‑POLD1 and anti‑PCNA antibodies. ***p* < 0.01 versus the sh-NC group in (**B**), (**C**), (**D**).

**Supplementary Fig. 6.** (**A**) The plasmids expressing Flag-tag labeled PCNA as well as the GFP-tag labeled PCV2 Rep truncates were transfected into 293T cells for 24 h, and then the interaction of the PCNA with PCV2 Rep truncates were measured by co-IP assays. (**B**) The plasmids expressing Flag-tag labeled PCNA truncates as well as the GFP-tag labeled PCV2 Rep were transfected into 293T cells for 24 h, and then the interaction of the PCNA truncates with PCV2 Rep were measured by co-IP assays. (**C**) The plasmids expressing Flag-tag labeled PCNA as well as the GFP-tag labeled PCV2 Rep mutants were transfected into 293T cells for 24 h, and then the interaction of the PCNA with PCV2 Rep truncation mutants were measured by co-IP assays. (**D**) Quantitative analysis of Flag protein band intensities in the IP fractions was performed. (**E**) Quantitative analysis of GFP protein band intensities in the Input fractions was performed. (**F**) The plasmids expressing Flag-tag labeled PCNA mutants as well as the GFP-tag labeled PCV2 Rep were transfected into 293T cells for 24 h, and then the interaction of the PCNA mutants with PCV2 Rep were measured by co-IP assays. (**G**) Quantitative analysis of Flag protein band intensities in the IP fractions was performed. (**H**) Sequencing results of PK-15^WT^ and PK-15^mPCNA^ cells. (**I**) Western blotting detection of PCNA expression in PK-15^WT^ and PK-15^mPCNA^ cells. (**J**) PCV2 infection of PK-15^WT^ or PK-15^mPCNA^ cells was performed for 12 h, and co-IP assay was performed to detect the interaction between the PCV2 Rep and POLD1. (**K**) Prediction of the interaction site(s) Prediction of the interaction site(s) between PCV2 Rep, PiCV Rep, HirCV Rep, CaCV Rep and PCNA using AlphaFold 3.

**Supplementary Fig. 7.** (**A**) CCK-8 assay for the effects of different concentrations of the drug on cell viability. (**B**) qPCR analysis of the effects of different concentrations of the drug on PCV2 replication. **p* < 0.05 versus the DMSO group in (**B**).

**Supplementary Fig. 8.** (**A**) Prediction of R428–PCNA binding using AlphaFold 3. (**B**) SDS-PAGE analysis of the purified PCNA and mPCNA proteins. (**C**) co-IP assay for the effects of R428 on the interaction between PCV2 Rep and PCNA. (**D**) co-IP assay for the effects of R428 on the interaction between PCV2 Rep and POLD1. (**E**) Effects of different concentrations of R428 on the interaction between Rep and PCNA detected by co-IP assay. (**F**) Quantitative analysis of Rep protein band intensities in the IP fractions was performed. (**G**) The effects of AXL on PCV2 replication was examined by qPCR analysis. (**H**) CCK-8 assay was used to investigate the effect of AXL interference on cell proliferation. **p* < 0.05 versus the DMSO group in (**B**). **p* < 0.05 and ***p* < 0.01 versus the 0 group in (**F**). “ns” versus the sh-NC group in (**G**).
