# Supplementary material for: Hijacking of host PCNA by circovirus replication-associated protein to recruit POLD1 drives viral DNA replication and is inhibited by R428
Source: PLoS Pathog. 2026 Sep 28;22(9):e1014575. doi: 10.1371/journal.ppat.1014575 (PMC13618890; doi:10.1371/journal.ppat.1014575)
Supplement: Supplementary file 3 [file ppat.1014575.s008.docx]

**Supplementary Table 3 shRNA** **sequence (5’ to 3’)**

| **name** | **sequence** **(5’ to 3’)** |
| --- | --- |
| shPOLA1-1-F | GATCCGGACCAATACATCCAGCTTAGCTCGAGCTAAGCTGGATGTATTGGTCCTTTTTG |
| shPOLA1-1-R | AATTCAAAAAGGACCAATACATCCAGCTTAGCTCGAGCTAAGCTGGATGTATTGGTCCG |
| shPOLA1-2-F | GATCCGCATAAGTGTTAAGCTCAAGTCTCGAGACTTGAGCTTAACACTTATGCTTTTTG |
| shPOLA1-2-R | AATTCAAAAAGCATAAGTGTTAAGCTCAAGTCTCGAGACTTGAGCTTAACACTTATGCG |
| shPOLB-1-F | GATCCCCAAAGCTGCTACATCGTGTTCTCGAGAACACGATGTAGCAGCTTTGGTTTTTG |
| shPOLB-1-R | AATTCAAAAACCAAAGCTGCTACATCGTGTTCTCGAGAACACGATGTAGCAGCTTTGGG |
| shPOLB-2-F | GATCCTCCACACAGGAGAATCGATATCTCGAGATATCGATTCTCCTGTGTGGATTTTTG |
| shPOLB-2-R | AATTCAAAAATCCACACAGGAGAATCGATATCTCGAGATATCGATTCTCCTGTGTGGAG |
| shPOLD1-1-F | GATCCGCAGCTGGAGATCGACCATTACTCGAGTAATGGTCGATCTCCAGCTGCTTTTTG |
| shPOLD1-1-R | AATTCAAAAAGCAGCTGGAGATCGACCATTACTCGAGTAATGGTCGATCTCCAGCTGCG |
| shPOLD1-2-F | GATCCGCTTTGCACCCTACTTCTACACTCGAGTGTAGAAGTAGGGTGCAAAGCTTTTTG |
| shPOLD1-2-R | AATTCAAAAAGCTTTGCACCCTACTTCTACACTCGAGTGTAGAAGTAGGGTGCAAAGCG |
| shPOLE1-1-F | GATCCGCGTTTGACATTGAGACAACCCTCGAGGGTTGTCTCAATGTCAAACGCTTTTTG |
| shPOLE1-1-R | AATTCAAAAAGCGTTTGACATTGAGACAACCCTCGAGGGTTGTCTCAATGTCAAACGCG |
| shPOLE1-2-F | GATCCGCTCCAACATGGTCTATAACCCTCGAGGGTTATAGACCATGTTGGAGCTTTTTG |
| shPOLE1-2-R | AATTCAAAAAGCTCCAACATGGTCTATAACCCTCGAGGGTTATAGACCATGTTGGAGCG |
| shPOLK-1-F | GATCCGCAAGTCAACCAACGAATTGACTCGAGTCAATTCGTTGGTTGACTTGCTTTTTG |
| shPOLK-1-R | AATTCAAAAAGCAAGTCAACCAACGAATTGACTCGAGTCAATTCGTTGGTTGACTTGCG |
| shPOLK-2-F | GATCCGCACATTGCTATCCTCAAACACTCGAGTGTTTGAGGATAGCAATGTGCTTTTTG |
| shPOLK-2-R | AATTCAAAAAGCACATTGCTATCCTCAAACACTCGAGTGTTTGAGGATAGCAATGTGCG |
| shPOLQ-1-F | GATCCGCATCTTGCCAGGCAGATTTACTCGAGTAAATCTGCCTGGCAAGATGCTTTTTG |
| shPOLQ-1-R | AATTCAAAAAGCATCTTGCCAGGCAGATTTACTCGAGTAAATCTGCCTGGCAAGATGCG |
| shPOLQ-2-F | GATCCGCAACAAGCAAAGCAGATTTGCTCGAGCAAATCTGCTTTGCTTGTTGCTTTTTG |
| shPOLQ-2-R | AATTCAAAAAGCAACAAGCAAAGCAGATTTGCTCGAGCAAATCTGCTTTGCTTGTTGCG |
| shREV3L-1-F | GATCCGCTTTAGGCAAACCATCTTCCCTCGAGGGAAGATGGTTTGCCTAAAGCTTTTTG |
| shREV3L-1-R | AATTCAAAAAGCTTTAGGCAAACCATCTTCCCTCGAGGGAAGATGGTTTGCCTAAAGCG |
| shREV3L-2-F | GATCCGGAGATGACAGACCCTCATCACTCGAGTGATGAGGGTCTGTCATCTCCTTTTTG |
| shREV3L-2-R | AATTCAAAAAGGAGATGACAGACCCTCATCACTCGAGTGATGAGGGTCTGTCATCTCCG |
| shAXL-1-F | GATCCGCCCATAATGCCAAGGGGGTCCTCGAGGACCCCCTTGGCATTATGGGCTTTTTG |
| shAXL-1-R | AATTCAAAAAGCCCATAATGCCAAGGGGGTCCTCGAGGACCCCCTTGGCATTATGGGCG |
| shAXL-2-F | GATCCGCCGTGAAGACGATGAAGATTCTCGAGAATCTTCATCGTCTTCACGGCTTTTTG |
| shAXL-2-R | AATTCAAAAAGCCGTGAAGACGATGAAGATTCTCGAGAATCTTCATCGTCTTCACGGCG |
| shPCNA-1-F | GATCCCTGGCAATGAAGACATCATCTCGAGATGATGTCTTCATTGCCAGTTTTTG |
| shPCNA-1-R | AATTCAAAAACTGGCAATGAAGACATCATCTCGAGATGATGTCTTCATTGCCAGG |
| shPCNA-2-F | GATCCGGAGGAAGCAGTTACCATACTCGAGTATGGTAACTGCTTCCTCCTTTTTG |
| shPCNA-2-R | AATTCAAAAAGGAGGAAGCAGTTACCATACTCGAGTATGGTAACTGCTTCCTCCG |

The table lists all shRNA sequences used in this study, including shRNAs targeting key subunits of DNA polymerases (POLA1, POLB, POLD1, POLE1, POLK, POLQ, REV3L), AXL kinase, and PCNA. Each gene was targeted by two independent shRNA constructs (shRNA-1 and shRNA-2), each consisting of a forward (F) and a reverse (R) oligonucleotide.
